# Supplementary material for: Household and area determinants of emergency department attendance and hospitalisation in people with multimorbidity: a systematic review
Source: BMJ Open. 2022 Oct 3;12(10):e063441. doi: 10.1136/bmjopen-2022-063441 (PMC9535173; doi:10.1136/bmjopen-2022-063441)
Supplement: Supplementary data [file bmjopen-2022-063441supp001.pdf]

Supplementary File

Table S1. Database search strategies

Database (number of articles)	Search strategy
MedLine (1659)	(MULTIMORBIDITY/ or "multimorbidit*".mp. or "multi-morbidit*".mp. or "comorbidit*".mp. or "co-morbidit*".mp. or "polymorbidit*".mp. or "poly-morbidit*".mp. or"multicondition*".mp. or "multi-condition*".mp. or "multiple chronic condition*".mp. or "morbidity burden*".mp. or ((multiple or coexisting or co-existing or concurrent or con-current or comorbid* or co-morbid* or several*) adj2 (disease* or illness* or condition* or diagnosis* or morbid* or patholog*))) AND ("household*".mp. or "coresiden*".mp. or "co-residen*".mp. or ((household or family or domestic or residen*) adj2 (composition* or member* or characterist* or dweller*)) or "neighborhood*".mp. or "neighbourhood*".mp. or "small area*".mp. or "small-area*".mp. or "place".mp. or "communit*".mp. or "census tracts".mp. or "census block group".mp. or "zip code*".mp. or "post code*".mp. or "electoral ward*".mp. or "data zone*".mp. or "output area*".mp. or "lower super output area*".mp. or "municipalit*".mp. or "provinc*".mp. or SOCIOECONOMIC FACTORS/ or INCOME/ or "socioeconomic".mp. or "socio-economic".mp. or "social capital".mp. or "inequalit*".mp. or "disparit*".mp. or "income".mp. or "wealth".mp. or "financial problem*".mp. or "financial difficult*".mp. or "vocation*".mp. or "training opportunit*".mp. or "employment opportunit*".mp. or "occupation*".mp. or "opportunit*".mp. or "job opportunit*".mp. or "job insecurit".mp. or "education*" or "opportunit*".mp. or "education*".mp. or "achiev*".mp. or "education*".mp. or "quality".mp. or "fast-food".mp. or "fast food".mp. or "healthy food".mp. or "social determinant".mp. or "wider determinant".mp. or POVERTY AREAS/ or POVERTY/ or "neighbourhood disorder".mp. or "overcrowding".mp. or "public housing".mp. or "population density".mp. or "urban".mp. or "ghetto".mp. or "rural".mp. or "slum".mp. or "estate".mp. or "poverty area*".mp. or "physical environment*".mp. or "built environment*".mp. or "living standard*".mp. or "family structure".mp. or "family breakdown".mp. or "family disintegrat*".mp. or "single parent*".mp. or "housing tenure".mp. or "Townsend".mp. or "Carstairs".mp. or "Index of Multiple Deprivation".mp. or "Nam-Powers".mp. or "Hollingshead Index".mp. or "Breadline Britain Index".mp. or "inverse care law".mp. or "Singh ind*".mp. or "Yost ind*".mp. or "Kolak ind*".mp. or "social determinants of health".mp. or "neighbourhood archetype*".mp. or "segregation indic*".mp. or "ecological inversion".mp. or "ecological fallacy".mp. or "ecological bias".mp. or "depriv*".mp. or "impoverish*".mp. or "disadvantage*".mp. or "community cohesion".mp. or "community network".mp. or "community support".mp. or "transport* quality".mp. or "transport* services".mp. or "transport link*".mp. or "public transport*".mp. or "travel time".mp. or "community resource access*".mp. or "walkability".mp. or "public space".mp. or "green space".mp. or "open space".mp. or "recreation*".mp. or "leisure activit*".mp. or "food outlet*".mp. or "food environment".mp. or "air pollution".mp. or "particulate matter".mp. or "nitrogen dioxide".mp. or "sulphur dioxide".mp. or "sulfur dioxide".mp. or "carbon monoxide".mp. or "crime*".mp.) AND ("hospital admission*".mp. or "hospitalisation".mp. or "hospitalization".mp. or "emergency admission*".mp. or "unscheduled admission*".mp. or "unplanned admission*".mp. or "emergency department".mp. or "accident and emergency".mp.)<br>limit to (english language and yr="2000-Current")
EMBASE (5549)	("multimorbidit*".mp. or "multi-morbidit*".mp. or "comorbidit*".mp. or "co-morbidit*".mp. or "polymorbidit*".mp. or "poly-morbidit*".mp. or"multicondition*".mp. or "multi-condition*".mp. or "multiple chronic condition*".mp. or "morbidity burden*".mp. or "multiple long-term condition*".mp. or ((multiple or coexisting or co-existing or concurrent or con-current or comorbid* or co-morbid* or several*) adj2 (disease* or illness* or condition* or diagnosis* or morbid* or patholog*))) AND ("household*".mp. or "coresiden*".mp. or "co-residen*".mp. or ((household or family or domestic or residen*) adj2 (composition* or member* or characterist* or dweller*)) or "neighborhood*".mp. or "neighbourhood*".mp. or "small area*".mp. or "small-area*".mp. or "place".mp. or "communit*".mp. or "census tracts".mp. or "census block group".mp. or "zip code*".mp. or "post code*".mp. or "electoral ward*".mp. or "data zone*".mp. or "output area*".mp. or "lower super output area*".mp. or "municipalit*".mp. or "provinc*".mp. or "socioeconomic".mp. or "socio-economic".mp. or "social capital".mp. or "inequalit*".mp. or "disparit*".mp. or "income".mp. or "wealth".mp. or "financial problem*".mp. or "financial difficult*".mp. or "vocation*".mp. or "training opportunit*".mp. or "employment opportunit*".mp. or "occupation*".mp. or "opportunit*".mp. or "job opportunit*".mp. or "job insecurit".mp. or "education*" or "opportunit*".mp. or "education*".mp. or "achiev*".mp. or "education*".mp. or "quality".mp. or "fast-food".mp. or "fast food".mp. or "healthy food".mp. or "social determinant".mp. or "wider determinant".mp. or "neighbourhood disorder".mp. or "overcrowding".mp. or "public housing".mp. or "population density".mp. or "urban".mp. or "ghetto".mp. or "rural".mp. or "slum".mp. or "estate".mp. or "poverty area*".mp. or "physical environment*".mp. or "built environment*".mp. or "living standard*".mp. or "family structure".mp. or "family breakdown".mp. or "family disintegrat*".mp. or "single parent*".mp. or "housing tenure".mp. or "Townsend".mp. or "Carstairs".mp. or "Index of Multiple Deprivation".mp. or "Nam-Powers".mp. or "Hollingshead Index".mp. or "Breadline Britain Index".mp. or "inverse care law".mp. or "Singh ind*".mp. or "Yost ind*".mp. or "Kolak ind*".mp. or "social determinants of health".mp. or "neighbourhood archetype*".mp. or "segregation indic*".mp. or "ecological inversion".mp. or "ecological fallacy".mp. or "ecological bias".mp. or "depriv*".mp. or "impoverish*".mp. or "disadvantage*".mp. or "community cohesion".mp. or "community network".mp. or "community support".mp. or "transport* quality".mp. or "transport* services".mp. or "transport link*".mp. or "public transport*".mp. or "travel time".mp. or "community resource access*".mp. or "walkability".mp. or "public space".mp. or "green space".mp. or "open space".mp. or "recreation*".mp. or "leisure activit*".mp. or "food

	outlet*".mp. or "food environment".mp. or "air pollution".mp. or "particulate matter".mp. or "nitrogen dioxide".mp. or "sulphur dioxide".mp. or "sulfur dioxide".mp. or "carbon monoxide".mp. or "crime*".mp.) AND ("admission*".mp. or "hospital admission*".mp. or "hospitalisation".mp. or "hospitalization".mp. or "emergency admission*".mp. or "unscheduled admission*".mp. or "unplanned admission*".mp. or "emergency department".mp. or "accident and emergency".mp.)<br>limit to (english language and yr="2000-Current")
Global Health (556)	("multimorbidit*".mp. or "multi-morbidit*".mp. or "comorbidit*".mp. or "co-morbidit*".mp. or "polymorbidit*".mp. or "poly-morbidit*".mp. or"multicondition*".mp. or "multi-condition*".mp. or "multiple chronic condition*".mp. or "morbidity burden*".mp. or ((multiple or coexisting or co-existing or concurrent or con-current or comorbid* or co-morbid* or several*) adj2 (disease* or illness* or condition* or diagnosis* or morbid* or patholog*))) AND ("household*".mp. or "coresiden*".mp. or "co-residen*".mp. or ((household or family or domestic or residen*) adj2 (composition* or member* or characterist* or dweller*)) or "neighborhood*".mp. or "neighbourhood*".mp. or "small area*".mp. or "small-area*".mp. or "place".mp. or "communit*".mp. or "census tracts".mp. or "census block group".mp. or "zip code*".mp. or "post code*".mp. or "electoral ward*".mp. or "data zone*".mp. or "output area*".mp. or "lower super output area*".mp. or "municipalit*".mp. or "provinc*".mp. or "socioeconomic".mp. or "socio-economic".mp. or "social capital".mp. or "inequalit*".mp. or "disparit*".mp. or "income".mp. or "wealth".mp. or "financial problem*".mp. or "financial difficult*".mp. or "vocation*".mp. or "training opportunit*".mp. or "employment opportunit*".mp. or "occupation*".mp. or "opportunit*".mp. or "job opportunit*".mp. or "job insecurit".mp. or "education*" or "opportunit*".mp. or "education*".mp. or "achiev*".mp. or "education*".mp. or "quality".mp. or "fast-food".mp. or "fast food".mp. or "healthy food".mp. or "social determinant".mp. or "wider determinant".mp. or "neighbourhood disorder".mp. or "overcrowding".mp. or "public housing".mp. or "population density".mp. or "urban".mp. or "ghetto".mp. or "rural".mp. or "slum".mp. or "estate".mp. or "poverty area*".mp. or "physical environment*".mp. or "built environment*".mp. or "living standard*".mp. or "family structure".mp. or "family breakdown".mp. or "family disintegrat*".mp. or "single parent*".mp. or "housing tenure".mp. or "Townsend".mp. or "Carstairs".mp. or "Index of Multiple Deprivation".mp. or "Nam-Powers".mp. or "Hollingshead Index".mp. or "Breadline Britain Index".mp. or "inverse care law".mp. or "Singh ind*".mp. or "Yost ind*".mp. or "Kolak ind*".mp. or "social determinants of health".mp. or "neighbourhood archetype*".mp. or "segregation indic*".mp. or "ecological inversion".mp. or "ecological fallacy".mp. or "ecological bias".mp. or "depriv*".mp. or "impoverish*".mp. or "disadvantage*".mp. or "community cohesion".mp. or "community network".mp. or "community support".mp. or "transport* quality".mp. or "transport* services".mp. or "transport link*".mp. or "public transport*".mp. or "travel time".mp. or "community resource access*".mp. or "walkability".mp. or "public space".mp. or "green space".mp. or "open space".mp. or "recreation*".mp. or "leisure activit*".mp. or "food outlet*".mp. or "food environment".mp. or "air pollution".mp. or "particulate matter".mp. or "nitrogen dioxide".mp. or "sulphur dioxide".mp. or "sulfur dioxide".mp. or "carbon monoxide".mp. or "crime*".mp.) AND ("hospital admission*".mp. or "hospitalisation".mp. or "hospitalization".mp. or "emergency admission*".mp. or "unscheduled admission*".mp. or "unplanned admission*".mp. or "emergency department".mp. or "accident and emergency".mp.)<br>limit to (english language and yr="2000-Current")
PsychInfo (283)	("multimorbidit*".mp. or "multi-morbidit*".mp. or "comorbidit*".mp. or "co-morbidit*".mp. or "polymorbidit*".mp. or "poly-morbidit*".mp. or"multicondition*".mp. or "multi-condition*".mp. or "multiple chronic condition*".mp. or "morbidity burden*".mp. or ((multiple or coexisting or co-existing or concurrent or con-current or comorbid* or co-morbid* or several*) adj2 (disease* or illness* or condition* or diagnosis* or morbid* or patholog*))) AND ("household*".mp. or "coresiden*".mp. or "co-residen*".mp. or ((household or family or domestic or residen*) adj2 (composition* or member* or characterist* or dweller*)) or "neighborhood*".mp. or "neighbourhood*".mp. or "small area*".mp. or "small-area*".mp. or "place".mp. or "communit*".mp. or "census tracts".mp. or "census block group".mp. or "zip code*".mp. or "post code*".mp. or "electoral ward*".mp. or "data zone*".mp. or "output area*".mp. or "lower super output area*".mp. or "municipalit*".mp. or "provinc*".mp. or "socioeconomic".mp. or "socio-economic".mp. or "social capital".mp. or "inequalit*".mp. or "disparit*".mp. or "income".mp. or "wealth".mp. or "financial problem*".mp. or "financial difficult*".mp. or "vocation*".mp. or "training opportunit*".mp. or "employment opportunit*".mp. or "occupation*".mp. or "opportunit*".mp. or "job opportunit*".mp. or "job insecurit".mp. or "education*" or "opportunit*".mp. or "education*".mp. or "achiev*".mp. or "education*".mp. or "quality".mp. or "fast-food".mp. or "fast food".mp. or "healthy food".mp. or "social determinant".mp. or "wider determinant".mp. or "neighbourhood disorder".mp. or "overcrowding".mp. or "public housing".mp. or "population density".mp. or "urban".mp. or "ghetto".mp. or "rural".mp. or "slum".mp. or "estate".mp. or "poverty area*".mp. or "physical environment*".mp. or "built environment*".mp. or "living standard*".mp. or "family structure".mp. or "family breakdown".mp. or "family disintegrat*".mp. or "single parent*".mp. or "housing tenure".mp. or "Townsend".mp. or "Carstairs".mp. or "Index of Multiple Deprivation".mp. or "Nam-Powers".mp. or "Hollingshead Index".mp. or "Breadline Britain Index".mp. or "inverse care law".mp. or "Singh ind*".mp. or "Yost ind*".mp. or "Kolak ind*".mp. or "social determinants of health".mp. or "neighbourhood archetype*".mp. or "segregation indic*".mp. or "ecological inversion".mp. or "ecological fallacy".mp. or "ecological bias".mp. or "depriv*".mp. or "impoverish*".mp. or "disadvantage*".mp. or "community cohesion".mp. or "community network".mp. or "community support".mp. or "transport* quality".mp. or "transport* services".mp. or "transport link*".mp. or "public transport*".mp. or "travel time".mp. or "community resource access*".mp. or "walkability".mp. or "public space".mp. or "green space".mp. or "open space".mp. or "recreation*".mp. or "leisure activit*".mp. or "food outlet*".mp. or "food environment".mp. or "air pollution".mp. or "particulate matter".mp. or "nitrogen dioxide".mp. or "sulphur dioxide".mp. or "sulfur dioxide".mp. or "carbon monoxide".mp. or "crime*".mp.) AND

	("hospital admission*".mp. or "hospitalisation".mp. or "hospitalization".mp. or "emergency admission*".mp. or "unscheduled admission*".mp. or "unplanned admission*".mp. or "emergency department".mp. or "accident and emergency".mp.)<br>limit to (english language and yr="2000-Current")
ASSIA (380)	("multimorbidit*" or "multi-morbidit*" or "comorbidit*" or "co-morbidit*" or "polymorbidit*" or "poly-morbidit*" or "multicondition*" or "multi-condition*" or "multiple chronic condition*" or "morbidity burden*" or ((multiple or coexisting or co-existing or concurrent or con-current or comorbid* or co-morbid* or several*) adj2 (disease* or illness* or condition* or diagnosis* or morbid* or patholog*))) AND ("household*" or "coresiden*" or "co-residen*" or ((household or family or domestic or residen*) adj2 (composition* or member* or characterist* or dweller*)) or "neighborhood*" or "neighbourhood*" or "small area*" or "small-area*" or "place" or "communit*" or "census tracts" or "census block group" or "zip code*" or "post code*" or "electoral ward*" or "data zone*" or "output area*" or "lower super output area*" or "municipalit*" or "provinc*" or "socioeconomic" or "socio-economic" or "social capital" or "inequalit*" or "disparit*" or "income" or "wealth" or "financial problem*" or "financial difficult*" or "vocation*" or "training opportunit*" or "employment opportunit*" or "occupation* opportunit*" or "job opportunit*" or "job insecurit" or "education* opportunit*" or "education* achiev*" or "education* quality" or "fast-food" or "fast food" or "healthy food" or "social determinant" or "wider determinant" or "neighbourhood disorder" or "overcrowding" or "public housing" or "population density" or "urban" or "ghetto" or "rural" or "slum" or "estate" or "poverty area*" or "physical environment*" or "built environment*" or "living standard*" or "family structure" or "family breakdown" or "family disintegrat*" or "single parent*" or "housing tenure" or "Townsend" or "Carstairs" or "Index of Multiple Deprivation" or "Nam-Powers" or "Hollingshead Index" or "Breadline Britain Index" or "inverse care law" or "Singh ind*" or "Yost ind*" or "Kolak ind*" or "social determinants of health" or "neighbourhood archetype*" or "segregation indic*" or "ecological inversion" or "ecological fallacy" or "ecological bias" or "depriv*" or "impoverish*" or "disadvantage*" or "community cohesion" or "community network" or "community support" or "transport* quality" or "transport* services" or "transport link*" or "public transport*" or "travel time" or "community resource access*" or "walkability" or "public space" or "green space" or "open space" or "recreation*" or "leisure activit*" or "food outlet*" or "food environment" or "air pollution" or "particulate matter" or "nitrogen dioxide" or "sulphur dioxide" or "sulfur dioxide" or "carbon monoxide" or "crime*") AND ("hospital admission*" or "hospitalisation" or "hospitalization" or "emergency admission*" or "unscheduled admission*" or "unplanned admission*" or "emergency department" or "accident and emergency")<br>Publication date: after 01 January 2000
CAB Abstracts (142)	("multimorbidit*" or "multi-morbidit*" or "comorbidit*" or "co-morbidit*" or "polymorbidit*" or "poly-morbidit*" or "multicondition*" or "multi-condition*" or "multiple chronic condition*" or "morbidity burden*" or ((multiple or coexisting or co-existing or concurrent or con-current or comorbid* or co-morbid* or several*) adj2 (disease* or illness* or condition* or diagnosis* or morbid* or patholog*))) AND ("household*" or "coresiden*" or "co-residen*" or ((household or family or domestic or residen*) adj2 (composition* or member* or characterist* or dweller*)) or "neighborhood*" or "neighbourhood*" or "small area*" or "small-area*" or "place" or "communit*" or "census tracts" or "census block group" or "zip code*" or "post code*" or "electoral ward*" or "data zone*" or "output area*" or "lower super output area*" or "municipalit*" or "provinc*" or "socioeconomic" or "socio-economic" or "social capital" or "inequalit*" or "disparit*" or "income" or "wealth" or "financial problem*" or "financial difficult*" or "vocation*" or "training opportunit*" or "employment opportunit*" or "occupation* opportunit*" or "job opportunit*" or "job insecurit" or "education* opportunit*" or "education* achiev*" or "education* quality" or "fast-food" or "fast food" or "healthy food" or "social determinant" or "wider determinant" or "neighbourhood disorder" or "overcrowding" or "public housing" or "population density" or "urban" or "ghetto" or "rural" or "slum" or "estate" or "poverty area*" or "physical environment*" or "built environment*" or "living standard*" or "family structure" or "family breakdown" or "family disintegrat*" or "single parent*" or "housing tenure" or "Townsend" or "Carstairs" or "Index of Multiple Deprivation" or "Nam-Powers" or "Hollingshead Index" or "Breadline Britain Index" or "inverse care law" or "Singh ind*" or "Yost ind*" or "Kolak ind*" or "social determinants of health" or "neighbourhood archetype*" or "segregation indic*" or "ecological inversion" or "ecological fallacy" or "ecological bias" or "depriv*" or "impoverish*" or "disadvantage*" or "community cohesion" or "community network" or "community support" or "transport* quality" or "transport* services" or "transport link*" or "public transport*" or "travel time" or "community resource access*" or "walkability" or "public space" or "green space" or "open space" or "recreation*" or "leisure activit*" or "food outlet*" or "food environment" or "air pollution" or "particulate matter" or "nitrogen dioxide" or "sulphur dioxide" or "sulfur dioxide" or "carbon monoxide" or "crime*") AND ("hospital admission*" or "hospitalisation" or "hospitalization" or "emergency admission*" or "unscheduled admission*" or "unplanned admission*" or "emergency department" or "accident and emergency")<br>Publication date: after 01 January 2000
Web of Science (4071)	("multimorbidit*" or "multi-morbidit*" or "comorbidit*" or "co-morbidit*" or "polymorbidit*" or "poly-morbidit*" or "multicondition*" or "multi-condition*" or "multiple chronic condition*" or "morbidity burden*" or ((multiple or coexisting or co-existing or concurrent or con-current or comorbid* or co-morbid* or several*) adj2 (disease* or illness* or condition* or diagnosis* or morbid* or patholog*))) AND ("household*" or "coresiden*" or "co-residen*" or ((household or family or domestic or residen*) adj2 (composition* or member* or characterist* or dweller*)) or "neighborhood*" or "neighbourhood*" or "small area*" or "small-area*" or "place" or "communit*" or "census tracts" or "census block group" or "zip code*" or "post code*" or "electoral ward*" or "data zone*" or

	"output area*" or "lower super output area*" or "municipalit*" or "provinc*" or "socioeconomic" or "socio-economic" or "social capital" or "inequalit*" or "disparit*" or "income" or "wealth" or "financial problem*" or "financial difficult*" or "vocation*" or "training opportunit*" or "employment opportunit*" or "occupation* opportunit*" or "job opportunit*" or "job insecurit" or "education* opportunit*" or "education* achiev*" or "education* quality" or "fast-food" or "fast food" or "healthy food" or "social determinant" or "wider determinant" or "neighbourhood disorder" or "overcrowding" or "public housing" or "population density" or "urban" or "ghetto" or "rural" or "slum" or "estate" or "poverty area*" or "physical environment*" or "built environment*" or "living standard*" or "family structure" or "family breakdown" or "family disintegrat*" or "single parent*" or "housing tenure" or "Townsend" or "Carstairs" or "Index of Multiple Deprivation" or "Nam-Powers" or "Hollingshead Index" or "Breadline Britain Index" or "inverse care law" or "Singh ind*" or "Yost ind*" or "Kolak ind*" or "social determinants of health" or "neighbourhood archetype*" or "segregation indic*" or "ecological inversion" or "ecological fallacy" or "ecological bias" or "depriv*" or "impoverish*" or "disadvantage*" or "community cohesion" or "community network" or "community support" or "transport* quality" or "transport* services" or "transport link*" or "public transport*" or "travel time" or "community resource access*" or "walkability" or "public space" or "green space" or "open space" or "recreation*" or "leisure activit*" or "food outlet*" or "food environment" or "air pollution" or "particulate matter" or "nitrogen dioxide" or "sulphur dioxide" or "sulfur dioxide" or "carbon monoxide" or "crime*") AND ("hospital admission*" or "hospitalisation" or "hospitalization" or "emergency admission*" or "unscheduled admission*" or "unplanned admission*" or "emergency department" or "accident and emergency")<br>Language: English<br>Publication date: after 01 January 2000
Scopus (3458)	("multimorbidit*" or "multi-morbidit*" or "comorbidit*" or "co-morbidit*" or "polymorbidit*" or "poly-morbidit*" or "multicondition*" or "multi-condition*" or "multiple chronic condition*" or "morbidity burden*" or ((multiple or coexisting or co-existing or concurrent or con-current or comorbid* or co-morbid* or several*) adj2 (disease* or illness* or condition* or diagnosis* or morbid* or patholog*))) AND ("household*" or "coresiden*" or "co-residen*" or ((household or family or domestic or residen*) adj2 (composition* or member* or characterist* or dweller*)) or "neighborhood*" or "neighbourhood*" or "small area*" or "small-area*" or "place" or "communit*" or "census tracts" or "census block group" or "zip code*" or "post code*" or "electoral ward*" or "data zone*" or "output area*" or "lower super output area*" or "municipalit*" or "provinc*" or "socioeconomic" or "socio-economic" or "social capital" or "inequalit*" or "disparit*" or "income" or "wealth" or "financial problem*" or "financial difficult*" or "vocation*" or "training opportunit*" or "employment opportunit*" or "occupation* opportunit*" or "job opportunit*" or "job insecurit" or "education* opportunit*" or "education* achiev*" or "education* quality" or "fast-food" or "fast food" or "healthy food" or "social determinant" or "wider determinant" or "neighbourhood disorder" or "overcrowding" or "public housing" or "population density" or "urban" or "ghetto" or "rural" or "slum" or "estate" or "poverty area*" or "physical environment*" or "built environment*" or "living standard*" or "family structure" or "family breakdown" or "family disintegrat*" or "single parent*" or "housing tenure" or "Townsend" or "Carstairs" or "Index of Multiple Deprivation" or "Nam-Powers" or "Hollingshead Index" or "Breadline Britain Index" or "inverse care law" or "Singh ind*" or "Yost ind*" or "Kolak ind*" or "social determinants of health" or "neighbourhood archetype*" or "segregation indic*" or "ecological inversion" or "ecological fallacy" or "ecological bias" or "depriv*" or "impoverish*" or "disadvantage*" or "community cohesion" or "community network" or "community support" or "transport* quality" or "transport* services" or "transport link*" or "public transport*" or "travel time" or "community resource access*" or "walkability" or "public space" or "green space" or "open space" or "recreation*" or "leisure activit*" or "food outlet*" or "food environment" or "air pollution" or "particulate matter" or "nitrogen dioxide" or "sulphur dioxide" or "sulfur dioxide" or "carbon monoxide" or "crime*") AND ("hospital admission*" or "hospitalisation" or "hospitalization" or "emergency admission*" or "unscheduled admission*" or "unplanned admission*" or "emergency department" or "accident and emergency")<br>Publication date: after 01 January 2000
CINAHL (132)	("multimorbidit*" or "multi-morbidit*" or "comorbidit*" or "co-morbidit*" or "polymorbidit*" or "poly-morbidit*" or "multicondition*" or "multi-condition*" or "multiple chronic condition*" or "morbidity burden*" or ((multiple or coexisting or co-existing or concurrent or con-current or comorbid* or co-morbid* or several*) adj2 (disease* or illness* or condition* or diagnosis* or morbid* or patholog*))) AND ("household*" or "coresiden*" or "co-residen*" or ((household or family or domestic or residen*) adj2 (composition* or member* or characterist* or dweller*)) or "neighborhood*" or "neighbourhood*" or "small area*" or "small-area*" or "place" or "communit*" or "census tracts" or "census block group" or "zip code*" or "post code*" or "electoral ward*" or "data zone*" or "output area*" or "lower super output area*" or "municipalit*" or "provinc*" or "socioeconomic" or "socio-economic" or "social capital" or "inequalit*" or "disparit*" or "income" or "wealth" or "financial problem*" or "financial difficult*" or "vocation*" or "training opportunit*" or "employment opportunit*" or "occupation* opportunit*" or "job opportunit*" or "job insecurit" or "education* opportunit*" or "education* achiev*" or "education* quality" or "fast-food" or "fast food" or "healthy food" or "social determinant" or "wider determinant" or "neighbourhood disorder" or "overcrowding" or "public housing" or "population density" or "urban" or "ghetto" or "rural" or "slum" or "estate" or "poverty area*" or "physical environment*" or "built environment*" or "living standard*" or "family structure" or "family breakdown" or "family disintegrat*" or "single parent*" or "housing tenure" or "Townsend" or "Carstairs" or "Index of Multiple Deprivation" or "Nam-Powers" or "Hollingshead Index" or "Breadline Britain Index" or "inverse care law" or "Singh ind*" or "Yost ind*" or "Kolak ind*" or "social determinants of health" or "neighbourhood archetype*" or "segregation indic*" or "ecological inversion" or "ecological fallacy" or "ecological bias" or "depriv*" or "impoverish*" or "disadvantage*" or "community cohesion" or "community network" or

	"community support" or "transport* quality" or "transport* services" or "transport link*" or "public transport*" or "travel time" or "community resource access*" or "walkability" or "public space" or "green space" or "open space" or "recreation*" or "leisure activit*" or "food outlet*" or "food environment" or "air pollution" or "particulate matter" or "nitrogen dioxide" or "sulphur dioxide" or "sulfur dioxide" or "carbon monoxide" or "crime*") AND ("hospital admission*" or "hospitalisation" or "hospitalization" or "emergency admission*" or "unscheduled admission*" or "unplanned admission*" or "emergency department" or "accident and emergency")<br>Publication date: after 01 January 2000
Sociological Abstracts (623)	("multimorbidit*" or "multi-morbidit*" or "comorbidit*" or "co-morbidit*" or "polymorbidit*" or "poly-morbidit*" or "multicondition*" or "multi-condition*" or "multiple chronic condition*" or "morbidity burden*" or ((multiple or coexisting or co-existing or concurrent or con-current or comorbid* or co-morbid* or several*) adj2 (disease* or illness* or condition* or diagnosis* or morbid* or patholog*))) AND ("household*" or "coresiden*" or "co-residen*" or ((household or family or domestic or residen*) adj2 (composition* or member* or characterist* or dweller*)) or "neighborhood*" or "neighbourhood*" or "small area*" or "small-area*" or "place" or "communit*" or "census tracts" or "census block group" or "zip code*" or "post code*" or "electoral ward*" or "data zone*" or "output area*" or "lower super output area*" or "municipalit*" or "provinc*" or "socioeconomic" or "socio-economic" or "social capital" or "inequalit*" or "disparit*" or "income" or "wealth" or "financial problem*" or "financial difficult*" or "vocation*" or "training opportunit*" or "employment opportunit*" or "occupation* opportunit*" or "job opportunit*" or "job insecurit" or "education* opportunit*" or "education* achiev*" or "education* quality" or "fast-food" or "fast food" or "healthy food" or "social determinant" or "wider determinant" or "neighbourhood disorder" or "overcrowding" or "public housing" or "population density" or "urban" or "ghetto" or "rural" or "slum" or "estate" or "poverty area*" or "physical environment*" or "built environment*" or "living standard*" or "family structure" or "family breakdown" or "family disintegrat*" or "single parent*" or "housing tenure" or "Townsend" or "Carstairs" or "Index of Multiple Deprivation" or "Nam-Powers" or "Hollingshead Index" or "Breadline Britain Index" or "inverse care law" or "Singh ind*" or "Yost ind*" or "Kolak ind*" or "social determinants of health" or "neighbourhood archetype*" or "segregation indic*" or "ecological inversion" or "ecological fallacy" or "ecological bias" or "depriv*" or "impoverish*" or "disadvantage*" or "community cohesion" or "community network" or "community support" or "transport* quality" or "transport* services" or "transport link*" or "public transport*" or "travel time" or "community resource access*" or "walkability" or "public space" or "green space" or "open space" or "recreation*" or "leisure activit*" or "food outlet*" or "food environment" or "air pollution" or "particulate matter" or "nitrogen dioxide" or "sulphur dioxide" or "sulfur dioxide" or "carbon monoxide" or "crime*") AND ("hospital admission*" or "hospitalisation" or "hospitalization" or "emergency admission*" or "unscheduled admission*" or "unplanned admission*" or "emergency department" or "accident and emergency")<br>Publication date: after 01 January 2000
Cochrane (0)	("multimorbidit*" or "multi-morbidit*" or "comorbidit*" or "co-morbidit*" or "polymorbidit*" or "poly-morbidit*" or "multicondition*" or "multi-condition*" or "multiple chronic condition*" or "morbidity burden*" or ((multiple or coexisting or co-existing or concurrent or con-current or comorbid* or co-morbid* or several*) adj2 (disease* or illness* or condition* or diagnosis* or morbid* or patholog*)))
OpenGrey (0)	("multimorbidit*" or "multi-morbidit*" or "comorbidit*" or "co-morbidit*" or "polymorbidit*" or "poly-morbidit*" or "multicondition*" or "multi-condition*" or "multiple chronic condition*" or "morbidity burden*" or ((multiple or coexisting or co-existing or concurrent or con-current or comorbid* or co-morbid* or several*) adj2 (disease* or illness* or condition* or diagnosis* or morbid* or patholog*)))

Table S2. Study methodological quality ratings: Newcastle-Ottawa Assessment (NOAS), adapted for cross-sectional studies

Source	Selection				Comparability based on design and analysis	Outcome		Total
	Representativeness of the sample	Sample size	Non-respondents	Ascertainment of exposure		Assessment of outcome	Statistical test	
Chung et al (2016)	Half*	*	*	*	*	Half*	*	6 (Medium)
Lu et al (2017)	Half*	*	*	Half*	*	Half*	*	6.5 (Medium)
Fisher et al (2021)	Half*	*	*	Half*	**	*	*	7 (High)
Mbuya-Bienge et al (2021)	*	*	*	*	*	*	Half*	6.5 (Medium)
Oureta et al (2013)	*	*	Half*	*	*	*	Half*	6.5 (Medium)
Pati et al (20 15)	Half*	Half*	Half*	Half*	*	Half*	*	4.5(Medium)
Payne et al (2013)	*	*	*	*	**	*	*	8 (High)
Stafford et al (2020)	Half*	Half*	half*	*	*	*	*	5.5 (Medium)
Tomita et al (2021)	*	*	*	*	**	Half*	*	7.5 (High)
Wang et al	Half*	*	Half*	Half*	**	Half*	*	6 (Medium)

Methodological score: high = 7-8, medium = 4-6, low = 0-3

Box S1. Risk of bias Newcastle-Ottawa Assessment (NOAS), adapted for this study and application to examination of cross-sectional studies

A study can be awarded a maximum of one point for each numbered item within the Selection and Exposure categories. A maximum of two points can be given for the Comparability category.

Selection (maximum 4)

1. Representativeness of the sample

a. Truly representative of the average in the target population (random sample or whole population) *

b. Somewhat representative of the average in the target population (purposive sampling of representative healthcare sector areas or evidence that the sample is representative of the source population) half*

c. Selected group of users

d. No description of the sampling strategy

2. Sample size

a. Justified and satisfactory *

b. Adequately powered to detect a difference (at least 10 events per variable in multivariate analyses)*

c. Not justified

3. Non-respondents

a. Comparability between respondents and non-respondents characteristics is established, and the response rate is satisfactory (>60%)*

b. The response rate is unsatisfactory, or the comparability between respondents and non-respondents is unsatisfactory

c. No description of the response rate or the characteristics of the responders and non-responders

4. Risk factor exposure assessment

a. Assessment of exposure including type and duration from electronic health record or administrative database*

b. Assessment of exposure from questionnaire *

c. No description

Comparability (maximum 2)

5. The subjects in different outcome groups are comparable, based on the study design or analysis. Confounding factors are adjusted for

a. Study controls for multimorbidity and no multimorbidity, including age and gender adjustment**

b. Study controls for multimorbidity and no multimorbidity, without additional variable adjustment*

c. Study does not control for multimorbidity

Outcome (maximum 2)

6. Assessment of the outcome (emergency department or unscheduled hospital admission)

a. Electronic health record or health payment record account of healthcare use *

b. Reported healthcare use from questionnaire or no described data source half*

7. Statistical test

a. The statistical test used to analyse the data is clearly described and appropriate, and the measurement of the association is presented as either an OR, CI and P value or a beta coefficient, SE, and P value*

b. The statistical test is not appropriate, not described, or incomplete
