# Supplementary material for: Household and area determinants of emergency department attendance and hospitalisation in people with multimorbidity: a systematic review
Source: BMJ Open. 2022 Oct 3;12(10):e063441. doi: 10.1136/bmjopen-2022-063441 (PMC9535173; doi:10.1136/bmjopen-2022-063441)
Supplement: Author's manuscript [file bmjopen-2022-063441.draft_revisions.pdf]

BMJ Open is committed to open peer review. As part of this commitment we make the peer review history of every article we publish publicly available.

When an article is published we post the peer reviewers' comments and the authors' responses online. We also post the versions of the paper that were used during peer review. These are the versions that the peer review comments apply to.

The versions of the paper that follow are the versions that were submitted during the peer review process. They are not the versions of record or the final published versions. They should not be cited or distributed as the published version of this manuscript.

BMJ Open is an open access journal and the full, final, typeset and author-corrected version of record of the manuscript is available on our site with no access controls, subscription charges or pay-per-view fees (http://bmjopen.bmj.com).

If you have any questions on BMJ Open's open peer review process please email info.bmjopen@bmj.com

BMJ Open

Household and area determinants of emergency department attendance and hospitalisation in people with multimorbidity: a systematic review

Journal:	*BMJ Open*
Manuscript ID	bmjopen-2022-063441
Article Type:	Original research
Date Submitted by the Author:	01-Apr-2022
Complete List of Authors:	MacRae, Clare; The University of Edinburgh<br>Fisken, Harry; The University of Edinburgh Edinburgh Medical School<br>Lawrence, Edward; Ninewells Hospital and Medical School<br>Connor, Thomas; The University of Edinburgh Edinburgh Medical School<br>Pearce, Jamie; University of Edinburgh Institute of Geography, Institute of Geography<br>Marshall, Alan; The University of Edinburgh Social Policy, Social Policy<br>Lawson, Andrew; Medical University of South Carolina, Department of Public Health Sciences<br>Dibben, Chris; University of Edinburgh Institute of Geography<br>Mercer, Stewart; The University of Edinburgh, The Usher Institute of Population Health Sciences and Informatics<br>Guthrie, Bruce; University of Edinburgh College of Medicine and Veterinary Medicine
Keywords:	EPIDEMIOLOGY, PUBLIC HEALTH, STATISTICS & RESEARCH METHODS

SCHOLARONE™
Manuscripts

I, the Submitting Author has the right to grant and does grant on behalf of all authors of the Work (as defined in the below author licence), an exclusive licence and/or a non-exclusive licence for contributions from authors who are: i) UK Crown employees; ii) where BMJ has agreed a CC-BY licence shall apply, and/or iii) in accordance with the terms applicable for US Federal Government officers or employees acting as part of their official duties; on a worldwide, perpetual, irrevocable, royalty-free basis to BMJ Publishing Group Ltd ("BMJ") its licensees and where the relevant Journal is co-owned by BMJ to the co-owners of the Journal, to publish the Work in this journal and any other BMJ products and to exploit all rights, as set out in our licence.

The Submitting Author accepts and understands that any supply made under these terms is made by BMJ to the Submitting Author unless you are acting as an employee on behalf of your employer or a postgraduate student of an affiliated institution which is paying any applicable article publishing charge ("APC") for Open Access articles. Where the Submitting Author wishes to make the Work available on an Open Access basis (and intends to pay the relevant APC), the terms of reuse of such Open Access shall be governed by a Creative Commons licence – details of these licences and which Creative Commons licence will apply to this Work are set out in our licence referred to above.

Other than as permitted in any relevant BMJ Author's Self Archiving Policies, I confirm this Work has not been accepted for publication elsewhere, is not being considered for publication elsewhere and does not duplicate material already published. I confirm all authors consent to publication of this Work and authorise the granting of this licence.

For peer review only

Household and area determinants of emergency department attendance and hospitalisation in people with multimorbidity: a systematic review

Authors: Clare MacRae[1], Harry Fisken[2], Edward Lawrence[3], Thomas Connor[2], Jamie Pearce[4], Alan Marshall[5], Andrew Lawson[1, 6], Chris Dibben[4], Stewart W Mercer[1], Bruce Guthrie[1]

[1]Usher Institute, College of Medicine and Veterinary Medicine, University of Edinburgh, EH8 9AG

[2]The University of Edinburgh Medical School, College of Medicine and Veterinary Medicine, University of Edinburgh, EH8 9AG

[3]Ninewells Hospital and Medical School, Dundee, DD1 9SY

[4]School of Geosciences, College of Science and Engineering, University of Edinburgh, EH9 3JW

[5]School of Social and Political Science, University of Edinburgh, Chrystal Macmillan Building, Edinburgh, EH8 9LD

[6]Department of Public Health Sciences, Medical University of South Carolina, 135 Cannon Street, Charleston, South Carolina, USA

Corresponding author: Dr Clare MacRae, Usher Institute, College of Medicine and Veterinary Medicine, University of Edinburgh, EH8 9AG, clare.macrae@ed.ac.uk

PROSPERO registration number: CRD42021283515

For peer review only

For peer review only - http://bmjopen.bmj.com/site/about/guidelines.xhtml

Abstract

Objectives: Multimorbidity is one of the biggest challenges facing healthcare internationally. Emergency department (ED) attendance and hospitalisation rates are higher in people with multimorbidity, but most research focuses on associations with individual characteristics, ignoring household or community mediators of service use. We systematically review studies examining associations between household or area context and hospital attendance in people with multimorbidity.

Methods: Twelve electronic databases were searched for full text articles published between January 1st 2000, and September 21st 2021. Eligible studies included adults aged ≥16 years with multimorbidity, exposure(s) were household and/or area determinants of health, and outcomes were ED attendance and/or hospitalisation. Given high study heterogeneity, narrative synthesis was performed. PROSPERO registration number CRD42021283515.

Results: After de-duplication, 10,721 titles and abstracts were screened, and 142 full-text articles reviewed, of which nine were eligible for inclusion. In people with multimorbidity, household food insecurity was associated with hospitalisation (OR 1.58, 95%CI1.06-2.36 in concordant, and OR 1.54, 95%CI1.04-2.28 in discordant multimorbidity). People with multimorbidity living in the most versus least deprived areas attended ED more frequently (8.9%, 95%CI 8.6-9.1 in most-deprived versus 6.3% 95%CI6.1-6.6 in least-deprived), had higher rates (26% in most versus 22% in least), and were more frequently admitted (probability 6.4%, 95%CI 5.8-7.2 in most versus 4.2% 95%CI 3.8-4.7 in least). There was non-conclusive evidence that household income is associated with ED attendance and hospital admission. No statistically significant relationships were found between marital status, living with others with multimorbidity, or living in urban versus rural areas and ED attendance or hospitalisation.

Conclusions: There is some evidence that household and area context mediate associations of multimorbidity with ED attendance and hospitalisation, but firm conclusions are constrained by the small number of studies published and study design heterogeneity. Further research is required on large population samples using robust analytical methods.

Word count: 298

For peer review only - http://bmjopen.bmj.com/site/about/guidelines.xhtml

Strengths and limitations

- Comprehensive literature search of twelve electronic databases to examine associations between household or area context and hospital attendance, specifically in people with multimorbidity
- Reporting as per Preferred Reporting Items for Systematic Reviews and Meta-Analyses and Synthesis Without Meta-analysis guidelines
- Limited evidence available in current literature with a small number of studies meeting eligibility criteria
- Heterogeneity in multimorbidity definition and measurement, and study exposures, outcomes, and analysis methods, precluded meta-analysis

For peer review only

For peer review only - http://bmjopen.bmj.com/site/about/guidelines.xhtml

Introduction

Multimorbidity is usually defined as the presence of two or more long-term physical or mental health conditions, and is one of the biggest challenges facing healthcare internationally. Multimorbidity is becoming more common globally because of population ageing,[1, 2] improved survival after acute illnesses such as myocardial infarction,[3] and changing patterns of mental health in younger people.[4] There are large independent associations between multimorbidity and adverse outcomes including frailty,[5] reduced functional health status,[6] and hospital attendance.[7] People with multimorbidity are at nearly three times the risk of hospital admission compared to those without.[7]

Research usually compares different groups of people with multimorbidity, for example defined by their individual characteristics, such as age, sex, and lifestyle factors.[8, 9] However, for this important patient group, social, and environmental contexts are rarely examined in depth. People live in a social context, where attitudes and practices can be influenced by those around them, for example households have been described as "a living and developing unit of interdependent members, sharing common internal and external conditions and interactions".[10] Research examining general adult populations (not stratified by multimorbidity) has found that people who live in a household with fewer financial or food resources,[11, 12] and single person households,[13] are at higher risk of ED attendance and hospitalisation. Additionally, the areas in which people live can influence health. For example populations not stratified by multimorbidity living in urban areas, and in areas with low socioeconomic status (SES), with associated additional health burdens, are similarly at risk.[14] Households and areas are therefore an important unit of measurement when considering patterns of disease and healthcare use.[15] To date, there is a paucity of synthesis of studies examining these data in people with multimorbidity.

Understanding whether and how context influences outcomes is important to ensure that the call by the Academy of Medical Sciences for research into "how to organise healthcare systems to better manage patients with multimorbidity" is based on appropriate understanding of both the individual and their social and environmental context.[2] The aim of this review is to examine, in people with multimorbidity, associations of household and area contextual exposures with emergency department (ED) attendance and hospitalisation.

Methods

Method development and reporting of findings of the systematic review were based on the PRISMA-P 2020 checklist.[16] The protocol was registered with PROSPERO CRD42021283515.

For peer review only - http://bmjopen.bmj.com/site/about/guidelines.xhtml

Twelve electronic databases (MEDLINE/OVID, Embase, Global Health, PsycINFO, ASSIA, CAB Abstracts, Science Citation Index Expanded/ISI Web of Science, Scopus, Cumulative Index to Nursing and Allied Health Literature [CINAHL], Sociological Abstracts, and the Cochrane Library), OpenGrey, and reference lists, were searched for full text articles meeting eligibility criteria, published between 1st January 2000, and 21st September 2021. An empirical approach to deriving the search terms was followed using a test set of three critical papers, one examining the household and area determinants of multimorbidity,[17] a second examining the definition and operationalisation of the term multimorbidity,[18] and a third meeting the study inclusion and exclusion criteria.[19] Key search terms, derived through reference to medical subject heading terms in test papers, iterated within MedLine to maximise sensitivity and specificity for relevant articles, were divided into three sets pertaining to multimorbidity, contextual exposures, and healthcare use (Table S1).

Eligible studies included populations of adults aged 16 years and over, from the general population, who had been assessed for, and had, the presence of multimorbidity or multiple long-term conditions (LTCs), defined according to the Academy of Medical Sciences core definition of multimorbidity as 'two or more chronic conditions' (Table 1).[2] Study exposure(s) examined were one or more household and/or area determinant of health, including built environment, and socioeconomic determinants of health (Table S1).[20] Study outcomes were ED attendance and/or hospitalisation.

For peer review only - http://bmjopen.bmj.com/site/about/guidelines.xhtml

Table 1. Study inclusion and exclusion criteria

	Inclusion	Exclusion
**Population**	Adult participants from the general population residing in the community, assessed for the presence of multiple LTCs (multimorbidity) aged 16 years and older	Participants initially selected based on the presence of index diseases, including any study examining comorbidity<br>Studies exclusively examining children aged 15 years and younger<br>Participants within hospital settings, or examination of readmission where the dominator is previously hospitalised participants
**Exposure**	Study exposure(s): ≥ 1 household or area-level socioeconomic determinant of health (SDoH) in alignment with the World Health Organisation (WHO) Commission on SDoH (CSDH) Framework[20]<br>Household: One residential unit, characteristics can include reference to the built environment, or the occupants living within<br>Area: Geographical area within which a person lives<br>All area sizes larger than household unit	Study exposure(s) include individual SDoH only (e.g., ethnicity). Study exposure(s) are direct "causes" of ill-health, such as health behaviours (e.g., smoking)
**Comparator**	Study reports comparator group(s) for SDoH exposures (e.g., prevalence of hospitalisation admission in lowest versus highest household size)	Study does not report a comparator group for SDoH exposure(s)
**Outcome**	Prevalence or incidence studies examining emergency department use and hospitalisation	Studies not examining emergency department use or hospitalisation
**Study design**	Peer-reviewed studies of quantitative research designs (cross-sectional and longitudinal)	Systematic reviews, meta-analyses, clinical trials, and qualitative research

For peer review only - http://bmjopen.bmj.com/site/about/guidelines.xhtml

Following removal of duplicate records, independent double screening for relevance was performed for all titles and abstracts, using Covidence software,[21] and relevant full-text studies were selected (Figure 1). Conflicts were resolved through discussion between screeners (CM, HF, EL, TC) at each stage of screening and eligibility of all included studies was verified by a fifth author (BG).

The Newcastle-Ottowa Quality Assessment Scale (NOAS) tool[22] was adapted (Box S1), and study quality screening for each paper was performed independently by two of the researchers (CM plus one of HF, EL, TC), with consensus achieved through discussion.

CM performed data extraction using a custom spreadsheet to record study design, location, population (age, definition of multimorbidity), exposures (household and/or area), and outcome measures (ED attendance and/or hospitalisation). If data were missing or unclear, we contacted corresponding authors for clarification who provided additional data, and information regarding study methodology and analyses.

Study methodologies, exposures, outcomes, and effect measures were diverse and heterogenous, and narrative synthesis was therefore performed according to Synthesis Without Meta-analysis (SWiM)[23] and PRISMA guidelines.[16] Studies were grouped according to exposure examined, to ensure meaningful presentation of reviewed evidence.[24] Presentation of results using a standardised metric was not possible given the heterogeneity of outcome measures. Due to the small number of included studies, all study results were reported with quantitative figures given for statistically significant findings. Narrative comparison of heterogeneity between studies examining each exposure was performed by reviewing results within each group, comparing methodologies, exposures, and outcomes. Patients or the public were not involved in the design, conduct, reporting, or dissemination plans of this research.

Patient and Public Involvement

Patients or the public were not involved in the design, conduct, reporting, or dissemination plans of this research.

Results

The systematic search identified 16,853 articles. After removal of 6,132 duplicates, 10,721 titles and abstracts were screened, of which 10,579 were excluded at the first screening stage. Full text-screening of 142 articles identified nine studies meeting eligibility criteria. Screening of reference lists yielded no additional studies eligible for inclusion (Figure 1).

For peer review only - http://bmjopen.bmj.com/site/about/guidelines.xhtml

Two studies were conducted in North America,[25, 26] two in Asia,[27, 28] three in Europe,[19, 29, 30] one in Africa,[31] and one study used multiple sites[32] (Table 2). All studies used cross-sectional data; two used data derived from existing cohort studies.[25, 26] Study sample size ranged from 1,670 to 5,316,830 (median 28,361 participants, interquartile range [IQR] 9,222-108,815). Six studies reporting regression model outcomes,[19, 25, 27, 28, 31] used smaller populations (1,670-162,464 participants, median 23,505, IQR 9,222-33,054), than the two studies reporting rates of hospital attendance (2,262,698-5,316,830, median 3,789,764, IQR 3,026,231-4,553,297).[26, 29] Each included study examined a specific age-group, which were 15-65 years old (one study),[27] ≥18 years old (two studies),[26, 28] ≥20 years old (two studies),[30, 32] ≥40 years old (one study),[31] ≥50 years old (one study),[19] and ≥65 years old (two studies).[25, 29]

For peer review only

Table 2. Study characteristics.

Author	Study purpose	Study design	Data source a	Sample size	Setting and date	Target population	Multimorbidity definition	Household exposure	Area exposure	Primary outcome measure(s)
Fisher et al (2021),[25] Canada	Examine socio-demographic and health factors in multimorbidity and one-year acute care service use	Cross-section from cohort study	Health administrative data: Canadian Community Health Survey	28,361	General population; multiple cycles 2005–2006, 2007–2008, 2009–2010, 2011–2012	65 - 85 years old	≥ 2 LTCSs out of 12 LTCs (identifiable in administrative data,prevalent in older adults, and frequently reported in literature), physical and mental LTCs	Household income	NA	12-month ED attendance and hospitalisation. Dichotomous: attendance or no attendance
Lu et al (2017),[27] China	Examine inequalities limit the utilisation of health services among the China labour force	Cross-sectional study	China Labour Force Dynamic Survey	23,505	Adult population in employment; 2014	15-65 years old	≥ 2 LTCSs out of 10 LTCs, no description of rationale for disease inclusion, physical LTCs only	Marital status	NA	12-month hospitalisation rate (no differentiation planned/unplanned)
Mbuya-Bienge et al (2021),[26] Canada	Explore socioeconomic position and frequency of use of healthcare in multimorbidity	Cross-section from cohort study	Quebec Integrated Chronic Disease System	5,316,830	General population; 2011-2012	≥18 years old	≥ 2 LTCSs out of 31 LTCs (amalgamation of 17 Charlson and 30 Elixhauser LTC lists), physical and mental LTCs	NA	Area SES	12-month ED attendance rate
Orueta et al (2013),[29] Spain	Explore prevalence and costs of multimorbidity by socioeconomic levels	Cross-sectional study	Primary care electronic health records, hospital admission care databases	2,262,698	General population: population, cross-section date 31 August 2011	≥65 years old	≥ 2 LTCSs out of 52 LTCs, adapted Barnett et al LTC list,[41] physical and mental LTCs	NA	Area SES	12-month hospitalisation rate (no differentiation planned/unplanned)
Pati et al (20 15),[28] India	Examine relationship between multimorbidity and health care utilisation	Cross-sectional study	Multi-stage random sampling approach to interviews of adult patients in primary care facilities.	1,670	General population, 40 primary care facilities: 20 publicly and 20 privately funded; October 2013-March 2014	≥18 years old	≥ 2 LTCSs out of 22 LTCs most prevalent in primary care based on literature search and chart review, physical and mental LTCs	Marital status	Residential location: rural, urban	12-month hospital in-patient or out-patient hospital use (no differentiation planned/unplanned)
Payne et al (2013) [30]	Examine association between unplanned hospital admission and socioeconomic deprivation in multimorbidity	Cross-sectional study	Scottish Practice Team Information dataset	180,815	General population registered with General Practitioners in Scotland in 2006	≥18 years old	≥ 2 LTCSs out of 52 LTCs as Barnett et al LTC list,[41] physical and mental LTCs	NA	Area SES	12-month urgent or emergency hospital admission or potentially preventable urgent or emergency hospital admission

For peer review only

For peer review only - http://bmjopen.bmj.com/site/about/guidelines.xhtml

Stafford et al (2020),[19] England	Test whether co-residence with a person with multiple LTCs is associated with healthcare utilisation	Cross-sectional study	Barking and Dagenham (B&D) local government and health data, and Clinical Practice Research Datalink (CPRD)	9,222 (B&D) and 10,528 (CPRD)	General population; 2016-2018	≥ 50 years old living in 2-person households	≥ 2 LTCSs out of 16 LTCs, no description of rationale for disease inclusion, physical and mental LTCs	Health status of other household members (with or without multiple LTCs)	NA	Non-elective hospital admissions and ED attendance Dichotomous: hospital use or no hospital use
Tomita et al (2021),[31] Tanzania	Examine association between multimorbidity and inpatient hospitalisation	Cross-sectional study	Dar es Salaam Health Demographic Surveillance System; computer-assisted personal interviewing system	2,299	General population; 2017-2018	≥ 40 years old	≥ 2 LTCSs out of 8 LTCs (concordant: 1 domain of mental health/non-communicable/communicable health, discordant: ≥ 2 domains), physical and mental LTCs	Marital status<br><br>Household food insecurity	NA	12-month hospitalisation rate (no differentiation planned/unplanned)
Wang et at (2015),[32] Scotland, China, Hong Kong	Examine relationships of multimorbidity and household income with hospital admission in three countries.	Cross-sectional study	Scotland: Scottish Health Survey<br>China: Routine administrative data from three prefectures<br>Hong Kong: Thematic Household Survey	36,921<br><br>162,464<br><br>29,187	General population; 2008 to 2011<br><br>General population; 2011<br><br>General population; 2011	≥ 40 years old	≥ 2 LTCSs out of 31 LTCs from 14 ICD-10 categories. Selected if they occurred commonly in the three country-specific datasets, physical and mental LTCs	Household income	NA	Admission to hospital (Scotland), admission to hospital with no versus with medical insurance China), admission to hospital and admission to public and private hospital (Hong Kong)

For peer review only - http://bmjopen.bmj.com/site/about/guidelines.xhtml

Multimorbidity was defined as the presence of two or more LTCs by all studies, with the number of LTCs included in multimorbidity measures ranging from 8 to 52 (median 22, IQR 12-31). Eight studies included both physical and mental LTCs,[19, 25, 26, 28-33] and one study only included physical LTCs.[27] Various approaches were taken to defining the list of LTCs included, ranging from no included rationale by Lu et al[27] and Stafford et al,[19] adaptation of previous studies examining multimorbidity by Oureta et al,[29] to including LTCs included in the Charlson and Elixhauser indexes by Mbuya-Bienge et al.[26] Tomita et al[31] examined both concordant and discordant multimorbidity, fitting separate logistic regression models for each. In this study, discordant multimorbidity was defined as the occurrence of LTCs in ≥ two domains in mental health, non-communicable, and communicable disease.

The nine included studies examined six unique exposures, where an exposure was defined as any household or area characteristic associated with ED attendance or hospitalisation. Six studies examined household,[19, 25, 27, 28, 31, 32] and four studies examined area exposures.[26, 28-30] One study, by Pati et al,[28] examined both household and area exposures.

Household exposures examined were household income (two studies),[25, 32] multimorbidity status of other household members (one study),[19] household food insecurity (one study),[31] and marital status (three studies).[27, 28, 31] Data on household income were derived through household surveys in both included studies.[25, 32] Household food insecurity was defined using a cross-cultural measure, where survey participants were asked how often there was no food in their household, and how often household members went to sleep hungry or did not eat for a day.[31] Multimorbidity status of other household members was determined through linkage of local government and health provider administrative data with the unique property reference number,[19] a unique identifier for every addressable location in the United Kingdom.[34] Marital status was the most examined household exposure, but studies varied in how they examined this. Pati et al[28] classified participants as currently married and currently not married, and Lu et al[27] defined groups as married and single/divorced. Tomita et al[31] divided participants into three groups; currently married, never married, and separated/divorced/widowed. Stafford et al[19] examined multimorbidity status of household the other household resident in two-person households, with no reference to relationship status.

Area exposures examined were area SEP (three studies),[26, 29, 30] and rurality (one study).[28] All studies examining area SEP used a similar approach to deriving their SEP measure, using quintiles of a score derived from census tract deprivation indexes, based on rates of unemployment, educational attainment, and type of employment. Rurality was examined as a dichotomous exposure of rural versus urban, derived from population size and density, and proportion of workers.

For peer review only - http://bmjopen.bmj.com/site/about/guidelines.xhtml

Reported outcomes were any ED attendance (two studies),[25, 26] any hospitalisation (whether planned or unplanned) (five studies),[25, 27, 29, 31, 32] any inpatient or outpatient hospital attendance (one study),[28] any ED attendance or unplanned hospitalisation (one study),[19] and any unplanned or emergency hospital admission or unplanned or emergency potentially avoidable hospital admission (one study).[30] No studies presented results stratified by the number of attendances in the study period.

Methodological quality of studies varied across studies and was rated high in three studies,[25, 30, 31] and medium in six[19, 26-29, 32] (Table S2). Potential bias related to reporting of hospital attendance outcomes, where studies used either questionnaire based self-reported outcomes,[27, 28, 31, 32] or electronic health records and health payment systems,[19, 25, 26, 29] therefore differences in accuracy and reporting could exist between these studies. Use of statistical reporting varied between studies, with six studies performing regression models with different model outcomes, including odds ratios (ORs),[19, 25, 27, 31] incidence rate ratios (IRRs),[28] event probability,[32] as well as studies examining unadjusted rates of hospital attendance.[26, 29]

Fisher et al,[25] a Canadian study rated as high quality, examined associations between household income and service use in people with multimorbidity, with separate regression models fitted to sub-groups defined by age-group (65-74 and 75-84 years), sex, multimorbidity (categorised as 2-3 and ≥4 LTCs), and outcomes (ED attendance and hospitalisation) (Table 3). In people with multimorbidity, ORs for ED attendance in people with middle (Canadian \$30-\$79.9K) and lower (<\$30K) household income, versus high (>\$80K), were almost all >1 (15 out of 16). However, 12 (out of 16) had wide confidence intervals crossing 1, which is likely to reflect small numbers in each strata. There was some evidence of a dose-response relationship (ORs for low versus high income were always larger than ORs for medium versus high income), and estimated ORs were all small to moderate in size (maximum likelihood in men aged 65-74 with ≥4 LTCs was OR 2.74, 95C%I 1.12-6.66 for low versus high household income). In models with hospital admission as outcome, the ORs for middle and lower household income, versus high, were all >1, however only two (out of 16) ORs had 95%CIs not crossing 1, and a similar dose-response relationship between degree of income and hospital admission was seen.

For peer review only - http://bmjopen.bmj.com/site/about/guidelines.xhtml

Table 3. Household exposure study results

Study exposure	Study	Multimorbidity measurement	Exposure definition	Outcome	Result metric	Key Results
Household income	Fisher et al[25]	≥2 LTCs	Household income	ED use	ORs and 95%CIs	Statistically insignificant relationship between lower household income and increased likelihood of ED attendance in people with multimorbidity<br>Women 75-84 years old with 2-3 LTCs ($30k-$79.9k versus >$80k OR 2.05, 95%CI 1.09-3.86; <$30k versus >$80k OR 2.49, 95%CI 1.33-4.64, p=0.29)<br>Men 65-74 years old with 2-3 LTCs ($30k-$79.9k versus >$80k OR 1.58, 95%CI 1.05-2.37, p=0.22)<br>Men 65-74 years with ≥4 LTCs (<$30k versus >$80k OR 2.74, 95%CI 1.12-6.6, p=0.22)
				Any hospital use		Statistically insignificant relationship between lower household income and increased likelihood of any hospital use in people with multimorbidity<br>Women 75-84 years old with 2-3 LTCs (for $30 k-$79.9 k versus >$80 k OR 1.65, 95% CI1.08-2.51, p=0.20)
	Wang et al[32]	≥2 LTCs	Household income	Admission to hospital (Scotland and China), admission to public and private hospital (Hong Kong)	Predicted probability (P) and 95%CIs	Higher probability of hospital admission in Scotland and public hospital admission in Hong Kong, and lower probability of hospital admission in China and private hospital admission in Hong Kong, with lower household income<br><br>Scotland:<br>Two LTCs: lowest household income associated with higher probability (18.7%, 95%CI 18.3-19.1) of hospital admission than highest (11.1%, 95%CI 10.7-11.4)<br>Three LTCs: lowest household income (25.5%, 95% CI24.8-26.2) versus highest (16.0%, 95%CI 15.2-16.8)<br>Four LTCs: lowest household income (30.7%, 95%CI 30.0-31.4) versus highest (20.2%, 95%CI 18.6-21.6)<br><br>China (no medical insurance):<br>Two LTCs: lowest household income associated with lower probability (8.2%, 95%CI 7.9-8.5) of hospital admission than highest (13.1%, 95%CI 12.2-14.0)<br>Three LTCs: lowest household income (12.2%, 95%CI 11.4-13.0) versus highest (19.3%, 95%CI 17.0-21.6)<br>Four LTCs: lowest household income (24.7%, 95%CI 23.3-26.1) versus highest (36.1%, 95%CI 33.6-38.6)<br><br>Hong Kong (public hospital):<br>Two LTCs: lowest household income associated with higher probability (16.8%, 95%CI 16.45-17.15) of hospital admission than highest (7.7%, 95%CI 7.3-8.1)<br>Three LTCs: lowest household (23.4%, 95%CI 22.3-24.5) versus highest (12.1%, 95%CI 11.3-12.9)<br>Four LTCs: lowest household income (30.4%, 95%CI 29.8-31.0) versus highest (16.8%, 95%CI 15.4-18.2)
Multimorbidity status of the other household resident	Stafford et al[19]	≥ 2 LTCs	Multimorbidity status of the other household resident	ED use	ORs and 95%CIs	No difference in ED attendance between people where the other household resident had multimorbidity (OR 1.08, 95%CI 0.95-1.23) in people with multimorbidity
Household food insecurity	Tomita et al[31]	Concordant multimorbidity*	Little-to-no versus severe food insecurity	Hospitalisation	ORs and 95%CIs	Severe household hunger associated with increased likelihood of hospitalisation (OR 1.58, 95%CI 1.06-2.36) in people with concordant multimorbidity
		Discordant multimorbidity*	Little-to-no versus severe food insecurity	Hospitalisation	ORs and 95%CIs	Severe household hunger associated with increased likelihood of hospitalisation (OR 1.54, 95%CI 1.04-2.28) in people with discordant multimorbidity

For peer review only - http://bmjopen.bmj.com/site/about/guidelines.xhtml

Household marital status	Lu et al[27]	≥ 2 LTCs	Married versus single/divorced	Hospitalisation	ORs and 95%CIs	No difference in hospitalisation between married or single/divorced (OR 1.04, 95%CI 0.54-2.02) in people with multimorbidity
	Pati et al[28]	≥ 2 LTCs	Currently married versus currently not married	Inpatient and outpatient hospital use	IRRs and 95%CIs	No difference in inpatient or outpatient hospital use between currently married versus currently not married (IRR 1.17, 95%CI 0.85-1.61) in people with multimorbidity
	Tomita et al[31]	Concordant multimorbidity[*]	Currently married versus never married or separated/divorced	Hospitalisation	ORs and 95%CIs	No difference in hospitalisation between currently married versus never married (OR 1.43, 95%CI 0.62-3.28), or separated/divorced (OR 1.24, 95%CI 0.86-1.78), in people with multimorbidity
		Discordant multimorbidity[*]	Currently married versus never married or separated/divorced	Hospitalisation	ORs and 95% CIs	No difference in hospitalisation between currently married versus never married (OR 1.44, 95% CI 0.63-3.28) or separated/divorced (OR 1.27, 95% CI 0.83-1.85) in people with multimorbidity

[*] See Table 2 for definition of concordant and discordant multimorbidity

For peer review only - http://bmjopen.bmj.com/site/about/guidelines.xhtml

Wang et al,[32] a study rated as medium quality, found that in people with multimorbidity, lower household income was associated with a higher probability of hospital admission in Scotland, and public hospital admission in Hong Kong. A dose-response relationship was seen where probability of admission rose as the number of LTCs increased, for example, probability for hospitalisation was higher in people with ≥4 LTCs (probability 30.7%, 95%CI 30.3-31.7) than people with 2 LTCs (18.85%, 95%CI 18.4-19.2) in the lowest household income group in Scotland. However, a reversed relationship, lower household income associated with lower probability of hospital admission, was seen in China and in private hospitals in Hong Kong. For example, people with lowest household income had lowest probability of hospital admission (24.7%, 95%CI 23.4-26.1) versus people with highest household income (36.1%, 95%CI 33.6-38.6), in people with ≥4 LTCs with no medical insurance in China, and the pattern was the same regardless of medical insurance status.

An English study by Stafford et al,[19] examined the multimorbidity status of the other household resident in two-person households. In this study rated as medium quality, no significant difference between living with someone who had multimorbidity, versus someone who did not have multimorbidity, was found in association with ED attendance (OR 1.08, 95%CI 0.95-1.23).

A statistically significant relationship between severe household hunger and hospitalisation was found in a Tanzanian study by Tomita et al,[31] rated as high quality. This relationship was found in both concordant (severe versus none-to-moderate household hunger OR 1.58, 95%CI 1.06-2.36) and discordant (severe versus none-to-moderate household hunger OR 1.54, 95%CI 1.04-2.28) multimorbidity.

No significant association between marital status and any outcome was found in the three studies examining this. Lu et al,[27] a Chinese study rated as medium quality, found a small but not statistically significant relationship between being married and hospitalisation (married versus unmarried OR 1.04, 95%CI 0.54-2.02). Pati et al,[28] a study from India rated as medium quality, found a larger but not statistically significant relationship in the opposite direction between marital status and any inpatient or outpatient hospital attendance (unmarried versus married incidence rate ratio [IRR] 1.17, 95%CI 0.85-1.61). Similarly, Tomita et al[31] found a small but not statistically significant association between not being married and hospitalisation in people with concordant multimorbidity (never married versus married OR 1.43, 95%CI 0.62-3.28; divorced/separated vs married OR 1.24, 95%CI 0.86-1.78) and in people with discordant multimorbidity (never married versus married OR 1.44, 95%CI 0.63-3.28; divorced/separated versus married OR 1.27, 95%CI 0.83-1.85) in Tanzania.

More people living in the most versus least deprived quintile of areas attended ED in a medium quality Canadian study by Mbuya-Bienge et al[26] (Table 4). Results were stratified by number of LTCs: for people

For peer review only - http://bmjopen.bmj.com/site/about/guidelines.xhtml

with two LTCs 8.9% (95%CI 8.6-9.1) living in most deprived areas attended ED versus 6.3% (95%CI 6.1-6.6) in the least deprived areas; for people with three LTCs 12.0% (95%CI 11.6-12.5) versus 9.5% (95%CI 9.1-10.0); for people with ≥ four LTCs 18.3% (95%CI 17.9-18.8) versus 16.8% (95%CI 16.3-17.4). Likewise, more people living in the most versus least deprived area quintiles were hospitalised in the Spanish study by Orueta et al,[29] rated as medium quality. This study also stratified the number of LTCs and found that in people with four to six LTCs, 26% living in most deprived areas were hospitalised versus 22% of people in the least deprived areas; for people with seven to nine LTCs, 48% versus 44% (no 95%CIs or cohort size from which to derive these). However, in people with ≥10 LTCs, rates of hospitalisation were lower in people living in the most, 68%, versus least, 70%, deprived areas. Neither study examining the effect of SEP performed regression analyses.

For peer review only

For peer review only - http://bmjopen.bmj.com/site/about/guidelines.xhtml

Table 4. Area exposure study results

Study exposure	Study	Multimorbidity measurement	Exposure definition	Outcome	Result metric	Key Results
Area SEP	Mbuya-Bienge et al[26]	2 LTCs	Most versus least deprived area quintile	ED use	Prevalence % and 95%CIs	Lower rates of ED use in most (8.9%, 95%CI 8.6-9.1) versus least (6.3%, 6.1-6.6) deprived area quintile in people with multimorbidity
		3 LTCs	Most versus least deprived area quintile	ED use	Prevalence % and 95%CIs	Lower rates of ED use in most (12.0%, 11.6-12.5) versus least (9.5%, 95%CI 9.1-10.0) deprived area quintile in people with multimorbidity
		≥ 4 LTCs	Most versus least deprived area quintile	ED use	Prevalence % and 95%CIs	Lower rates of ED use in most (18.3%, 95%CI 17.9-18.8) versus least (16.8%, 95%CI 16.3-17.4) deprived area quintile in people with multimorbidity
	Orueta et al[29]	4-6 LTCs	Most versus least deprived area quintile	Hospitalisation	Prevalence % (no 95%CIs)	Lower rates of hospitalisation in most (26%) versus least (22%) deprived area quintile in people with multimorbidity
		7-9 LTCs	Most versus least deprived area quintile	Hospitalisation	Prevalence % (no 95%CIs)	Lower rates of hospitalisation in most (48%) versus least (44%) deprived area quintile in people with multimorbidity
		≥ 10 LTCs	Most versus least deprived area quintile	Hospitalisation	Prevalence % (no 95%CIs)	Higher rates of hospitalisation in most (70%) versus least (86%) deprived area quintile in people with multimorbidity
	Payne et al[30]	2 LTCs	Most versus least deprived area quintile	Hospitalisation	Predicted probability and 95%CIs	Higher probability of unplanned hospital admission in men with physical only multimorbidity in most (6.4%, 95%CI 5.8-7.2) versus least (4.2%, 95%CI 3.8-4.7)<br><br>Higher probability of unplanned hospital admission in men with physical and mental multimorbidity in most (12.1%, 95%CI 11.0-13.6) versus least (8.1%, 95%CI 7.3-9.2)
		3 LTCs	Most versus least deprived area quintile	Hospitalisation	Predicted probability and 95%CIs	Higher probability of unplanned hospital admission in men with physical only multimorbidity in most (8.1%, 95%CI 7.3-9.1) versus least (5.4%, 95%CI 4.8-6.1)<br><br>Higher probability of unplanned hospital admission in men with physical and mental multimorbidity in most (15.1%, 95%CI 13.8-17.1) versus least (10.2%, 95%CI 9.2-11.7)
		≥ 4 LTCs	Most versus least deprived area quintile	Hospitalisation	Predicted probability and 95%CIs	Higher probability of unplanned hospital admission in men with physical only multimorbidity in most (13.0%, 95%CI 11.9-14.7) versus least (8.8%, 95%CI 7.9-9.9)<br><br>Higher probability of unplanned hospital admission in men with physical and mental multimorbidity in most (23.1%, 95%CI 21.2-26.2) versus least (16.1%, 95%CI 14.6-18.5)
Rurality	Pati et al[28]	≥ 2 LTCs	Rural versus urban	Inpatient and outpatient hospital use	IRR and 95%CIs	Urban area associated with increased likelihood of inpatient or outpatient hospital use compared to rural area (IRR 1.09, 95%CI 0.64-1.88) in people with multimorbidity

For peer review only - http://bmjopen.bmj.com/site/about/guidelines.xhtml

Payne et al,[30] a Scottish study rated as high quality, examined the association between SEP and hospital admission through regression analyses, stratified by gender, number of LTCs, and presence of physical only or physical and mental LTCs. Living in the most versus least deprived quintile areas was associated with a higher probability of hospitalisation for all groups examined (for example, men with physical only multimorbidity had a higher probability of unplanned hospital admission in most (6.4%, 95%CI 0.05.8-7.2) versus least (4.2%, 95%CI 3.8-4.7) deprived areas. These effects were more pronounced for people with more versus less LTCs, with a dose response seen between number of LTCs and increase in probability of hospital admission, and for people with physical and mental multimorbidity versus physical only multimorbidity.

Pati et al[28] examined the relationship between urban versus rural residence and any hospital attendance in India, and found a relationship that did not reach statistical significance (urban versus rural residence IRR 1.09, 95%CI 0.64-1.88).

Discussion

This systematic review and narrative synthesis describes existing evidence of associations between household and area exposures and ED attendance and hospitalisation outcomes in people with multimorbidity. In people with multimorbidity, household food insecurity was associated with hospitalisation, and rates of ED attendance and hospitalisation, and probability of hospitalisation, were higher in people living in the most versus least deprived areas. There is non-conclusive evidence that in people with multimorbidity, household income is associated with ED attendance and hospital admission, with differing relationships seen depending on study location and how healthcare is organised and paid for in different settings. No statistically significant relationships were found in people with multimorbidity between ED attendance or hospitalisation with marital status, living with others with multimorbidity, or living in urban versus rural areas.

Strengths of the study include comprehensive and systematic searching of many electronic databases, with an iteratively developed set of search terms, assessment of study quality with adaptation of a standard method to meet the needs of the review, and analysis using a standardised approach to narrative reporting. There are several limitations of our study. Firstly, there are few included studies because analyses of household and area variables associated with ED attendance or hospitalisation has not been commonly done in people with multimorbidity, and there is considerable heterogeneity in the definition and measurement of multimorbidity, exposures, outcomes, and analysis methods. Secondly, although multimorbidity was defined as the presence of ≥ 2 LTCs in all studies, there was a diverse range of LTCs included within counts, which will affect prevalence rates and therefore reliability of comparison

For peer review only

between studies.[18] Where individual studies examined the same exposure, different outcomes were used, precluding meta-analysis.

Household income was not conclusively associated with ED attendance or hospitalisation in people with multimorbidity, however small sample sizes resulting from stratified analyses may have contributed to some estimates not reaching statistical significance in the Canadian study examining this.[25] Different directions of association were found between household income and hospitalisation in different countries and healthcare systems by Wang et al.[32] Lower household income was associated with higher likelihood of hospitalisation in areas with universal healthcare provision (free at the point of care in Scotland and heavily subsidised in Hong Kong public hospitals), and lower likelihood in areas where healthcare is largely or solely privately funded (mainland China and the private hospitals in Hong Kong). These results are similar to findings from a study of older people (not stratified by multimorbidity) in Stockholm, where healthcare is free at the point of delivery, found women living in households with the lowest versus highest income households had an increased likelihood (OR 2.91, 95% CI 2.52-3.36) of ED attendance.[35] A similar study of Medicaid beneficiaries in the USA, found that having more social needs and lower income was associated with increased ED attendance and hospitalisation.[36]

Household hunger was associated with hospitalisation in both concordant and discordant multimorbidity in Tanzania,[31] which is similar to a study from the United States of America where household hunger was associated with increased hospitalisations in a general adult population (OR 1.36, 95%CI 1.22–1.52).[37] The severity of household hunger is likely to vary widely depending on study setting, making these findings context specific. Marital status is commonly examined as a proxy for not living alone and having increased household social support.[12, 38] However, actual living arrangements (including cohabitation as an unmarried couple) are more closely associated with health outcomes but are less examined due to data limitations.[38] We expected to see a protective effect between being married resulting in reduced ED attendance and hospitalisation, but the included studies found no statistically significant associations in both directions. The three studies examining area SES reported increased ED attendance and hospitalisation in people with multimorbidity living in the most deprived fifth of areas, consistent with existing literature of general adult populations people living in areas of lower area SES are higher users of healthcare.[8, 9, 39]

In our review, one study examined the association between living in urban versus rural areas and any hospital attendance in people with multimorbidity, finding a non-statistically significant OR>1 for people living in urban areas. Similarly, a study examining a population of older adults in China,[40] not stratified by multimorbidity status, found that living in urban areas was associated with increased likelihood of inpatient care.

For peer review only - http://bmjopen.bmj.com/site/about/guidelines.xhtml

Some of the variation in results across studies may be explained by the heterogeneity in the definition of multimorbidity. Future studies should standardise multimorbidity definitions (for example, the presence of two or more LTCs from a standardised list of LTCs) to align with other studies and improve comparability.[18] Comparative research that further explores variation in associations in different healthcare systems would be valuable, testing models of exposure to outcome associations. Such studies are needed to understand the mechanisms by which observed associations happen, examining whether household and area exposures interact with each other and with multimorbidity, considering whether these factors mediate, moderate, or have a multiplicative effect. Use of longitudinal study designs could be used to evaluate the evolution of increased risk or protection associated with the context in which an individual lives. Only two area-level exposures have been examined, despite more extensive literature examining the place-based determinants of multimorbidity,[17] emphasising the need to examine associations between outcomes in people with multimorbidity and a broader range of area-level factors, including green spaces, social cohesion, and provision of services.

In conclusion, although available evidence in the study area is limited, there is some evidence that household and area exposures are associated with increased risk of ED attendance and hospitalisation in people with multimorbidity. Since interventions to reduce hospital attendance may be more effective if they also account for the context in which people live, there is a need for further research to examine the contribution of a wider range of contextual exposures to hospital attendance in people with multimorbidity.

Word count: 3631

For peer review only

Declarations

Funding: CM received funding from the NHS Education for Scotland Academic Fellowship and Medical Research Council MR/W000253/1 to support this work.

Role of the funding source: The funder had no role development of study design, data collection and interpretation, or writing of the final report. The corresponding author had full access all data and had final responsibility for submitting for publication.

Conflict of interests: None.

Data Sharing Agreement: Not applicable. No data sharing agreement was obtained for this study because data analysed was obtained from previous published studies where data sharing agreements are in place.

Ethical Approvals: Not applicable. No ethical approval was obtained for this study because data analysed was obtained from previous published studies where ethical approvals are in place.

Author contributions: CM and BG conceived the study and contributed to the design of the project. CM created the search strategy and BG, SWM, JP, AL, and CD contributed to subsequent iterations. CM, HF, EL, and TC conducted the search. CM and BG extracted and analysed the data. CM drafted the paper, BG, SWM, JP, AM, AL, and CD critically commented on the manuscript. All authors reviewed drafts and approved the final version

For peer review only

References

1. National population projections: 2018-based. Office for National Statistics. Availalble at https://www.ons.gov.uk/peoplepopulationandcommunity/populationandmigration/populationprojections/bulletins/nationalpopulationprojections/2018based [accessed 19 September 2021].
2. Multimorbidity: a priority for global health research. The Academy of Medical Sciences. Available at https://acmedsci.ac.uk/policy/policy-projects/multimorbidity [accessed 19 September 2021].
3. Johansson S, Rosengren A, Young K, Jennings E. Mortality and morbidity trends after the first year in survivors of acute myocardial infarction: a systematic review. BMC cardiovascular disorders. 2017;17(1):53.
4. Jones PB. Adult mental health disorders and their age at onset. British journal of psychiatry. 2013;202(s54):s5-s10.
5. Hanlon P, Nicholl BI, Jani BD, Lee D, McQueenie R, Mair FS. Frailty and pre-frailty in middle-aged and older adults and its association with multimorbidity and mortality: a prospective analysis of 493 737 UK Biobank participants. The Lancet Public health. 2018;3(7):e323-e32.
6. Williams JS, Egede LE. The Association Between Multimorbidity and Quality of Life, Health Status and Functional Disability. The American journal of the medical sciences. 2016;352(1):45-52.
7. Cassell A, Edwards D, Harshfield A, Rhodes K, Brimicombe J, Payne R, et al. The epidemiology of multimorbidity in primary care: a retrospective cohort study. The British journal of general practice : the journal of the Royal College of General Practitioners. 2018;68(669):e245.
8. Luben R, Hayat S, Wareham N, Pharoah PP, Khaw KT. Sociodemographic and lifestyle predictors of incident hospital admissions with multimorbidity in a general population, 1999-2019: the EPIC-Norfolk cohort. Bmj Open. 2020;10(9).
9. McCormack LA, Madlock-Brown C. Social Determinant of Health Documentation Trends and Their Association with Emergency Department Admissions. AMIA Annual Symposium proceedings AMIA Symposium. [Article]. 2020;2020:823-32.
10. Huygen FJA. Family medicine : the medical life history of families / F.J.A. Huygen. London: Royal College of General Practitioners; 1990.
11. Longman JM, I Rolfe M, Passey MD, Heathcote KE, Ewald DP, Dunn T, et al. Frequent hospital admission of older people with chronic disease: A cross-sectional survey with telephone follow-up and data linkage. BMC Health Services Research. [Review]. 2012;12(1).
12. Ridge A, Peterson GM, Kitsos A, Seidel BM, Anderson V, Nash R. Potentially Preventable Hospitalisations in rural Community-Dwelling Patients. Internal medicine journal. 2021.
13. Bu F, Philip K, Fancourt D. Social isolation and loneliness as risk factors for hospital admissions for respiratory disease among older adults. Thorax. 2020;75(7):597-9.
14. Luben R, Hayat S, Khawaja A, Wareham N, Pharoah PP, Khaw K-T. Residential area deprivation and risk of subsequent hospital admission in a British population: the EPIC-Norfolk cohort. BMJ open. 2019;9(12):e031251-e.
15. Mohnen SM, Schneider S, Droomers M. Neighborhood characteristics as determinants of healthcare utilization – a theoretical model. Health economics review. 2019;9(1):1-9.
16. PRISMA: Transparent Reporting of Systematic Reviews and Meta-Analyses. Aavailable at http://www.prisma-statement.org [accessed 7 September 2021].
17. Ingram E, Ledden S, Beardon S, Gomes M, Hogarth S, McDonald H, et al. Household and area-level social determinants of multimorbidity: a systematic review. Journal of epidemiology and community health (1979). 2021;75(3):232-41.
18. Ho IS-S, Azcoaga-Lorenzo A, Akbari A, Black C, Davies J, Hodgins P, et al. Examining variation in the measurement of multimorbidity in research: a systematic review of 566 studies. The Lancet Public Health. 2021;6(8):e587-e97.

For peer review only - http://bmjopen.bmj.com/site/about/guidelines.xhtml

19. Stafford M, Deeny SR, Dreyer K, Shand J. Multiple long-term conditions within households and use of health and social care: a retrospective cohort study. BJGP open. 2021;5(2):BJGPO.2020.0134.
20. A conceptual framework for action on the social determinants of health: social determinants of health discussion paper 2. World Health Organisation. Available at https://apps.who.int/iris/bitstream/handle/10665/44489/9789241500852_eng.pdf?sequence=1&isAllowed=y [accessed 28 September 2021].
21. Innovation VH. Covidence systematic review software. Melbourne: Veritas Health Innovation VIC, 2016.
22. The Newcastle-Ottowa Assessment Scale (NOAS) for assessing the quality of nonrandomised studies in meta-analyses. Available at http://www.ohri.ca/programs/clinical_epidemiology/oxford.asp [accessed 5 October 2021].
23. Campbell M, McKenzie JE, Sowden A, Katikireddi SV, Brennan SE, Ellis S, et al. Synthesis without meta-analysis (SWiM) in systematic reviews: reporting guideline. BMJ. 2020;368:l6890-l.
24. Thomas JHJ. Cochrane Handbook for Systematic Reviews of Interventions. Cochrane Training. 2019, version 6.
25. Fisher KA, Griffith LE, Gruneir A, Upshur R, Perez R, Favotto L, et al. Effect of socio-demographic and health factors on the association between multimorbidity and acute care service use: population-based survey linked to health administrative data. Bmc Health Services Research. 2021 Jan;21(1).
26. Mbuya-Bienge C, Simard M, Gaulin M, Candas B, Sirois C. Does socio-economic status influence the effect of multimorbidity on the frequent use of ambulatory care services in a universal healthcare system? A population-based cohort study. Bmc Health Services Research. 2021 Mar;21(1).
27. Lu L, Zeng J, Zeng Z. What limits the utilization of health services among china labor force? analysis of inequalities in demographic, socio-economic and health status. International journal for equity in health. 2017;16(1):30-.
28. Pati S, Swain S, Hussain MA, Kadam S, Salisbury C. Prevalence, correlates, and outcomes of multimorbidity among patients attending primary care in Odisha, India. Annals of Family Medicine. [Article]. 2015;13(5):446-50.
29. Orueta JF, García-Álvarez A, Alonso-Morán E, Vallejo-Torres L, Nuño-Solinis R. Socioeconomic variation in the burden of chronic conditions and health care provision - Analyzing administrative individual level data from the Basque Country, Spain. BMC Public Health. [Article]. 2013;13(1).
30. Payne RA, Abel GA, Guthrie B, Mercer SW. The effect of physical multimorbidity, mental health conditions and socioeconomic deprivation on unplanned admissions to hospital: A retrospective cohort study. CMAJ. [Article]. 2013;185(5):E221-E8.
31. Tomita A, Leyna GH, Kim HY, Moodley Y, Mpolya E, Mogeni P, et al. Patterns of multimorbidity and their association with hospitalisation: a population-based study of older adults in urban Tanzania. Age and Ageing. 2021 Jul;50(4):1349-60.
32. Wang HHX, Wang JJ, Lawson KD, Wong SYS, Wong MCS, Li FJ, et al. Relationships of multimorbidity and income with hospital admissions in 3 health care systems. Annals of Family Medicine. [Article]. 2015;13(2):164-7.
33. Cassell A, Edwards D, Harshfield A, Rhodes K, Brimicombe J, Payne R, et al. The epidemiology of multimorbidity in primary care: A retrospective cohort study. British Journal of General Practice. [Article]. 2018;68(669):e245-e51.
34. Identifying property and street information. Available at https://www.gov.uk/government/publications/open-standards-for-government/identifying-property-and-street-information [accessed 19 March 2022].

For peer review only - http://bmjopen.bmj.com/site/about/guidelines.xhtml

35. Doheny M, Agerholm J, Orsini N, Schon P, Burstrom B. Socio-demographic differences in the frequent use of emergency department care by older persons: a population-based study in Stockholm County. Bmc Health Services Research. 2019 Mar;19.
36. Herrick CJM, Amy; Li, Linda; Verdecias, Niko; Brown, Derek S.; Kreuter, Matthew; Broussard, Darrell J.; Smith, Rachel E. Social Needs, Chronic Conditions, and Health Care Utilization among Medicaid Beneficiaries. Population health management 2021;24(6):681-90.
37. Wray CM, Tang J, Lopez L, Hoggatt K, Keyhani S. Association of Social Determinants of Health and Their Cumulative Impact on Hospitalization Among a National Sample of Community-Dwelling US Adults. Journal of general internal medicine : JGIM. 2021.
38. Herm A, Anson JON, Poulain M. Living arrangements and marital status: a register-based study of survival of older adults in Belgium at the beginning of the 21st century. Ageing and society. 2016;36(10):2141-62.
39. Giannouchos TV, Kum H-C, Foster MJ, Ohsfeldt RL. Characteristics and predictors of adult frequent emergency department users in the United States: A systematic literature review. Journal of evaluation in clinical practice. 2019;25(3):420-33.
40. Gong CH, Kendig H, He X. Factors predicting health services use among older people in China: An analysis of the China Health and Retirement Longitudinal Study 2013. BMC health services research. 2016;16(1):63-.
41. Barnett K, Mercer SW, Norbury M, Watt G, Wyke S, Guthrie B. Epidemiology of multimorbidity and implications for health care, research, and medical education: a cross-sectional study. The Lancet. 2012;380(9836):37-43.

For peer review only

For peer review only - http://bmjopen.bmj.com/site/about/guidelines.xhtml

Figure 1. Study selection, PRISMA diagram

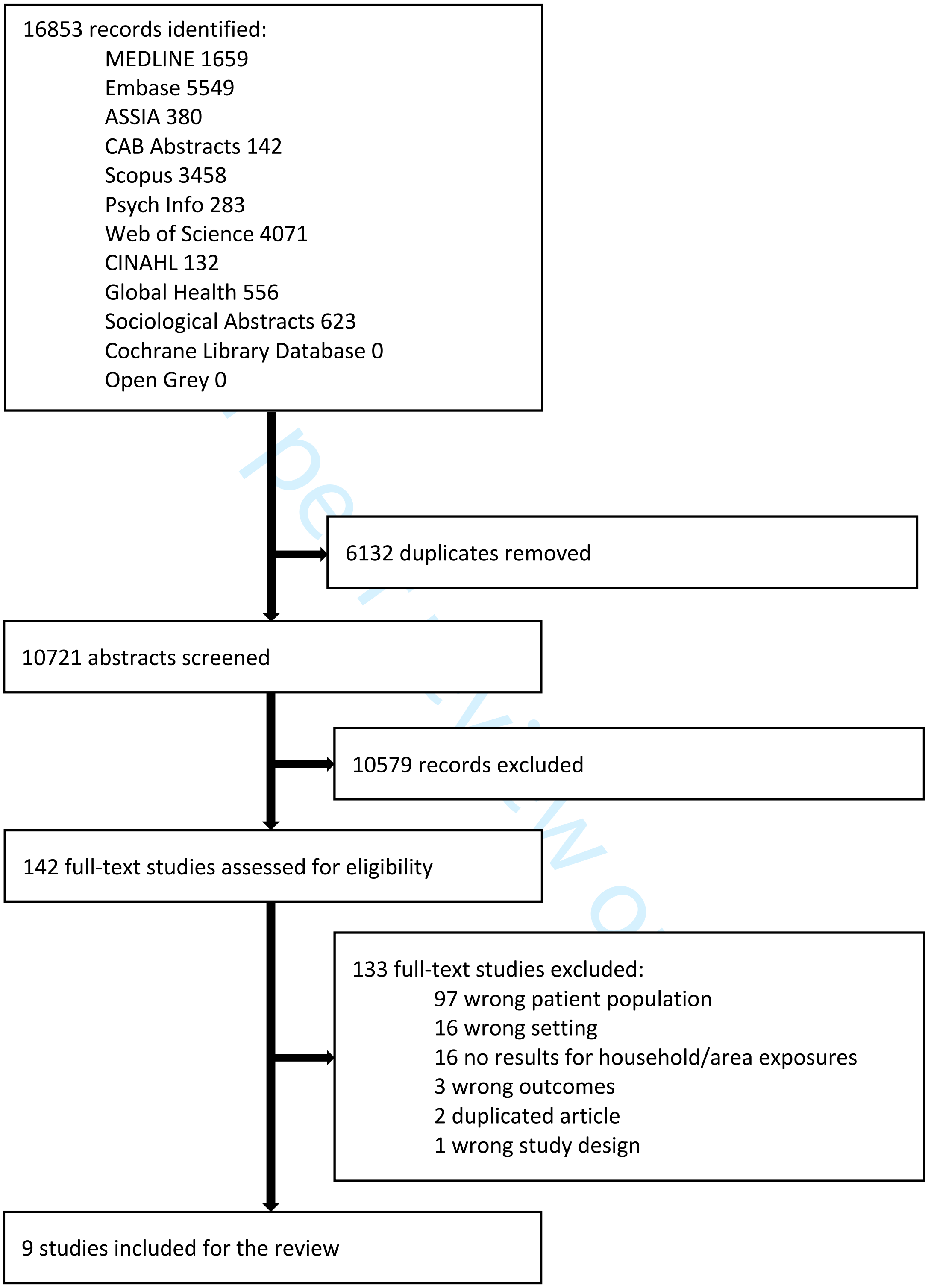

Supplementary File

Table S1. Database search strategies

**Database (number of articles)**	**Search strategy**
MedLine (1659)	(MULTIMORBIDITY/ or "multimorbidit*".mp. or "multi-morbidit*".mp. or "comorbidit*".mp. or "co-morbidit*".mp. or "polymorbidit*".mp. or "poly-morbidit*".mp. or"multicondition*".mp. or "multi-condition*".mp. or "multiple chronic condition*".mp. or "morbidity burden*".mp. or ((multiple or coexisting or co-existing or concurrent or con-current or comorbid* or co-morbid* or several*) adj2 (disease* or illness* or condition* or diagnosis* or morbid* or patholog*))) AND ("household*".mp. or "coresiden*".mp. or "co-residen*".mp. or ((household or family or domestic or residen*) adj2 (composition* or member* or characterist* or dweller*)) or "neighborhood*".mp. or "neighbourhood*".mp. or "small area*".mp. or "small-area*".mp. or "place".mp. or "communit*".mp. or "census tracts".mp. or "census block group".mp. or "zip code*".mp. or "post code*".mp. or "electoral ward*".mp. or "data zone*".mp. or "output area*".mp. or "lower super output area*".mp. or "municipalit*".mp. or "provinc*".mp. or SOCIOECONOMIC FACTORS/ or INCOME/ or "socioeconomic".mp. or "socio-economic".mp. or "social capital".mp. or "inequalit*".mp. or "disparit*".mp. or "income".mp. or "wealth".mp. or "financial problem*".mp. or "financial difficult*".mp. or "vocation*".mp. or "training opportunit*".mp. or "employment opportunit*".mp. or "occupation*".mp. or "opportunit*".mp. or "job opportunit*".mp. or "job insecurit".mp. or "education*" or "opportunit*".mp. or "education*".mp. or "achiev*".mp. or "education*".mp. or "quality".mp. or "fast-food".mp. or "fast food".mp. or "healthy food".mp. or "social determinant".mp. or "wider determinant".mp. or POVERTY AREAS/ or POVERTY/ or "neighbourhood disorder".mp. or "overcrowding".mp. or "public housing".mp. or "population density".mp. or "urban".mp. or "ghetto".mp. or "rural".mp. or "slum".mp. or "estate".mp. or "poverty area*".mp. or "physical environment*".mp. or "built environment*".mp. or "living standard*".mp. or "family structure".mp. or "family breakdown".mp. or "family disintegrat*".mp. or "single parent*".mp. or "housing tenure".mp. or "Townsend".mp. or "Carstairs".mp. or "Index of Multiple Deprivation".mp. or "Nam-Powers".mp. or "Hollingshead Index".mp. or "Breadline Britain Index".mp. or "inverse care law".mp. or "Singh ind*".mp. or "Yost ind*".mp. or "Kolak ind*".mp. or "social determinants of health".mp. or "neighbourhood archetype*".mp. or "segregation indic*".mp. or "ecological inversion".mp. or "ecological fallacy".mp. or "ecological bias".mp. or "depriv*".mp. or "impoverish*".mp. or "disadvantage*".mp. or "community cohesion".mp. or "community network".mp. or "community support".mp. or "transport* quality".mp. or "transport* services".mp. or "transport link*".mp. or "public transport*".mp. or "travel time".mp. or "community resource access*".mp. or "walkability".mp. or "public space".mp. or "green space".mp. or "open space".mp. or "recreation*".mp. or "leisure activit*".mp. or "food outlet*".mp. or "food environment".mp. or "air pollution".mp. or "particulate matter".mp. or "nitrogen dioxide".mp. or "sulphur dioxide".mp. or "sulfur dioxide".mp. or "carbon monoxide".mp. or "crime*".mp.) AND ("hospital admission*".mp. or "hospitalisation".mp. or "hospitalization".mp. or "emergency admission*".mp. or "unscheduled admission*".mp. or "unplanned admission*".mp. or "emergency department".mp. or "accident and emergency".mp.)<br>limit to (english language and yr="2000-Current")
EMBASE (5549)	("multimorbidit*".mp. or "multi-morbidit*".mp. or "comorbidit*".mp. or "co-morbidit*".mp. or "polymorbidit*".mp. or "poly-morbidit*".mp. or"multicondition*".mp. or "multi-condition*".mp. or "multiple chronic condition*".mp. or "morbidity burden*".mp. or "multiple long-term condition*".mp. or ((multiple or coexisting or co-existing or concurrent or con-current or comorbid* or co-morbid* or several*) adj2 (disease* or illness* or condition* or diagnosis* or morbid* or patholog*))) AND ("household*".mp. or "coresiden*".mp. or "co-residen*".mp. or ((household or family or domestic or residen*) adj2 (composition* or member* or characterist* or dweller*)) or "neighborhood*".mp. or "neighbourhood*".mp. or "small area*".mp. or "small-area*".mp. or "place".mp. or "communit*".mp. or "census tracts".mp. or "census block group".mp. or "zip code*".mp. or "post code*".mp. or "electoral ward*".mp. or "data zone*".mp. or "output area*".mp. or "lower super output area*".mp. or "municipalit*".mp. or "provinc*".mp. or "socioeconomic".mp. or "socio-economic".mp. or "social capital".mp. or "inequalit*".mp. or "disparit*".mp. or "income".mp. or "wealth".mp. or "financial problem*".mp. or "financial difficult*".mp. or "vocation*".mp. or "training opportunit*".mp. or "employment opportunit*".mp. or "occupation*".mp. or "opportunit*".mp. or "job opportunit*".mp. or "job insecurit".mp. or "education*" or "opportunit*".mp. or "education*".mp. or "achiev*".mp. or "education*".mp. or "quality".mp. or "fast-food".mp. or "fast food".mp. or "healthy food".mp. or "social determinant".mp. or "wider determinant".mp. or "neighbourhood disorder".mp. or "overcrowding".mp. or "public housing".mp. or "population density".mp. or "urban".mp. or "ghetto".mp. or "rural".mp. or "slum".mp. or "estate".mp. or "poverty area*".mp. or "physical environment*".mp. or "built environment*".mp. or "living standard*".mp. or "family structure".mp. or "family breakdown".mp. or "family disintegrat*".mp. or "single parent*".mp. or "housing tenure".mp. or "Townsend".mp. or "Carstairs".mp. or "Index of Multiple Deprivation".mp. or "Nam-Powers".mp. or "Hollingshead Index".mp. or "Breadline Britain Index".mp. or "inverse care law".mp. or "Singh ind*".mp. or "Yost ind*".mp. or "Kolak ind*".mp. or "social determinants of health".mp. or "neighbourhood archetype*".mp. or "segregation indic*".mp. or "ecological inversion".mp. or "ecological fallacy".mp. or "ecological bias".mp. or "depriv*".mp. or "impoverish*".mp. or "disadvantage*".mp. or "community cohesion".mp. or "community network".mp. or "community support".mp. or "transport* quality".mp. or "transport* services".mp. or "transport link*".mp. or "public transport*".mp. or "travel time".mp. or "community resource access*".mp. or "walkability".mp. or "public space".mp. or "green space".mp. or "open space".mp. or "recreation*".mp. or "leisure activit*".mp. or "food

For peer review only

	outlet*".mp. or "food environment".mp. or "air pollution".mp. or "particulate matter".mp. or "nitrogen dioxide".mp. or "sulphur dioxide".mp. or "sulfur dioxide".mp. or "carbon monoxide".mp. or "crime*".mp.) AND ("admission*".mp. or "hospital admission*".mp. or "hospitalisation".mp. or "hospitalization".mp. or "emergency admission*".mp. or "unscheduled admission*".mp. or "unplanned admission*".mp. or "emergency department".mp. or "accident and emergency".mp.)<br>limit to (english language and yr="2000-Current")
Global Health (556)	("multimorbidit*".mp. or "multi-morbidit*".mp. or "comorbidit*".mp. or "co-morbidit*".mp. or "polymorbidit*".mp. or "poly-morbidit*".mp. or"multicondition*".mp. or "multi-condition*".mp. or "multiple chronic condition*".mp. or "morbidity burden*".mp. or ((multiple or coexisting or co-existing or concurrent or con-current or comorbid* or co-morbid* or several*) adj2 (disease* or illness* or condition* or diagnosis* or morbid* or patholog*))) AND ("household*".mp. or "coresiden*".mp. or "co-residen*".mp. or ((household or family or domestic or residen*) adj2 (composition* or member* or characterist* or dweller*)) or "neighborhood*".mp. or "neighbourhood*".mp. or "small area*".mp. or "small-area*".mp. or "place".mp. or "communit*".mp. or "census tracts".mp. or "census block group".mp. or "zip code*".mp. or "post code*".mp. or "electoral ward*".mp. or "data zone*".mp. or "output area*".mp. or "lower super output area*".mp. or "municipalit*".mp. or "provinc*".mp. or "socioeconomic".mp. or "socio-economic".mp. or "social capital".mp. or "inequalit*".mp. or "disparit*".mp. or "income".mp. or "wealth".mp. or "financial problem*".mp. or "financial difficult*".mp. or "vocation*".mp. or "training opportunit*".mp. or "employment opportunit*".mp. or "occupation*".mp. or "opportunit*".mp. or "job opportunit*".mp. or "job insecurit".mp. or "education*" or "opportunit*".mp. or "education*".mp. or "achiev*".mp. or "education*".mp. or "quality".mp. or "fast-food".mp. or "fast food".mp. or "healthy food".mp. or "social determinant".mp. or "wider determinant".mp. or "neighbourhood disorder".mp. or "overcrowding".mp. or "public housing".mp. or "population density".mp. or "urban".mp. or "ghetto".mp. or "rural".mp. or "slum".mp. or "estate".mp. or "poverty area*".mp. or "physical environment*".mp. or "built environment*".mp. or "living standard*".mp. or "family structure".mp. or "family breakdown".mp. or "family disintegrat*".mp. or "single parent*".mp. or "housing tenure".mp. or "Townsend".mp. or "Carstairs".mp. or "Index of Multiple Deprivation".mp. or "Nam-Powers".mp. or "Hollingshead Index".mp. or "Breadline Britain Index".mp. or "inverse care law".mp. or "Singh ind*".mp. or "Yost ind*".mp. or "Kolak ind*".mp. or "social determinants of health".mp. or "neighbourhood archetype*".mp. or "segregation indic*".mp. or "ecological inversion".mp. or "ecological fallacy".mp. or "ecological bias".mp. or "depriv*".mp. or "impoverish*".mp. or "disadvantage*".mp. or "community cohesion".mp. or "community network".mp. or "community support".mp. or "transport* quality".mp. or "transport* services".mp. or "transport link*".mp. or "public transport*".mp. or "travel time".mp. or "community resource access*".mp. or "walkability".mp. or "public space".mp. or "green space".mp. or "open space".mp. or "recreation*".mp. or "leisure activit*".mp. or "food outlet*".mp. or "food environment".mp. or "air pollution".mp. or "particulate matter".mp. or "nitrogen dioxide".mp. or "sulphur dioxide".mp. or "sulfur dioxide".mp. or "carbon monoxide".mp. or "crime*".mp.) AND ("hospital admission*".mp. or "hospitalisation".mp. or "hospitalization".mp. or "emergency admission*".mp. or "unscheduled admission*".mp. or "unplanned admission*".mp. or "emergency department".mp. or "accident and emergency".mp.)<br>limit to (english language and yr="2000-Current")
PsychInfo (283)	("multimorbidit*".mp. or "multi-morbidit*".mp. or "comorbidit*".mp. or "co-morbidit*".mp. or "polymorbidit*".mp. or "poly-morbidit*".mp. or"multicondition*".mp. or "multi-condition*".mp. or "multiple chronic condition*".mp. or "morbidity burden*".mp. or ((multiple or coexisting or co-existing or concurrent or con-current or comorbid* or co-morbid* or several*) adj2 (disease* or illness* or condition* or diagnosis* or morbid* or patholog*))) AND ("household*".mp. or "coresiden*".mp. or "co-residen*".mp. or ((household or family or domestic or residen*) adj2 (composition* or member* or characterist* or dweller*)) or "neighborhood*".mp. or "neighbourhood*".mp. or "small area*".mp. or "small-area*".mp. or "place".mp. or "communit*".mp. or "census tracts".mp. or "census block group".mp. or "zip code*".mp. or "post code*".mp. or "electoral ward*".mp. or "data zone*".mp. or "output area*".mp. or "lower super output area*".mp. or "municipalit*".mp. or "provinc*".mp. or "socioeconomic".mp. or "socio-economic".mp. or "social capital".mp. or "inequalit*".mp. or "disparit*".mp. or "income".mp. or "wealth".mp. or "financial problem*".mp. or "financial difficult*".mp. or "vocation*".mp. or "training opportunit*".mp. or "employment opportunit*".mp. or "occupation*".mp. or "opportunit*".mp. or "job opportunit*".mp. or "job insecurit".mp. or "education*" or "opportunit*".mp. or "education*".mp. or "achiev*".mp. or "education*".mp. or "quality".mp. or "fast-food".mp. or "fast food".mp. or "healthy food".mp. or "social determinant".mp. or "wider determinant".mp. or "neighbourhood disorder".mp. or "overcrowding".mp. or "public housing".mp. or "population density".mp. or "urban".mp. or "ghetto".mp. or "rural".mp. or "slum".mp. or "estate".mp. or "poverty area*".mp. or "physical environment*".mp. or "built environment*".mp. or "living standard*".mp. or "family structure".mp. or "family breakdown".mp. or "family disintegrat*".mp. or "single parent*".mp. or "housing tenure".mp. or "Townsend".mp. or "Carstairs".mp. or "Index of Multiple Deprivation".mp. or "Nam-Powers".mp. or "Hollingshead Index".mp. or "Breadline Britain Index".mp. or "inverse care law".mp. or "Singh ind*".mp. or "Yost ind*".mp. or "Kolak ind*".mp. or "social determinants of health".mp. or "neighbourhood archetype*".mp. or "segregation indic*".mp. or "ecological inversion".mp. or "ecological fallacy".mp. or "ecological bias".mp. or "depriv*".mp. or "impoverish*".mp. or "disadvantage*".mp. or "community cohesion".mp. or "community network".mp. or "community support".mp. or "transport* quality".mp. or "transport* services".mp. or "transport link*".mp. or "public transport*".mp. or "travel time".mp. or "community resource access*".mp. or "walkability".mp. or "public space".mp. or "green space".mp. or "open space".mp. or "recreation*".mp. or "leisure activit*".mp. or "food outlet*".mp. or "food environment".mp. or "air pollution".mp. or "particulate matter".mp. or "nitrogen dioxide".mp. or "sulphur dioxide".mp. or "sulfur dioxide".mp. or "carbon monoxide".mp. or "crime*".mp.) AND

For peer review only

	("hospital admission*".mp. or "hospitalisation".mp. or "hospitalization".mp. or "emergency admission*".mp. or "unscheduled admission*".mp. or "unplanned admission*".mp. or "emergency department".mp. or "accident and emergency".mp.)<br>limit to (english language and yr="2000-Current")
ASSIA (380)	("multimorbidit*" or "multi-morbidit*" or "comorbidit*" or "co-morbidit*" or "polymorbidit*" or "poly-morbidit*" or "multicondition*" or "multi-condition*" or "multiple chronic condition*" or "morbidity burden*" or ((multiple or coexisting or co-existing or concurrent or con-current or comorbid* or co-morbid* or several*) adj2 (disease* or illness* or condition* or diagnosis* or morbid* or patholog*))) AND ("household*" or "coresiden*" or "co-residen*" or ((household or family or domestic or residen*) adj2 (composition* or member* or characterist* or dweller*)) or "neighborhood*" or "neighbourhood*" or "small area*" or "small-area*" or "place" or "communit*" or "census tracts" or "census block group" or "zip code*" or "post code*" or "electoral ward*" or "data zone*" or "output area*" or "lower super output area*" or "municipalit*" or "provinc*" or "socioeconomic" or "socio-economic" or "social capital" or "inequalit*" or "disparit*" or "income" or "wealth" or "financial problem*" or "financial difficult*" or "vocation*" or "training opportunit*" or "employment opportunit*" or "occupation* opportunit*" or "job opportunit*" or "job insecurit" or "education* opportunit*" or "education* achiev*" or "education* quality" or "fast-food" or "fast food" or "healthy food" or "social determinant" or "wider determinant" or "neighbourhood disorder" or "overcrowding" or "public housing" or "population density" or "urban" or "ghetto" or "rural" or "slum" or "estate" or "poverty area*" or "physical environment*" or "built environment*" or "living standard*" or "family structure" or "family breakdown" or "family disintegrat*" or "single parent*" or "housing tenure" or "Townsend" or "Carstairs" or "Index of Multiple Deprivation" or "Nam-Powers" or "Hollingshead Index" or "Breadline Britain Index" or "inverse care law" or "Singh ind*" or "Yost ind*" or "Kolak ind*" or "social determinants of health" or "neighbourhood archetype*" or "segregation indic*" or "ecological inversion" or "ecological fallacy" or "ecological bias" or "depriv*" or "impoverish*" or "disadvantage*" or "community cohesion" or "community network" or "community support" or "transport* quality" or "transport* services" or "transport link*" or "public transport*" or "travel time" or "community resource access*" or "walkability" or "public space" or "green space" or "open space" or "recreation*" or "leisure activit*" or "food outlet*" or "food environment" or "air pollution" or "particulate matter" or "nitrogen dioxide" or "sulphur dioxide" or "sulfur dioxide" or "carbon monoxide" or "crime*") AND ("hospital admission*" or "hospitalisation" or "hospitalization" or "emergency admission*" or "unscheduled admission*" or "unplanned admission*" or "emergency department" or "accident and emergency")<br>Publication date: after 01 January 2000
CAB Abstracts (142)	("multimorbidit*" or "multi-morbidit*" or "comorbidit*" or "co-morbidit*" or "polymorbidit*" or "poly-morbidit*" or "multicondition*" or "multi-condition*" or "multiple chronic condition*" or "morbidity burden*" or ((multiple or coexisting or co-existing or concurrent or con-current or comorbid* or co-morbid* or several*) adj2 (disease* or illness* or condition* or diagnosis* or morbid* or patholog*))) AND ("household*" or "coresiden*" or "co-residen*" or ((household or family or domestic or residen*) adj2 (composition* or member* or characterist* or dweller*)) or "neighborhood*" or "neighbourhood*" or "small area*" or "small-area*" or "place" or "communit*" or "census tracts" or "census block group" or "zip code*" or "post code*" or "electoral ward*" or "data zone*" or "output area*" or "lower super output area*" or "municipalit*" or "provinc*" or "socioeconomic" or "socio-economic" or "social capital" or "inequalit*" or "disparit*" or "income" or "wealth" or "financial problem*" or "financial difficult*" or "vocation*" or "training opportunit*" or "employment opportunit*" or "occupation* opportunit*" or "job opportunit*" or "job insecurit" or "education* opportunit*" or "education* achiev*" or "education* quality" or "fast-food" or "fast food" or "healthy food" or "social determinant" or "wider determinant" or "neighbourhood disorder" or "overcrowding" or "public housing" or "population density" or "urban" or "ghetto" or "rural" or "slum" or "estate" or "poverty area*" or "physical environment*" or "built environment*" or "living standard*" or "family structure" or "family breakdown" or "family disintegrat*" or "single parent*" or "housing tenure" or "Townsend" or "Carstairs" or "Index of Multiple Deprivation" or "Nam-Powers" or "Hollingshead Index" or "Breadline Britain Index" or "inverse care law" or "Singh ind*" or "Yost ind*" or "Kolak ind*" or "social determinants of health" or "neighbourhood archetype*" or "segregation indic*" or "ecological inversion" or "ecological fallacy" or "ecological bias" or "depriv*" or "impoverish*" or "disadvantage*" or "community cohesion" or "community network" or "community support" or "transport* quality" or "transport* services" or "transport link*" or "public transport*" or "travel time" or "community resource access*" or "walkability" or "public space" or "green space" or "open space" or "recreation*" or "leisure activit*" or "food outlet*" or "food environment" or "air pollution" or "particulate matter" or "nitrogen dioxide" or "sulphur dioxide" or "sulfur dioxide" or "carbon monoxide" or "crime*") AND ("hospital admission*" or "hospitalisation" or "hospitalization" or "emergency admission*" or "unscheduled admission*" or "unplanned admission*" or "emergency department" or "accident and emergency")<br>Publication date: after 01 January 2000
Web of Science (4071)	("multimorbidit*" or "multi-morbidit*" or "comorbidit*" or "co-morbidit*" or "polymorbidit*" or "poly-morbidit*" or "multicondition*" or "multi-condition*" or "multiple chronic condition*" or "morbidity burden*" or ((multiple or coexisting or co-existing or concurrent or con-current or comorbid* or co-morbid* or several*) adj2 (disease* or illness* or condition* or diagnosis* or morbid* or patholog*))) AND ("household*" or "coresiden*" or "co-residen*" or ((household or family or domestic or residen*) adj2 (composition* or member* or characterist* or dweller*)) or "neighborhood*" or "neighbourhood*" or "small area*" or "small-area*" or "place" or "communit*" or "census tracts" or "census block group" or "zip code*" or "post code*" or "electoral ward*" or "data zone*" or

For peer review only

	"output area*" or "lower super output area*" or "municipalit*" or "provinc*" or "socioeconomic" or "socio-economic" or "social capital" or "inequalit*" or "disparit*" or "income" or "wealth" or "financial problem*" or "financial difficult*" or "vocation*" or "training opportunit*" or "employment opportunit*" or "occupation* opportunit*" or "job opportunit*" or "job insecurit" or "education* opportunit*" or "education* achiev*" or "education* quality" or "fast-food" or "fast food" or "healthy food" or "social determinant" or "wider determinant" or "neighbourhood disorder" or "overcrowding" or "public housing" or "population density" or "urban" or "ghetto" or "rural" or "slum" or "estate" or "poverty area*" or "physical environment*" or "built environment*" or "living standard*" or "family structure" or "family breakdown" or "family disintegrat*" or "single parent*" or "housing tenure" or "Townsend" or "Carstairs" or "Index of Multiple Deprivation" or "Nam-Powers" or "Hollingshead Index" or "Breadline Britain Index" or "inverse care law" or "Singh ind*" or "Yost ind*" or "Kolak ind*" or "social determinants of health" or "neighbourhood archetype*" or "segregation indic*" or "ecological inversion" or "ecological fallacy" or "ecological bias" or "depriv*" or "impoverish*" or "disadvantage*" or "community cohesion" or "community network" or "community support" or "transport* quality" or "transport* services" or "transport link*" or "public transport*" or "travel time" or "community resource access*" or "walkability" or "public space" or "green space" or "open space" or "recreation*" or "leisure activit*" or "food outlet*" or "food environment" or "air pollution" or "particulate matter" or "nitrogen dioxide" or "sulphur dioxide" or "sulfur dioxide" or "carbon monoxide" or "crime*") AND ("hospital admission*" or "hospitalisation" or "hospitalization" or "emergency admission*" or "unscheduled admission*" or "unplanned admission*" or "emergency department" or "accident and emergency")<br>Language: English<br>Publication date: after 01 January 2000
Scopus (3458)	("multimorbidit*" or "multi-morbidit*" or "comorbidit*" or "co-morbidit*" or "polymorbidit*" or "poly-morbidit*" or "multicondition*" or "multi-condition*" or "multiple chronic condition*" or "morbidity burden*" or ((multiple or coexisting or co-existing or concurrent or con-current or comorbid* or co-morbid* or several*) adj2 (disease* or illness* or condition* or diagnosis* or morbid* or patholog*))) AND ("household*" or "coresiden*" or "co-residen*" or ((household or family or domestic or residen*) adj2 (composition* or member* or characterist* or dweller*)) or "neighborhood*" or "neighbourhood*" or "small area*" or "small-area*" or "place" or "communit*" or "census tracts" or "census block group" or "zip code*" or "post code*" or "electoral ward*" or "data zone*" or "output area*" or "lower super output area*" or "municipalit*" or "provinc*" or "socioeconomic" or "socio-economic" or "social capital" or "inequalit*" or "disparit*" or "income" or "wealth" or "financial problem*" or "financial difficult*" or "vocation*" or "training opportunit*" or "employment opportunit*" or "occupation* opportunit*" or "job opportunit*" or "job insecurit" or "education* opportunit*" or "education* achiev*" or "education* quality" or "fast-food" or "fast food" or "healthy food" or "social determinant" or "wider determinant" or "neighbourhood disorder" or "overcrowding" or "public housing" or "population density" or "urban" or "ghetto" or "rural" or "slum" or "estate" or "poverty area*" or "physical environment*" or "built environment*" or "living standard*" or "family structure" or "family breakdown" or "family disintegrat*" or "single parent*" or "housing tenure" or "Townsend" or "Carstairs" or "Index of Multiple Deprivation" or "Nam-Powers" or "Hollingshead Index" or "Breadline Britain Index" or "inverse care law" or "Singh ind*" or "Yost ind*" or "Kolak ind*" or "social determinants of health" or "neighbourhood archetype*" or "segregation indic*" or "ecological inversion" or "ecological fallacy" or "ecological bias" or "depriv*" or "impoverish*" or "disadvantage*" or "community cohesion" or "community network" or "community support" or "transport* quality" or "transport* services" or "transport link*" or "public transport*" or "travel time" or "community resource access*" or "walkability" or "public space" or "green space" or "open space" or "recreation*" or "leisure activit*" or "food outlet*" or "food environment" or "air pollution" or "particulate matter" or "nitrogen dioxide" or "sulphur dioxide" or "sulfur dioxide" or "carbon monoxide" or "crime*") AND ("hospital admission*" or "hospitalisation" or "hospitalization" or "emergency admission*" or "unscheduled admission*" or "unplanned admission*" or "emergency department" or "accident and emergency")<br>Publication date: after 01 January 2000
CINAHL (132)	("multimorbidit*" or "multi-morbidit*" or "comorbidit*" or "co-morbidit*" or "polymorbidit*" or "poly-morbidit*" or "multicondition*" or "multi-condition*" or "multiple chronic condition*" or "morbidity burden*" or ((multiple or coexisting or co-existing or concurrent or con-current or comorbid* or co-morbid* or several*) adj2 (disease* or illness* or condition* or diagnosis* or morbid* or patholog*))) AND ("household*" or "coresiden*" or "co-residen*" or ((household or family or domestic or residen*) adj2 (composition* or member* or characterist* or dweller*)) or "neighborhood*" or "neighbourhood*" or "small area*" or "small-area*" or "place" or "communit*" or "census tracts" or "census block group" or "zip code*" or "post code*" or "electoral ward*" or "data zone*" or "output area*" or "lower super output area*" or "municipalit*" or "provinc*" or "socioeconomic" or "socio-economic" or "social capital" or "inequalit*" or "disparit*" or "income" or "wealth" or "financial problem*" or "financial difficult*" or "vocation*" or "training opportunit*" or "employment opportunit*" or "occupation* opportunit*" or "job opportunit*" or "job insecurit" or "education* opportunit*" or "education* achiev*" or "education* quality" or "fast-food" or "fast food" or "healthy food" or "social determinant" or "wider determinant" or "neighbourhood disorder" or "overcrowding" or "public housing" or "population density" or "urban" or "ghetto" or "rural" or "slum" or "estate" or "poverty area*" or "physical environment*" or "built environment*" or "living standard*" or "family structure" or "family breakdown" or "family disintegrat*" or "single parent*" or "housing tenure" or "Townsend" or "Carstairs" or "Index of Multiple Deprivation" or "Nam-Powers" or "Hollingshead Index" or "Breadline Britain Index" or "inverse care law" or "Singh ind*" or "Yost ind*" or "Kolak ind*" or "social determinants of health" or "neighbourhood archetype*" or "segregation indic*" or "ecological inversion" or "ecological fallacy" or "ecological bias" or "depriv*" or "impoverish*" or "disadvantage*" or "community cohesion" or "community network" or

For peer review only

	"community support" or "transport* quality" or "transport* services" or "transport link*" or "public transport*" or "travel time" or "community resource access*" or "walkability" or "public space" or "green space" or "open space" or "recreation*" or "leisure activit*" or "food outlet*" or "food environment" or "air pollution" or "particulate matter" or "nitrogen dioxide" or "sulphur dioxide" or "sulfur dioxide" or "carbon monoxide" or "crime*") AND ("hospital admission*" or "hospitalisation" or "hospitalization" or "emergency admission*" or "unscheduled admission*" or "unplanned admission*" or "emergency department" or "accident and emergency")<br>Publication date: after 01 January 2000
Sociological Abstracts (623)	("multimorbidit*" or "multi-morbidit*" or "comorbidit*" or "co-morbidit*" or "polymorbidit*" or "poly-morbidit*" or "multicondition*" or "multi-condition*" or "multiple chronic condition*" or "morbidity burden*" or ((multiple or coexisting or co-existing or concurrent or con-current or comorbid* or co-morbid* or several*) adj2 (disease* or illness* or condition* or diagnosis* or morbid* or patholog*))) AND ("household*" or "coresiden*" or "co-residen*" or ((household or family or domestic or residen*) adj2 (composition* or member* or characterist* or dweller*)) or "neighborhood*" or "neighbourhood*" or "small area*" or "small-area*" or "place" or "communit*" or "census tracts" or "census block group" or "zip code*" or "post code*" or "electoral ward*" or "data zone*" or "output area*" or "lower super output area*" or "municipalit*" or "provinc*" or "socioeconomic" or "socio-economic" or "social capital" or "inequalit*" or "disparit*" or "income" or "wealth" or "financial problem*" or "financial difficult*" or "vocation*" or "training opportunit*" or "employment opportunit*" or "occupation* opportunit*" or "job opportunit*" or "job insecurit" or "education* opportunit*" or "education* achiev*" or "education* quality" or "fast-food" or "fast food" or "healthy food" or "social determinant" or "wider determinant" or "neighbourhood disorder" or "overcrowding" or "public housing" or "population density" or "urban" or "ghetto" or "rural" or "slum" or "estate" or "poverty area*" or "physical environment*" or "built environment*" or "living standard*" or "family structure" or "family breakdown" or "family disintegrat*" or "single parent*" or "housing tenure" or "Townsend" or "Carstairs" or "Index of Multiple Deprivation" or "Nam-Powers" or "Hollingshead Index" or "Breadline Britain Index" or "inverse care law" or "Singh ind*" or "Yost ind*" or "Kolak ind*" or "social determinants of health" or "neighbourhood archetype*" or "segregation indic*" or "ecological inversion" or "ecological fallacy" or "ecological bias" or "depriv*" or "impoverish*" or "disadvantage*" or "community cohesion" or "community network" or "community support" or "transport* quality" or "transport* services" or "transport link*" or "public transport*" or "travel time" or "community resource access*" or "walkability" or "public space" or "green space" or "open space" or "recreation*" or "leisure activit*" or "food outlet*" or "food environment" or "air pollution" or "particulate matter" or "nitrogen dioxide" or "sulphur dioxide" or "sulfur dioxide" or "carbon monoxide" or "crime*") AND ("hospital admission*" or "hospitalisation" or "hospitalization" or "emergency admission*" or "unscheduled admission*" or "unplanned admission*" or "emergency department" or "accident and emergency")<br>Publication date: after 01 January 2000
Cochrane (0)	("multimorbidit*" or "multi-morbidit*" or "comorbidit*" or "co-morbidit*" or "polymorbidit*" or "poly-morbidit*" or "multicondition*" or "multi-condition*" or "multiple chronic condition*" or "morbidity burden*" or ((multiple or coexisting or co-existing or concurrent or con-current or comorbid* or co-morbid* or several*) adj2 (disease* or illness* or condition* or diagnosis* or morbid* or patholog*)))
OpenGrey (0)	("multimorbidit*" or "multi-morbidit*" or "comorbidit*" or "co-morbidit*" or "polymorbidit*" or "poly-morbidit*" or "multicondition*" or "multi-condition*" or "multiple chronic condition*" or "morbidity burden*" or ((multiple or coexisting or co-existing or concurrent or con-current or comorbid* or co-morbid* or several*) adj2 (disease* or illness* or condition* or diagnosis* or morbid* or patholog*)))

For peer review only

Table S2. Study methodological quality ratings: Newcastle-Ottowa Assessment (NOAS), adapted for cross-sectional studies

Source	Selection				Comparability based on design and analysis	Outcome		Total
	Representativeness of the sample	Sample size	Non-respondents	Ascertainment of exposure		Assessment of outcome	Statistical test	
Lu et al (2017)	Half*	*	*	Half*	*	Half*	*	6.5 (Medium)
Fisher et al (2021)	Half*	*	*	Half*	**	*	*	7 (High)
Mbuya-Bienge et al (2021)	*	*	*	*	*	*	Half*	6.5 (Medium)
Oureta et al (2013)	*	*	Half*	*	*	*	Half*	6.5 (Medium)
Pati et al (20 15)	Half*	Half*	Half*	Half*	*	Half*	*	4.5(Medium)
Payne et al (2013)	*	*	*	*	**	*	*	8 (High)
Stafford et al (2020)	Half*	Half*	half*	*	*	*	*	5.5 (Medium)
Tomita et al (2021)	*	*	*	*	**	Half*	*	7.5 (High)
Wang et al	Half*	*	Half*	Half*	**	Half*	*	6 (Medium)

Methodological score: high = 7-8, medium = 4-6, low = 0-3

For peer review only

Box S1. Risk of bias Newcastle-Ottowa Assessment (NOAS), adapted for this study and application to examination of cross-sectional studies

A study can be awarded a maximum of one point for each numbered item within the Selection and Exposure categories. A maximum of two points can be given for the Comparability category.

Selection (maximum 4)

1. Representativeness of the sample

a. Truly representative of the average in the target population (random sample or whole population) *

b. Somewhat representative of the average in the target population (purposive sampling of representative healthcare sector areas or evidence that the sample is representative of the source population) half*

c. Selected group of users

d. No description of the sampling strategy

2. Sample size

a. Justified and satisfactory *

b. Adequately powered to detect a difference (at least 10 events per variable in multivariate analyses)*

c. Not justified

3. Non-respondents

a. Comparability between respondents and non-respondents characteristics is established, and the response rate is satisfactory (>60%)*

b. The response rate is unsatisfactory, or the comparability between respondents and non-respondents is unsatisfactory

c. No description of the response rate or the characteristics of the responders and non-responders

4. Risk factor exposure assessment

a. Assessment of exposure including type and duration from electronic health record or administrative database*

b. Assessment of exposure from questionnaire *

c. No description

Comparability (maximum 2)

5. The subjects in different outcome groups are comparable, based on the study design or analysis. Confounding factors are adjusted for

a. Study controls for multimorbidity and no multimorbidity, including age and gender adjustment**

b. Study controls for multimorbidity and no multimorbidity, without additional variable adjustment*

c. Study does not control for multimorbidity

Outcome (maximum 2)

6. Assessment of the outcome (emergency department or unscheduled hospital admission)

a. Electronic health record or health payment record account of healthcare use *

b. Reported healthcare use from questionnaire or no described data source half*

7. Statistical test

a. The statistical test used to analyse the data is clearly described and appropriate, and the measurement of the association is presented as either an OR, CI and P value or a beta coefficient, SE, and P value*

b. The statistical test is not appropriate, not described, or incomplete

For peer review only

Checkilsts

1. Preferred Reporting Items for Systematic Reviews and Meta-Analyses (PRISMA) 2020 Checklist

Section and Topic	Item #	Checklist item	Location where item is reported
**TITLE**			
Title	1	Identify the report as a systematic review.	Methods – paragraph 1 (Page 4)
**ABSTRACT**			
Abstract	2	See the PRISMA 2020 for Abstracts checklist.	See checklist below
**INTRODUCTION**			
Rationale	3	Describe the rationale for the review in the context of existing knowledge.	Introduction – paragraphs 1-3 (Page 4)
Objectives	4	Provide an explicit statement of the objective(s) or question(s) the review addresses.	Introduction – paragraphs 2-3 (Page 4)
**METHODS**			
Eligibility criteria	5	Specify the inclusion and exclusion criteria for the review and how studies were grouped for the syntheses.	Methods – paragraph 3, Table 1 (Page 5)
Information sources	6	Specify all databases, registers, websites, organisations, reference lists and other sources searched or consulted to identify studies. Specify the date when each source was last searched or consulted.	Methods – paragraph 1 (Page 5)
Search strategy	7	Present the full search strategies for all databases, registers and websites, including any filters and limits used.	Table S1
Selection process	8	Specify the methods used to decide whether a study met the inclusion criteria of the review, including how many reviewers screened each record and each report retrieved, whether they worked independently, and if applicable, details of automation tools used in the process.	Methods – paragraph 4 (Page 4)
Data collection process	9	Specify the methods used to collect data from reports, including how many reviewers collected data from each report, whether they worked independently, any processes for obtaining or confirming data from study investigators, and if applicable, details of automation tools used in the process.	Methods – paragraph 6 (Page 5)
Data items	10a	List and define all outcomes for which data were sought. Specify whether all results that were compatible with each outcome domain in each study were sought (e.g. for all measures, time points, analyses), and if not, the methods used to decide which results to collect.	Methods – paragraph 6 (Page 5)
	10b	List and define all other variables for which data were sought (e.g. participant and intervention characteristics, funding sources). Describe any assumptions made about any missing or unclear information.	Methods – paragraph 6 (Page 5)
Study risk of bias assessment	11	Specify the methods used to assess risk of bias in the included studies, including details of the tool(s) used, how many reviewers assessed each study and whether they worked independently, and if applicable, details of automation tools used in the process.	Methods – paragraph 5 (Page 5)
Effect measures	12	Specify for each outcome the effect measure(s) (e.g. risk ratio, mean difference) used in the synthesis or presentation of results.	Methods – paragraph 7

For peer review only

Section and Topic	Item #	Checklist item	Location where item is reported
			(Page 6)
Synthesis methods	13a	Describe the processes used to decide which studies were eligible for each synthesis (e.g. tabulating the study intervention characteristics and comparing against the planned groups for each synthesis (item #5)).	Methods – paragraph 7 (Page 6)
	13b	Describe any methods required to prepare the data for presentation or synthesis, such as handling of missing summary statistics, or data conversions.	Methods – paragraph 7 (Page 6)
	13c	Describe any methods used to tabulate or visually display results of individual studies and syntheses.	Methods – paragraph 7 (Page 6)
	13d	Describe any methods used to synthesize results and provide a rationale for the choice(s). If meta-analysis was performed, describe the model(s), method(s) to identify the presence and extent of statistical heterogeneity, and software package(s) used.	Methods – paragraph 7 (Page 5)
	13e	Describe any methods used to explore possible causes of heterogeneity among study results (e.g. subgroup analysis, meta-regression).	Methods – paragraph 7 (Page 5)
	13f	Describe any sensitivity analyses conducted to assess robustness of the synthesized results.	Not applicable
Reporting bias assessment	14	Describe any methods used to assess risk of bias due to missing results in a synthesis (arising from reporting biases).	Methods – paragraph 5 (Page 5)
Certainty assessment	15	Describe any methods used to assess certainty (or confidence) in the body of evidence for an outcome.	Methods – paragraph 7 (Page 5)
**RESULTS**			
Study selection	16a	Describe the results of the search and selection process, from the number of records identified in the search to the number of studies included in the review, ideally using a flow diagram.	Results – paragraph 1 (Page 6)
	16b	Cite studies that might appear to meet the inclusion criteria, but which were excluded, and explain why they were excluded.	Introduction – paragraph 2 (Page 4)
Study characteristics	17	Cite each included study and present its characteristics.	Results – paragraphs 2-4 and 7-12 (Pages 6-8), Table 2
Risk of bias in studies	18	Present assessments of risk of bias for each included study.	Results – paragraph 8 (Page 7), Table S2
Results of individual studies	19	For all outcomes, present, for each study: (a) summary statistics for each group (where appropriate) and (b) an effect estimate and its precision (e.g. confidence/credible interval), ideally using structured tables or plots.	Results – paragraphs 3-16 (Page 6-10), Table 3
Results of syntheses	20a	For each synthesis, briefly summarise the characteristics and risk of bias among contributing studies.	Results – paragraphs 8-16 (Page 7-10).

For peer review only

Section and Topic	Item #	Checklist item	Location where item is reported
	20b	Present results of all statistical syntheses conducted. If meta-analysis was done, present for each the summary estimate and its precision (e.g. confidence/credible interval) and measures of statistical heterogeneity. If comparing groups, describe the direction of the effect.	Results – paragraphs 9-16 (Page 8-10)
	20c	Present results of all investigations of possible causes of heterogeneity among study results.	Results – paragraphs 9-16 (Page 8-10)
	20d	Present results of all sensitivity analyses conducted to assess the robustness of the synthesized results.	Not applicable
Reporting biases	21	Present assessments of risk of bias due to missing results (arising from reporting biases) for each synthesis assessed.	Table S2
Certainty of evidence	22	Present assessments of certainty (or confidence) in the body of evidence for each outcome assessed.	Results – paragraphs 9-16 (Page 8-10)
**DISCUSSION**			
Discussion	23a	Provide a general interpretation of the results in the context of other evidence.	Discussion - paragraph 1 (Page 10)
	23b	Discuss any limitations of the evidence included in the review.	Discussion – paragraph 2 (Page 10)
	23c	Discuss any limitations of the review processes used.	Discussion – paragraph 2 (Page 10)
	23d	Discuss implications of the results for practice, policy, and future research.	Discussion – paragraph 6 (Page 11-12)
**OTHER INFORMATION**			
Registration and protocol	24a	Provide registration information for the review, including register name and registration number, or state that the review was not registered.	Methods – paragraph 1 (Page 4)
	24b	Indicate where the review protocol can be accessed, or state that a protocol was not prepared.	Methods – paragraph 1 (Page 4)
	24c	Describe and explain any amendments to information provided at registration or in the protocol.	Not applicable
Support	25	Describe sources of financial or non-financial support for the review, and the role of the funders or sponsors in the review.	Declarations (Page 13)
Competing interests	26	Declare any competing interests of review authors.	Declarations (Page 13)
Availability of data, code and other materials	27	Report which of the following are publicly available and where they can be found: template data collection forms; data extracted from included studies; data used for all analyses; analytic code; any other materials used in the review.	Stable 2, Table 3, upplementary file

From: Page MJ, McKenzie JE, Bossuyt PM, Boutron I, Hoffmann TC, Mulrow CD, et al. The PRISMA 2020 statement: an updated guideline for reporting systematic reviews. BMJ 2021;372:n71. doi: 10.1136/bmj.n71

For peer review only

2. PRISMA 2020 for Abstracts Checklist

Section and Topic	Item #	Checklist item	Reported (Yes/No)
**TITLE**			
Title	1	Identify the report as a systematic review.	Background
**BACKGROUND**			
Objectives	2	Provide an explicit statement of the main objective(s) or question(s) the review addresses.	Methods
**METHODS**			
Eligibility criteria	3	Specify the inclusion and exclusion criteria for the review.	Methods
Information sources	4	Specify the information sources (e.g. databases, registers) used to identify studies and the date when each was last searched.	Methods (and main paper)
Risk of bias	5	Specify the methods used to assess risk of bias in the included studies.	No (main paper)
Synthesis of results	6	Specify the methods used to present and synthesise results.	Methods
**RESULTS**			
Included studies	7	Give the total number of included studies and participants and summarise relevant characteristics of studies.	No (main paper)
Synthesis of results	8	Present results for main outcomes, preferably indicating the number of included studies and participants for each. If meta-analysis was done, report the summary estimate and confidence/credible interval. If comparing groups, indicate the direction of the effect (i.e. which group is favoured).	Results
**DISCUSSION**			
Limitations of evidence	9	Provide a brief summary of the limitations of the evidence included in the review (e.g. study risk of bias, inconsistency and imprecision).	No (main paper)
Interpretation	10	Provide a general interpretation of the results and important implications.	Results
**OTHER**			
Funding	11	Specify the primary source of funding for the review.	No (main paper)
Registration	12	Provide the register name and registration number.	Methods

From: Page MJ, McKenzie JE, Bossuyt PM, Boutron I, Hoffmann TC, Mulrow CD, et al. The PRISMA 2020 statement: an updated guideline for reporting systematic reviews. BMJ 2021;372:n71. doi: 10.1136/bmj.n71
For more information, visit: http://www.prisma-statement

For peer review only

3. Synthesis Without Meta-analysis (SWiM) reporting items

**SWiM is intended to complement and be used as an extension to PRISMA**			
**SWiM reporting item**	**Item description**	**Page in manuscript where item is reported**	**Other***
Methods			
1 Grouping studies for synthesis	1a) Provide a description of, and rationale for, the groups used in the synthesis (e.g., groupings of populations, interventions, outcomes, study design)	Methods – paragraph 7 (Pages 5-6)	
	1b) Detail and provide rationale for any changes made subsequent to the protocol in the groups used in the synthesis		Not applicable
2 Describe the standardised metric and transformation methods used	Describe the standardised metric for each outcome. Explain why the metric(s) was chosen, and describe any methods used to transform the intervention effects, as reported in the study, to the standardised metric, citing any methodological guidance consulted	Results – paragraph 5-16 (Pages 7-10)	
3 Describe the synthesis methods	Describe and justify the methods used to synthesise the effects for each outcome when it was not possible to undertake a meta-analysis of effect estimates	Methods – paragraphs 5 and 7 (Pages 5-6)	
4 Criteria used to prioritise results for summary and synthesis	Where applicable, provide the criteria used, with supporting justification, to select the particular studies, or a particular study, for the main synthesis or to draw conclusions from the synthesis (e.g., based on study design, risk of bias assessments, directness in relation to the review question)	Results – paragraph 5-16 (Pages 7-10)	
5 Investigation of heterogeneity in reported effects	State the method(s) used to examine heterogeneity in reported effects when it was not possible to undertake a meta-analysis of effect estimates and its extensions to investigate heterogeneity	Results – paragraph 5-16 (Pages 7-10)	
6 Certainty of evidence	Describe the methods used to assess certainty of the synthesis findings		Not applicable
7 Data presentation methods	Describe the graphical and tabular methods used to present the effects (e.g., tables, forest plots, harvest plots).<br>Specify key study characteristics (e.g., study design, risk of bias) used to order the studies, in the text and any tables or graphs, clearly referencing the studies included	Table 2, Table 3,	
Results			
8 Reporting results	For each comparison and outcome, provide a description of the synthesised findings, and the certainty of the findings. Describe the result in language that is consistent with the question the synthesis addresses, and indicate which studies contribute to the synthesis	Results – paragraph 5-16 (Pages 7-10)	
Discussion			
9 Limitations of the synthesis	Report the limitations of the synthesis methods used and/or the groupings used in the synthesis, and how these affect the conclusions that can be drawn in relation to the original review question	Discussion – paragraph 2 (Page 10)	

For peer review only

BMJ Open

Household and area determinants of emergency department attendance and hospitalisation in people with multimorbidity: a systematic review

Journal:	*BMJ Open*
Manuscript ID	bmjopen-2022-063441.R1
Article Type:	Original research
Date Submitted by the Author:	17-Jul-2022
Complete List of Authors:	MacRae, Clare; The University of Edinburgh Usher Institute of Population Health Sciences and Informatics,<br>Fisken, Harry; The University of Edinburgh Edinburgh Medical School<br>Lawrence, Edward; Ninewells Hospital and Medical School<br>Connor, Thomas; The University of Edinburgh Edinburgh Medical School<br>Pearce, Jamie; University of Edinburgh Institute of Geography, Institute of Geography<br>Marshall, Alan; The University of Edinburgh Social Policy, Social Policy<br>Lawson, Andrew; Medical University of South Carolina, Department of Public Health Sciences<br>Dibben, Chris; University of Edinburgh Institute of Geography<br>Mercer, Stewart; The University of Edinburgh, The Usher Institute of Population Health Sciences and Informatics<br>Guthrie, Bruce; The University of Edinburgh Usher Institute of Population Health Sciences and Informatics
**Primary Subject Heading**:	Public health
Secondary Subject Heading:	Epidemiology
Keywords:	EPIDEMIOLOGY, PUBLIC HEALTH, STATISTICS & RESEARCH METHODS

SCHOLARONE™
Manuscripts

I, the Submitting Author has the right to grant and does grant on behalf of all authors of the Work (as defined in the below author licence), an exclusive licence and/or a non-exclusive licence for contributions from authors who are: i) UK Crown employees; ii) where BMJ has agreed a CC-BY licence shall apply, and/or iii) in accordance with the terms applicable for US Federal Government officers or employees acting as part of their official duties; on a worldwide, perpetual, irrevocable, royalty-free basis to BMJ Publishing Group Ltd ("BMJ") its licensees and where the relevant Journal is co-owned by BMJ to the co-owners of the Journal, to publish the Work in this journal and any other BMJ products and to exploit all rights, as set out in our licence.

The Submitting Author accepts and understands that any supply made under these terms is made by BMJ to the Submitting Author unless you are acting as an employee on behalf of your employer or a postgraduate student of an affiliated institution which is paying any applicable article publishing charge ("APC") for Open Access articles. Where the Submitting Author wishes to make the Work available on an Open Access basis (and intends to pay the relevant APC), the terms of reuse of such Open Access shall be governed by a Creative Commons licence – details of these licences and which Creative Commons licence will apply to this Work are set out in our licence referred to above.

Other than as permitted in any relevant BMJ Author's Self Archiving Policies, I confirm this Work has not been accepted for publication elsewhere, is not being considered for publication elsewhere and does not duplicate material already published. I confirm all authors consent to publication of this Work and authorise the granting of this licence.

For peer review only

Household and area determinants of emergency department attendance and hospitalisation in people with multimorbidity: a systematic review

Authors: Clare MacRae[1], Harry Fisken[2], Edward Lawrence[3], Thomas Connor[2], Jamie Pearce[4], Alan Marshall[5], Andrew Lawson[1, 6], Chris Dibben[4], Stewart W Mercer[1], Bruce Guthrie[1]

[1]The University of Edinburgh Usher Institute of Population Health Sciences and Informatics University of Edinburgh, EH8 9AG Edinburgh, UK EH8 9AG

[2] The University of Edinburgh Edinburgh Medical School Edinburgh, Edinburgh, UK

[3] Ninewells Hospital and Medical School Dundee, Dundee, UK

[4] University of Edinburgh Institute of Geography, Institute of Geography Edinburgh, Edinburgh, UK

[5] The University of Edinburgh Social Policy, Social Policy Edinburgh, Edinburgh, UK

[6] Medical University of South Carolina, Department of Public Health Sciences Charleston, USA

Corresponding author: Dr Clare MacRae, Usher Institute, College of Medicine and Veterinary Medicine, University of Edinburgh, EH8 9AG, clare.macrae@ed.ac.uk

PROSPERO registration number: CRD42021283515

For peer review only

Abstract

Objectives: Multimorbidity is one of the biggest challenges facing healthcare internationally. Emergency department (ED) attendance and hospitalisation rates are higher in people with multimorbidity, but most research focuses on associations with individual characteristics, ignoring household or area mediators of service use.

Design: Systematic review reported using the synthesis without meta-analysis framework.

Data sources: Twelve electronic databases (January 1st, 2000, to September 21st, 2021): MEDLINE/OVID, Embase, Global Health, PsycINFO, ASSIA, CAB Abstracts, Science Citation Index Expanded/ISI Web of Science, Scopus, Cumulative Index to Nursing and Allied Health Literature [CINAHL], Sociological Abstracts, the Cochrane Library, and OpenGrey.

Eligibility criteria: Adults aged ≥16 years, with multimorbidity. Exposure(s) were household and/or area determinants of health. Outcomes were ED attendance and/or hospitalisation.

Data extraction and synthesis: Independent double screening of titles and abstracts to select relevant full-text studies. Methdological quality was assessed using an adaptation of the Newcastle-Ottowa Quality Assessment Scale tool. Given high study heterogeneity, narrative synthesis was performed. PROSPERO registration number CRD42021283515.

Results: After de-duplication, 10,721 titles and abstracts were screened, and 142 full-text articles reviewed, of which ten were eligible for inclusion. In people with multimorbidity, household food insecurity was associated with hospitalisation (OR 1.58 [95%CI 1.06-2.36] in concordant multimorbidity). People with multimorbidity living in the most versus least deprived areas attended ED more frequently (8.9% [95%CI 8.6-9.1] in most, versus 6.3% [95%CI 6.1-6.6] in least), had higher rates of hospitalisation (26% in most, versus 22% in least), and higher probability of hospitalisation (6.4% [95%CI 5.8-7.2] in most, versus 4.2% [95%CI 3.8-4.7] in least). There was non-conclusive evidence that household income is associated with ED attendance and hospitalisation. No statistically significant relationships were found between marital status, living with others with multimorbidity, or rurality and ED attendance or hospitalisation.

Conclusions: There is some evidence that household and area context mediate associations of multimorbidity with ED attendance and hospitalisation, but firm conclusions are constrained by the small number of studies published and study design heterogeneity. Further research is required on large population samples using robust analytical methods.

For peer review only - http://bmjopen.bmj.com/site/about/guidelines.xhtml

Word count: 334

For peer review only - http://bmjopen.bmj.com/site/about/guidelines.xhtml

Strengths and limitations

- Comprehensive literature search of twelve electronic databases to examine associations between household or area context and hospital attendance, specifically in people with multimorbidity
- Reporting as per Preferred Reporting Items for Systematic Reviews and Meta-Analyses and Synthesis Without Meta-analysis guidelines
- Limited evidence available in current literature with a small number of studies meeting eligibility criteria
- Heterogeneity in multimorbidity definition and measurement, and study exposures, outcomes, and analysis methods, precluded meta-analysis

For peer review only

Introduction

Multimorbidity is usually defined as the presence of two or more long-term physical or mental health conditions and is one of the biggest challenges facing healthcare internationally. It is becoming more common globally because of population ageing,[1, 2] improved survival after acute illnesses such as myocardial infarction,[3] and changing patterns of mental health in younger people.[4] There are large independent associations between multimorbidity and adverse outcomes including frailty,[5] reduced functional health status,[6] and hospital attendance.[7] People with multimorbidity are at nearly three times the risk of hospitalisation compared to those without.[7]

Research usually compares different groups of people with multimorbidity, for example defined by their individual characteristics, such as age, sex, and lifestyle factors.[8, 9] However, for this important patient group, social, and environmental contexts are rarely examined in depth. People live in a social context, where attitudes and practices can be influenced by those around them, for example households have been described as "a living and developing unit of interdependent members, sharing common internal and external conditions and interactions".[10] Research examining general adult populations (not stratified by multimorbidity) has found that people who live in a household with fewer financial or food resources,[11, 12] and single person households,[13] are at higher risk of emergency department (ED) attendance and hospitalisation. The areas in which people live can influence health. For example, populations not stratified by multimorbidity living in urban areas, and in areas with low socioeconomic status (SES), with associated additional health burdens, are similarly at risk.[14] Households and areas are therefore an important unit of measurement when considering patterns of disease and healthcare use.[15] To date, there is a paucity of synthesis of studies examining these data in people with multimorbidity. Additionally, factors such as geographical proximity to services are known to be associated with likelihood of ED attendance,[16] and consideration of individual characteristics and variation in how services themselves operate, including supply-wide factors such as relative accessibility of primary care versus ED care are important.

Understanding whether and how context influences outcomes is needed to ensure that the call by the Academy of Medical Sciences for research into "how to organise healthcare systems to better manage patients with multimorbidity" is based on appropriate understanding of both the individual and their social and environmental context.[2] The aim of this review is to examine, in people with multimorbidity, associations of household and area contextual exposures with emergency department (ED) attendance and hospitalisation.

For peer review only - http://bmjopen.bmj.com/site/about/guidelines.xhtml

Methods

Method development and reporting of findings of the systematic review were based on the PRISMA-P 2020 checklist.[17] The protocol was registered with PROSPERO CRD42021283515.

Twelve electronic databases (MEDLINE/OVID, Embase, Global Health, PsycINFO, ASSIA, CAB Abstracts, Science Citation Index Expanded/ISI Web of Science, Scopus, Cumulative Index to Nursing and Allied Health Literature [CINAHL], Sociological Abstracts, the Cochrane Library, and OpenGrey), and reference lists, were searched for full text articles meeting eligibility criteria, published between 1st January 2000, and 21st September 2021, which was the date the final searches took place. Searches were limited to articles written in English. An empirical approach to deriving the search terms was followed using a test set of three critical papers, one examining the household and area determinants of multimorbidity,[18] a second examining the definition and operationalisation of the term multimorbidity,[19] and a third meeting the study inclusion and exclusion criteria.[20] Key search terms, derived through reference to medical subject heading terms in test papers, iterated within MedLine to maximise sensitivity and specificity for relevant articles, were divided into three sets pertaining to multimorbidity, contextual exposures, and healthcare use (Table S1).

Eligible studies included populations of adults aged 16 years and over, from the general population, who had been assessed for, and had, the presence of multimorbidity or multiple long-term conditions (LTCs), defined according to the Academy of Medical Sciences core definition of multimorbidity as 'two or more chronic conditions' (Table 1).[2] Study exposure(s) examined were one or more household and/or area determinant of health, including built environment, and socioeconomic determinants of health (Table S1).[21] Study outcomes were ED attendance and/or hospitalisation.

For peer review only

For peer review only - http://bmjopen.bmj.com/site/about/guidelines.xhtml

1 **Table 1. Study inclusion and exclusion criteria**

2

	Inclusion	Exclusion
**Population**	Adult participants from the general population residing in the community, assessed for the presence of multiple LTCs (multimorbidity) aged 16 years and older	Participants initially selected based on the presence of index diseases, including any study examining comorbidity<br>Studies exclusively examining children aged 15 years and younger<br>Participants within hospital settings, or examination of readmission where the dominator is previously hospitalised participants
**Exposure**	Study exposure(s): ≥ 1 household or area-level socioeconomic determinant of health (SDoH) in alignment with the World Health Organisation (WHO) Commission on SDoH (CSDH) Framework[21]<br>Household: One residential unit, characteristics can include reference to the built environment, or the occupants living within<br>Area: Geographical area within which a person lives<br>All area sizes larger than household unit	Study exposure(s) include individual SDoH only (e.g., ethnicity). Study exposure(s) are direct "causes" of ill-health, such as health behaviours (e.g., smoking)
**Comparator**	Study reports comparator group(s) for SDoH exposures (e.g., prevalence of hospitalisation admission in lowest versus highest household income)	Study does not report a comparator group for SDoH exposure(s)
**Outcome**	Prevalence or incidence studies examining emergency department use and hospitalisation (defined as an overnight admission)	Studies not examining emergency department use or hospitalisation
**Study design**	Peer-reviewed studies of quantitative research designs (cross-sectional and longitudinal)	Systematic reviews, meta-analyses, clinical trials, and qualitative research

3

4

5

6

For peer review only - http://bmjopen.bmj.com/site/about/guidelines.xhtml

Following removal of duplicate records, independent double screening for relevance was performed for all titles and abstracts, using Covidence software,[22] and relevant full-text studies were selected (Figure 1). Conflicts were resolved through discussion between screeners (CM, HF, EL, TC) at each stage of screening and eligibility of all included studies was verified by a fifth author (BG).

The Newcastle-Ottowa Quality Assessment Scale (NOAS) tool[23] was adapted (Box S1), and study quality screening for each paper was performed independently by two of the researchers (CM plus one of HF, EL, TC), with consensus achieved through discussion.

CM performed data extraction using a custom spreadsheet to record study design, location, population (age, definition of multimorbidity), exposures (household and/or area), and outcome measures (ED attendance and/or hospitalisation). If data were missing or unclear, we contacted corresponding authors for clarification who provided additional data, and information regarding study methodology and analyses.

Study methodologies, exposures, outcomes, and effect measures were diverse and heterogenous, and narrative synthesis was therefore performed according to Synthesis Without Meta-analysis (SWiM)[24] and PRISMA guidelines.[17] Studies were grouped according to exposure examined, to ensure meaningful presentation of reviewed evidence.[25] Presentation of results using a standardised metric was not possible given the heterogeneity of outcome measures. Due to the small number of included studies, all study results were reported with quantitative figures given for statistically significant findings. Narrative comparison of heterogeneity between studies examining each exposure was performed by reviewing results within each group, comparing methodologies, exposures, and outcomes. Patients or the public were not involved in the design, conduct, reporting, or dissemination plans of this research.

Patient and Public Involvement

Patients or the public were not involved in the design, conduct, reporting, or dissemination plans of this research.

Results

The systematic search identified 16,853 articles. After removal of 6,132 duplicates, 10,721 titles and abstracts were screened, of which 10,579 were excluded at the first screening stage. Full text-screening of 142 articles identified 10 studies meeting eligibility criteria. Screening of reference lists yielded no additional studies eligible for inclusion (Figure 1).

For peer review only - http://bmjopen.bmj.com/site/about/guidelines.xhtml

Study characteristics

Two studies were conducted in North America,[26, 27] three in Asia,[28-30] three in Europe,[20, 31, 32] one in Africa, [33] and one study used multiple sites[34] (Table 2). All studies used cross-sectional data; two used data derived from existing cohort studies.[26, 27] Study sample size ranged from 1,670 to 5,316,830 (median 27,689 participants, interquartile range [IQR] 20,689-216,633). Seven studies reporting regression model outcomes,[20, 26, 28-30, 33] used smaller populations (1,670-162,464 participants, median 24,642, IQR 15,387-66,474), than the two studies reporting rates of hospital attendance (2,262,698-5,316,830, median 3,789,764, IQR 3,026,231-4,553,297).[27, 31] Each included study examined a specific age-group, which were ≥15 years old (one study),[30] 15-65 years old (one study),[28] ≥18 years old (two studies),[27, 29] ≥20 years old (two studies),[32, 34] ≥40 years old (one study),[33] ≥50 years old (one study),[20] and ≥65 years old (two studies).[26, 31]

For peer review only - http://bmjopen.bmj.com/site/about/guidelines.xhtml

Table 2. Study characteristics.

Author	Study purpose	Study design	Data source a	Sample size	Setting and date	Target population	Multimorbidity definition	Household exposure	Area exposure	Primary outcome measure(s)[a]
Chung et al (2016),[30] Hong Kong	Examine factors associated with hospitalisation	Cross-sectional	Hong Kong Government household survey	25 780	General population; 2011	≥ 15 yrs	≥ 2 LTCSs out of 45 LTCs, mental and physical	Household income and marital status	NA	12-month hospitalisation[b]
Fisher et al (2021),[26] Canada	Examine socio-demographic and health factors associated with acute care	Cross-sectional	Canadian Community Health Survey; administrative health data	28 361	General population; pooled from multiple cycles 2005-2012	65 - 85 yrs	≥ 2 LTCSs out of 12 LTCs, physical and mental	Household income	NA	12-month ED attendance and hospitalisation[b]
Lu et al (2017),[28] China	Examine inequalities limiting utilisation of health services	Cross-sectional	China Labour Force Dynamic Survey	23 505	Adult population in employment; 2014	15-65 yrs	≥ 2 LTCSs out of 10 LTCs, physical only	Marital status	NA	12-month hospitalisation[b]
Mbuya-Bienge et al (2021),[27] Canada	Examine SES and frequency of use of healthcare	Cross-sectional	Quebec Integrated Chronic Disease System	5 316 830	General population; 2011-2012	≥18 yrs	≥ 2 LTCSs out of 31 LTCs, physical and mental	NA	Area SES	12-month ED attendance
Orueta et al (2013),[31] Spain	Examine costs of multimorbidity by SES	Cross-sectional	Routine administrative health data	2 262 698	General population: 2011	≥65 yrs	≥ 2 LTCSs out of 52 LTCs, physical and mental	NA	Area SES	12-month hospitalisation[b]
Pati et al (20 15),[29] India	Examine relationship between multimorbidity and health care utilisation	Cross-sectional	Multi-stage random sampling patient interviews	1 670	General population, 2013-2014	≥18 yrs	≥ 2 LTCSs out of 22 LTCs, physical and mental	Marital status	Rurality	12-month hospital in-patient or out-patient hospital use
Payne et al (2013),[32] Scotland,	Examine association between SES and	Cross-sectional	Scottish Practice Team	180 815	General population; 2006	≥18 yrs	≥ 2 LTCSs out of 52 LTCsphysical and mental	NA	Area SES	12-month emergency or potentially

For peer review only - http://bmjopen.bmj.com/site/about/guidelines.xhtml

China, Hong Kong	unplanned hospitalisation		Information dataset							preventable hospitalisation
Stafford et al (2020),[20] England	Examine association between co-resident multimorbidity status an hospitalisation	Cross-sectional	Barking and Dagenham (B&D) health data, Clinical Practice Research Datalink (CPRD)	9 222 (B&D) and 10 528 (CPRD)	General population; 2016-2018	≥ 50 yrs living in 2-person household s	≥ 2 LTCSs out of 16 LTCs, physical and mental	Multimorbidity status of other household member	NA	Non-elective hospitalisation
Tomita et al (2021),[33] Tanzania	Examine association between SES and hospitalisation	Cross-sectional	Dar es Salaam Health Demographic Surveillance System	2 299	General population; 2017-2018	≥ 40 yrs	≥ 2 LTCSs out of 8 LTCs (concordant and discordant)[c], physical and mental	Marital status<br><br>Household food insecurity	NA	12-month hospitalisation[b]
Wang et at (2015),[34] Scotland, China, Hong Kong	Examine association between household income with hospitalisation	Cross-sectional	Scotland: Health Survey<br>China: Routine administrative data<br>Hong Kong: Household Survey	36 921<br><br>162 464<br><br>29 187	General population; 2008 to 2011<br>2011<br><br>2011	≥ 40 yrs	≥ 2 LTCSs out of 31 LTCs, physical and menta	Household income	NA	12-month hospitalisation[b]

[a] All outcomes are dichotomous (attendance or no attendance) rather than counts
[b] No differentiation planned/unplanned
[c] Concorant multimorbidity: 1 domain of mental health/non-communicable/communicable health. Discordant multimorbidity: ≥ domains.

For peer review only - http://bmjopen.bmj.com/site/about/guidelines.xhtml

Multimorbidity was defined as the presence of two or more LTCs by all studies, with the number of LTCs included in multimorbidity measures ranging from 8 to 52 (median 26.5, IQR 13-41.5). Nine studies included both physical and mental LTCs,[20, 26, 27, 29-35] and one study only included physical LTCs.[28] Various approaches were taken to defining the list of LTCs included, ranging from no included rationale by Lu et al[28] and Stafford et al,[20] adaptation of previous studies examining multimorbidity by Oureta et al,[31] to including LTCs included in the Charlson and Elixhauser indexes by Mbuya-Bienge et al.[27] Tomita et al[33] examined both concordant and discordant multimorbidity, fitting separate logistic regression models for each. In this study, discordant multimorbidity was defined as the occurrence of LTCs in ≥ two domains in mental health, non-communicable, and communicable disease.

The 10 included studies examined six unique exposures, where an exposure was defined as any household or area characteristic associated with ED attendance or hospitalisation. Seven studies examined household,[20, 26, 28-30, 33, 34] and four studies examined area exposures.[27, 29, 31, 32] One study, by Pati et al,[29] examined both household and area exposures.

Household exposures examined were household income (three studies),[26, 30, 34] multimorbidity status of other household members (one study),[20] household food insecurity (one study),[33] and marital status (four studies).[28-30, 33] Data on household income were derived through household surveys in all included studies.[26, 30, 34] Household food insecurity was defined using a cross-cultural measure, where survey participants were asked how often there was no food in their household, and how often household members went to sleep hungry or did not eat for a day.[33] Multimorbidity status of other household members was determined through linkage of local government and health provider administrative data with the unique property reference number,[20] a unique identifier for every addressable location in the United Kingdom.[36] Marital status was the most examined household exposure, but studies varied in how they examined this. Pati et al[29] classified participants as currently married and currently not married, and Lu et al[28] defined groups as married and single/divorced. Tomita et al[33] and Chung et al[30] divided participants into three groups comparing those who are currently married with never married, or separated/divorced/widowed. Stafford et al[20] examined multimorbidity status of household the other household resident in two-person households, with no reference to relationship status.

Area exposures examined were area SEP (three studies),[27, 31, 32] and rurality (one study).[29] All studies examining area SEP used a similar approach to deriving their SEP measure, using quintiles of a score derived from census tract deprivation indexes, based on rates of unemployment, educational attainment, and type of employment. Rurality was examined as a dichotomous exposure of rural versus urban, derived from population size and density, and proportion of workers.

For peer review only - http://bmjopen.bmj.com/site/about/guidelines.xhtml

Reported outcomes were any ED attendance (two studies),[26, 27] any hospitalisation (whether planned or unplanned) (six studies),[26, 28, 30, 31, 33, 34] any inpatient or outpatient hospital attendance (one study),[29] any ED attendance or unplanned hospitalisation (one study),[20] and any unplanned or emergency hospitalisation or unplanned or emergency potentially avoidable hospitalisation (one study).[32] No studies presented results stratified by the number of attendances in the study period.

Methodological quality of studies varied across studies and was rated high in three studies,[26, 32, 33] and medium in six[20, 27-29, 31, 34] (Table S2). Potential bias related to reporting of hospital attendance outcomes, where studies used either questionnaire based self-reported outcomes,[28, 29, 33, 34] or electronic health records and health payment systems,[20, 26, 27, 31] therefore differences in accuracy and reporting could exist between these studies. Use of statistical reporting varied between studies, with six studies performing regression models with different model outcomes, including odds ratios (ORs),[20, 26, 28, 33] incidence rate ratios (IRRs),[29] event probability,[34] as well as studies examining unadjusted rates of hospital attendance.[27, 31]

Household income

There was inconclusive evidence of a relationship between household income and hospital use. Chung et al,[30] a medium quality study from Hong Kong, examined the relative risk (RR) of hospitalisation for households with different levels of income. Analyses were compared to household income of <$10K and RRs were close to 1.00 for all income strata, with all 95%CIs crossing 1.00 apart (from income of $10-19K, RR1.66, 95%CI 1.002-1.356). Fisher et al,[26] a Canadian study rated as high quality, examined associations between household income and service use in people with multimorbidity, with separate regression models fitted to sub-groups defined by age-group (65-74 and 75-84 years), sex, multimorbidity (categorised as 2-3 and ≥4 LTCs), and outcomes (ED attendance and hospitalisation) (Table 3). In people with multimorbidity, ORs for ED attendance in people with middle (Canadian $30-$79.9K) and lower (<$30K) household income, versus high (>$80K), were almost all >1 (15 out of 16). However, 12 (out of 16) had wide confidence intervals crossing 1, which is likely to reflect small numbers in each stratum. There was some evidence of a dose-response relationship (ORs for low versus high income were always larger than ORs for medium versus high income), and estimated ORs were all small to moderate in size (maximum likelihood in men aged 65-74 with ≥4 LTCs was OR 2.74, 95C%I 1.12-6.66 for low versus high household income). In models with hospitalisation as outcome, the ORs for middle and lower household income, versus high, were all >1, however only two (out of 16) ORs had 95%CIs not crossing 1, and a similar dose-response relationship between degree of income and hospitalisation was seen.

For peer review only - http://bmjopen.bmj.com/site/about/guidelines.xhtml

Table 3. Household exposure study results

Study exposure	Study	Outcome	Methodological quality rating	Covariates	Result metric	Key Results
Household income	Chung et al[30] China	Hospitalisation	Medium	No details provided	Relative risk (RR) and 95%CIs	Highest RR of hospitalisation in households with income ≥$50,000 (RR 1.193 [0.916–1.553]) versus income <$10,000 (reference RR 1.000)
	Fisher et al[26] Canada	ED attendance and any hospital use	High	Stratified by age and number of LTCs	ORs and 95%CIs	Highly stratified sample with small group sizes. 6 of 32 models showed statistically significant relationship between increased likelihood of ED attendance/any hospital use with lower household income .
	Wang et al[34] Scotland, Hong Kong, China	Hospitalisation	Medium	Stratified by number of LTC and sex	Predicted probability (P) and 95%CIs	Scotland: Higher probability of hospitalisation in lowest (18.7% [95%CI 18.3-19.1]) versus highest (11.1% [95%CI 10.7-11.4]) household income<br>China (no medical insurance): Lower probability of hosptial admission in lowest (8.2% [95%CI 7.9-8.5]) versus highest (13.1% [95%CI 12.2-14.0]) household income
Co-resident multimorbidity	Stafford et al[20] England	ED attendance	Medium	Age, sex, SES	ORs and 95%CIs	No difference in ED attendance between people with household co-resident with versus without multimorbidity (OR 1.08 [95%CI 0.95-1.23])
Household food insecurity	Tomita et al[33] Tanzania	Hospitalisation	High	Age, sex, marital status, education	ORs and 95%CIs	Increased likelihood of hospitalisation in severe versus little-to-no household food insecurity (OR 1.58 [95%CI 1.06-2.36])
Household marital status	Chung et al[30] China	Hospitalisation	Medium	No details provided	Relative risk (RR) and 95%CIs	Highest RR of hospitalisation in widowed (RR 1.058 [95%CI 0.791-1.416], lowest RR in married (RR0.917 [95%CI 0.706-1.191]) versus single (reference RR 1.000)
	Lu et al[28] China	Hospitalisation	Medium	No details provided	ORs and 95%CIs	No difference in hospitalisation between married or single/divorced (OR 1.04 [95%CI 0.54-2.02])
	Pati et al[29] India	Inpatient and outpatient hospital use	Medium	Age, sex, ethnicity, education, SES rurality	IRRs and 95%CIs	No difference in inpatient or outpatient hospital use between currently married versus currently not married (IRR 1.17 [95%CI 0.85-1.61])
	Tomita et al[33] Tanzania	Hospitalisation	High	Age, sex, education	ORs and 95%CIs	No difference in hospitalisation between currently married versus never married (OR 1.43 [95%CI 0.62-3.28]), or separated/divorced (OR 1.24 [95%CI 0.86-1.78])

[*] See Table 2 for definition of concordant and discordant multimorbidity

For peer review only - http://bmjopen.bmj.com/site/about/guidelines.xhtml

Wang et al,[34] a study rated as medium quality, found that in people with multimorbidity, lower household income was associated with a higher probability of hospitalisation in Scotland, and public hospitalisation in Hong Kong. A dose-response relationship was seen where probability of admission rose as the number of LTCs increased, for example, probability for hospitalisation was higher in people with ≥4 LTCs (probability 30.7%, 95%CI 30.3-31.7) than people with 2 LTCs (18.85%, 95%CI 18.4-19.2) in the lowest household income group in Scotland. However, a reversed relationship, lower household income associated with lower probability of hospitalisation, was seen in China and in private hospitals in Hong Kong. For example, people with lowest household income had lowest probability of hospitalisation (24.7%, 95%CI 23.4-26.1) versus people with highest household income (36.1% [95%CI 33.6-38.6]), in people with ≥4 LTCs with no medical insurance in China, and the pattern was the same regardless of medical insurance status.

Household co-resident multimorbidity status

An English study by Stafford et al,[20] examined the multimorbidity status of the other household resident in two-person households. In this study rated as medium quality, no significant difference between living with someone who had multimorbidity, versus someone who did not have multimorbidity, was found in association with ED attendance (OR 1.08 [95%CI 0.95-1.23]).

Household hunger

A statistically significant relationship between severe household hunger and hospitalisation was found in a Tanzanian study by Tomita et al,[33] rated as high quality. This relationship was found in both concordant (severe versus none-to-moderate household hunger OR 1.58 [95%CI 1.06-2.36]) and discordant (severe versus none-to-moderate household hunger OR 1.54 [95%CI 1.04-2.28]) multimorbidity.

Marital status

No significant association between marital status and any outcome was found in the four studies examining this. Chung et al[30] examined relative RR of hospitalisation depending on marital status, and there were no statistically significant results with all RR being close to 1.00 with wide 95%CIs crossing 1.00. Lu et al,[28] a Chinese study rated as medium quality, found a small but not statistically significant relationship between being married and hospitalisation (married versus unmarried OR 1.04 [95%CI 0.54-2.02]). Pati et al,[29] a study from India rated as medium quality, found a larger but not statistically significant relationship in the opposite direction between marital status and any inpatient or outpatient hospital attendance (unmarried versus married incidence rate ratio [IRR] 1.17 [95%CI 0.85-1.61]). Similarly, Tomita et al[33] found a small but not statistically significant association between not being married and hospitalisation in people with concordant multimorbidity (never married versus married OR

For peer review only - http://bmjopen.bmj.com/site/about/guidelines.xhtml

1.43 [95%CI 0.62-3.28]; divorced/separated vs married OR 1.24 [95%CI 0.86-1.7 [) and in people with discordant multimorbidity (never married versus married OR 1.44 [95%CI 0.63-3.28]; divorced/separated versus married OR 1.27 [95%CI 0.83-1.85]) in Tanzania.

Socioeconomic status

More people living in the most versus least deprived quintile of areas attended ED in a medium quality Canadian study by Mbuya-Bienge et al[27] (Table 4). Results were stratified by number of LTCs: for people with two LTCs 8.9% (95%CI 8.6-9.1) living in most deprived areas attended ED versus 6.3% (95%CI 6.1-6.6) in the least deprived areas; for people with three LTCs 12.0% (95%CI 11.6-12.5) versus 9.5% (95%CI 9.1-10.0); for people with ≥ four LTCs 18.3% (95%CI 17.9-18.8) versus 16.8% (95%CI 16.3-17.4). Likewise, more people living in the most versus least deprived area quintiles were hospitalised in the Spanish study by Orueta et al,[31] rated as medium quality. This study also stratified the number of LTCs and found that in people with four to six LTCs, 26% living in most deprived areas were hospitalised versus 22% of people in the least deprived areas; for people with seven to nine LTCs, 48% versus 44% (no 95%CIs or cohort size from which to derive these). However, in people with ≥10 LTCs, rates of hospitalisation were lower in people living in the most, 68%, versus least, 70%, deprived areas. Neither study examining the effect of SES performed regression analyses.

For peer review only - http://bmjopen.bmj.com/site/about/guidelines.xhtml

Table 4. Area exposure study results

Study exposure	Study	Outcome	Methodological quality rating	Covariates	Result metric	Key Results
Area SEP	Mbuya-Bienge et al[27] Canada	ED attendance	Medium	Non-adjusted prevalence rates	Prevalence % and 95%CIs	Lower rates of ED use in most (8.9% [95%CI 8.6-9.1]) versus least (6.3% [95%CI 6.1-6.6]) deprived area quintile
	Orueta et al[31] Spain	Hospitalisation	Medium	Non-adjusted prevalence rates	Prevalence % (no 95%CIs)	Lower rates of hospitalisation in most (26%) versus least (22%) deprived area quintile
	Payne et al[32] Scotland, Hong Kong, China	Hospitalisation	High	Stratified by number of LTC and sex	Predicted probability and 95%CIs	Higher probability of unplanned hospitalisation in men with physical only multimorbidity in most (6.4% [95%CI 5.8-7.2]) versus least (4.2% [95%CI 3.8-4.7])<br>Higher probability of unplanned hospitalisation in men with physical and mental multimorbidity in most (12.1% [95%CI 11.0-13.6]) versus least (8.1% [95%CI 7.3-9.2])
Rurality	Pati et al[29] India	Inpatient and outpatient hospital use	Medium	Age, sex, ethnicity, education, SES, rurality	IRR and 95%CIs	Urban area associated with increased likelihood of inpatient or outpatient hospital use compared to rural area (IRR 1.09 [95%CI 0.64-1.88])

For peer review only

Payne et al,[32] a Scottish study rated as high quality, examined the association between SEP and hospitalisation through regression analyses, stratified by gender, number of LTCs, and presence of physical only or physical and mental LTCs. Living in the most versus least deprived quintile areas was associated with a higher probability of hospitalisation for all groups examined (for example, men with physical only multimorbidity had a higher probability of unplanned hospitalisation in most (6.4% [95%CI 0.05.8-7.2]) versus least (4.2% [95%CI 3.8-4.7]) deprived areas. These effects were more pronounced for people with more versus less LTCs, with a dose response seen between number of LTCs and increase in probability of hospitalisation, and for people with physical and mental multimorbidity versus physical only multimorbidity.

Area rurality

Pati et al[29] examined the relationship between urban versus rural residence and any hospital attendance in India, and found a relationship that did not reach statistical significance (urban versus rural residence IRR 1.09 [95%CI 0.64-1.88]).

Discussion

Principle findings

This systematic review and narrative synthesis describe existing evidence of associations between household and area exposures and ED attendance and hospitalisation outcomes in people with multimorbidity. In people with multimorbidity, household food insecurity was associated with hospitalisation, and rates of ED attendance and hospitalisation, and probability of hospitalisation, were higher in people living in the most versus least deprived areas. There is non-conclusive evidence that in people with multimorbidity, household income is associated with ED attendance and hospitalisation, with differing relationships seen depending on study location and how healthcare is organised and paid for in different settings. No statistically significant relationships were found in people with multimorbidity between ED attendance or hospitalisation with marital status, living with others with multimorbidity, or living in urban versus rural areas.

Strengths and weaknesses

Strengths of the study include comprehensive and systematic searching of many electronic databases, with an iteratively developed set of search terms, extraction of data by two researchers, and assessment of study quality with adaptation of a standard method to meet the needs of the review, and analysis using a standardised approach to narrative reporting. There are several limitations of our study. There are few included studies because analyses of household and area variables associated with ED attendance or hospitalisation has not been commonly done in people with multimorbidity, and there is considerable heterogeneity in the definition and measurement of multimorbidity, exposures, outcomes,

For peer review only - http://bmjopen.bmj.com/site/about/guidelines.xhtml

and analysis methods precluding meta-analysis. Studies were from both high-income and low-and-middle income countries, where contextual factors may hold different meanings, making direct comparisons across studies difficult. Finally, although multimorbidity was defined as the presence of ≥ 2 LTCs in all studies, there was a diverse range of LTCs included within counts, which will affect prevalence rates and therefore reliability of comparison between studies.[19]

Comparison with existing literature

Household income was not conclusively associated with ED attendance or hospitalisation in people with multimorbidity, however small sample sizes resulting from stratified analyses may have contributed to some estimates not reaching statistical significance in the Canadian study examining this.[26] Different directions of association were found between household income and hospitalisation in different countries and healthcare systems by Wang et al.[34] Lower household income was associated with higher likelihood of hospitalisation in areas with universal healthcare provision (free at the point of care in Scotland and heavily subsidised in Hong Kong public hospitals). These results reflect findings from a study of older people (not stratified by multimorbidity) in Stockholm, where healthcare is free at the point of delivery, found women living in households with the lowest versus highest income households had an increased probability (OR 2.91 [95% CI 2.52-3.36]) of ED attendance.[37] However lower household income was associated with lower probability of hospitalition in areas where healthcare is largely or solely privately funded (mainland China and the private hospitals in Hong Kong), demonstrating that lack of access to equitable care can further exacerbate health inequalities by reducing access to those with lowest household income.[34]

Household hunger was associated with hospitalisation in both concordant and discordant multimorbidity in Tanzania,[33] which is similar to a study from the United States of America where household hunger was associated with increased hospitalisations in a general adult population (OR 1.36 [95%CI 1.22–1.52]).[38] The severity of household hunger is likely to vary widely depending on study setting, making these findings context specific. Marital status in this study was assumed to be a household variable. Marital status could also be considered a characteristic of the individual or of a household. It is commonly examined as a proxy for not living alone and having increased household social support in studies using routine data,[12, 39] and in this study was therefore assumed to be a household variable." However, actual living arrangements (including cohabitation as an unmarried couple) are more closely associated with health outcomes but are less examined due to data limitations.[39] We expected to see a protective effect between being married resulting in reduced ED attendance and hospitalisation, but the included studies found no statistically significant associations in both directions. The three studies examining area SES reported increased ED attendance and hospitalisation in people with multimorbidity living in the

For peer review only

For peer review only - http://bmjopen.bmj.com/site/about/guidelines.xhtml

most deprived fifth of areas, consistent with existing literature of general adult populations people living in areas of lower area SES are higher users of healthcare.[8, 9, 40]

In our review, one study examined the association between living in urban versus rural areas and any hospital attendance in people with multimorbidity, finding a non-statistically significant OR>1 for people living in urban areas. Similarly, a study examining a population of older adults in China,[41] not stratified by multimorbidity status, found that living in urban areas was associated with increased likelihood of inpatient care.

Implications

Some of the variation in results across studies may be explained by the heterogeneity in the definition of multimorbidity. Future studies should standardise multimorbidity definitions (for example, the presence of two or more LTCs from a standardised list of LTCs) to align with other studies and improve comparability.[19] Comparative research that further explores variation in associations in different healthcare systems would be valuable, testing models of exposure to outcome associations. Such studies are needed to understand the mechanisms by which observed associations happen, examining whether household and area exposures interact with each other and with multimorbidity, considering whether these factors mediate, moderate, or have a multiplicative effect. No included articles examined supply factors associated with hospital use, for example area wide demand issues such as distance to services, or supply issues such as access to primary care services. These factors are likely to influence hospital use rates in people with multimorbidity and require further study. Use of longitudinal study designs could be used to evaluate the evolution of increased risk or protection associated with the context in which an individual lives. Only two area-level exposures have been examined, despite more extensive literature examining the place-based determinants of multimorbidity,[18] emphasising the need to examine associations between outcomes in people with multimorbidity and a broader range of area-level factors, including green spaces, social cohesion, and provision of services.

Conclusions

In conclusion, although available evidence in the study area is limited, there is some evidence that household and area exposures are associated with increased risk of ED attendance and hospitalisation in people with multimorbidity. Since interventions to reduce hospital attendance may be more effective if they also account for the context in which people live, there is a need for further research to examine the contribution of a wider range of contextual exposures to hospital attendance in people with multimorbidity.

Word count: 3956

For peer review only - http://bmjopen.bmj.com/site/about/guidelines.xhtml

Figure 1. Study selection, PRISMA diagram

For peer review only

For peer review only - http://bmjopen.bmj.com/site/about/guidelines.xhtml

Declarations

Funding: CM received funding from the NHS Education for Scotland Academic Fellowship and Medical Research Council MR/W000253/1 to support this work.

Role of the funding source: The funder had no role development of study design, data collection and interpretation, or writing of the final report. The corresponding author had full access all data and had final responsibility for submitting for publication.

Conflict of interests: None.

Data Sharing Agreement: Not applicable. No data sharing agreement was obtained for this study because data analysed was obtained from previous published studies where data sharing agreements are in place.

Ethical Approvals: Not applicable. No ethical approval was obtained for this study because data analysed was obtained from previous published studies where ethical approvals are in place.

Author contributions: CM and BG conceived the study and contributed to the design of the project. CM created the search strategy and BG, SWM, JP, AL, and CD contributed to subsequent iterations. CM, HF, EL, and TC conducted the search. CM and BG extracted and analysed the data. CM drafted the paper, BG, SWM, JP, AM, AL, and CD critically commented on the manuscript. All authors reviewed drafts and approved the final version

For peer review only

References

1. National population projections: 2018-based. Office for National Statistics. Availalble at https://www.ons.gov.uk/peoplepopulationandcommunity/populationandmigration/populationprojections/bulletins/nationalpopulationprojections/2018based [accessed 19 September 2021] [
2. Multimorbidity: a priority for global health research. The Academy of Medical Sciences. Available at https://acmedsci.ac.uk/policy/policy-projects/multimorbidity [accessed 18 April 2022] [
3. Johansson S, Rosengren A, Young K, Jennings E. Mortality and morbidity trends after the first year in survivors of acute myocardial infarction: a systematic review. BMC cardiovascular disorders. 2017;17(1):53.
4. Jones PB. Adult mental health disorders and their age at onset. British journal of psychiatry. 2013;202(s54):s5-s10.
5. Hanlon P, Nicholl BI, Jani BD, Lee D, McQueenie R, Mair FS. Frailty and pre-frailty in middle-aged and older adults and its association with multimorbidity and mortality: a prospective analysis of 493 737 UK Biobank participants. The Lancet Public health. 2018;3(7):e323-e32.
6. Williams JS, Egede LE. The Association Between Multimorbidity and Quality of Life, Health Status and Functional Disability. The American journal of the medical sciences. 2016;352(1):45-52.
7. Cassell A, Edwards D, Harshfield A, Rhodes K, Brimicombe J, Payne R, et al. The epidemiology of multimorbidity in primary care: a retrospective cohort study. The British journal of general practice : the journal of the Royal College of General Practitioners. 2018;68(669):e245.
8. Luben R, Hayat S, Wareham N, Pharoah PP, Khaw KT. Sociodemographic and lifestyle predictors of incident hospital admissions with multimorbidity in a general population, 1999-2019: the EPIC-Norfolk cohort. Bmj Open. 2020;10(9).
9. McCormack LA, Madlock-Brown C. Social Determinant of Health Documentation Trends and Their Association with Emergency Department Admissions. AMIA Annual Symposium proceedings AMIA Symposium. 2020;2020:823-32.
10. Huygen FJA. Family medicine : the medical life history of families / F.J.A. Huygen. London: Royal College of General Practitioners; 1990.
11. Longman JM, I Rolfe M, Passey MD, Heathcote KE, Ewald DP, Dunn T, et al. Frequent hospital admission of older people with chronic disease: A cross-sectional survey with telephone follow-up and data linkage. BMC Health Services Research. 2012;12(1).
12. Ridge A, Peterson GM, Kitsos A, Seidel BM, Anderson V, Nash R. Potentially Preventable Hospitalisations in rural Community-Dwelling Patients. Internal medicine journal. 2021.
13. Bu F, Philip K, Fancourt D. Social isolation and loneliness as risk factors for hospital admissions for respiratory disease among older adults. Thorax. 2020;75(7):597-9.
14. Luben R, Hayat S, Khawaja A, Wareham N, Pharoah PP, Khaw K-T. Residential area deprivation and risk of subsequent hospital admission in a British population: the EPIC-Norfolk cohort. BMJ open. 2019;9(12):e031251-e.
15. Mohnen SM, Schneider S, Droomers M. Neighborhood characteristics as determinants of healthcare utilization – a theoretical model. Health economics review. 2019;9(1):1-9.
16. Griffin E, McCarthy JP, Thomas F, Kingham S. New Zealand Healthline call data used to measure the effect of travel time on the use of the emergency department. Social science & medicine (1982). 2017;179:91-6.
17. PRISMA: Transparent Reporting of Systematic Reviews and Meta-Analyses. Aavailable at http://www.prisma-statement.org [accessed 7 September 2021].
18. Ingram E, Ledden S, Beardon S, Gomes M, Hogarth S, McDonald H, et al. Household and area-level social determinants of multimorbidity: a systematic review. Journal of epidemiology and community health (1979). 2021;75(3):232-41.
19. Ho IS-S, Azcoaga-Lorenzo A, Akbari A, Black C, Davies J, Hodgins P, et al. Examining variation in the measurement of multimorbidity in research: a systematic review of 566 studies. The Lancet Public Health. 2021;6(8):e587-e97.

For peer review only - http://bmjopen.bmj.com/site/about/guidelines.xhtml

20. Stafford M, Deeny SR, Dreyer K, Shand J. Multiple long-term conditions within households and use of health and social care: a retrospective cohort study. BJGP open. 2021;5(2):BJGPO.2020.0134.
21. A conceptual framework for action on the social determinants of health: social determinants of health discussion paper 2. World Health Organisation. Available at https://apps.who.int/iris/bitstream/handle/10665/44489/9789241500852_eng.pdf?sequence=1&isAllowed=y [accessed 28 September 2021] [
22. Innovation VH. Covidence systematic review software. Melbourne: Veritas Health Innovation VIC, 2016. [
23. The Newcastle-Ottowa Assessment Scale (NOAS) for assessing the quality of nonrandomised studies in meta-analyses. Available at http://www.ohri.ca/programs/clinical_epidemiology/oxford.asp [accessed 5 October 2021] [
24. Campbell M, McKenzie JE, Sowden A, Katikireddi SV, Brennan SE, Ellis S, et al. Synthesis without meta-analysis (SWiM) in systematic reviews: reporting guideline. BMJ. 2020;368:l6890-l.
25. Thomas JHJ. Cochrane Handbook for Systematic Reviews of Interventions. Cochrane Training. 2019, version 6.
26. Fisher KA, Griffith LE, Gruneir A, Upshur R, Perez R, Favotto L, et al. Effect of socio-demographic and health factors on the association between multimorbidity and acute care service use: population-based survey linked to health administrative data. Bmc Health Services Research. 2021;21(1).
27. Mbuya-Bienge C, Simard M, Gaulin M, Candas B, Sirois C. Does socio-economic status influence the effect of multimorbidity on the frequent use of ambulatory care services in a universal healthcare system? A population-based cohort study. Bmc Health Services Research. 2021;21(1).
28. Lu L, Zeng J, Zeng Z. What limits the utilization of health services among china labor force? analysis of inequalities in demographic, socio-economic and health status. International journal for equity in health. 2017;16(1):30-.
29. Pati S, Swain S, Hussain MA, Kadam S, Salisbury C. Prevalence, correlates, and outcomes of multimorbidity among patients attending primary care in Odisha, India. Annals of Family Medicine. 2015;13(5):446-50.
30. Chung RY, Mercer SW, Yip BHK, Chan SWC, Lai FTT, Wang HHX, et al. The association between types of regular primary care and hospitalization among people with and without multimorbidity: A household survey on 25,780 Chinese. Scientific Reports. 2016;6.
31. Orueta JF, García-Álvarez A, Alonso-Morán E, Vallejo-Torres L, Nuño-Solinis R. Socioeconomic variation in the burden of chronic conditions and health care provision - Analyzing administrative individual level data from the Basque Country, Spain. BMC Public Health. 2013;13(1).
32. Payne RA, Abel GA, Guthrie B, Mercer SW. The effect of physical multimorbidity, mental health conditions and socioeconomic deprivation on unplanned admissions to hospital: A retrospective cohort study. CMAJ. 2013;185(5):E221-E8.
33. Tomita A, Leyna GH, Kim HY, Moodley Y, Mpolya E, Mogeni P, et al. Patterns of multimorbidity and their association with hospitalisation: a population-based study of older adults in urban Tanzania. Age and Ageing. 2021;50(4):1349-60.
34. Wang HHX, Wang JJ, Lawson KD, Wong SYS, Wong MCS, Li FJ, et al. Relationships of multimorbidity and income with hospital admissions in 3 health care systems. Annals of Family Medicine. 2015;13(2):164-7.
35. Cassell A, Edwards D, Harshfield A, Rhodes K, Brimicombe J, Payne R, et al. The epidemiology of multimorbidity in primary care: A retrospective cohort study. British Journal of General Practice. 2018;68(669):e245-e51.
36. Identifying property and street information. Available at https://www.gov.uk/government/publications/open-standards-for-government/identifying-property-and-street-information [accessed 19 March 2022] [

For peer review only

For peer review only - http://bmjopen.bmj.com/site/about/guidelines.xhtml

37. Doheny M, Agerholm J, Orsini N, Schon P, Burstrom B. Socio-demographic differences in the frequent use of emergency department care by older persons: a population-based study in Stockholm County. Bmc Health Services Research. 2019;19.
38. Wray CM, Tang J, Lopez L, Hoggatt K, Keyhani S. Association of Social Determinants of Health and Their Cumulative Impact on Hospitalization Among a National Sample of Community-Dwelling US Adults. Journal of general internal medicine : JGIM. 2021.
39. Herm A, Anson JON, Poulain M. Living arrangements and marital status: a register-based study of survival of older adults in Belgium at the beginning of the 21st century. Ageing and society. 2016;36(10):2141-62.
40. Giannouchos TV, Kum H-C, Foster MJ, Ohsfeldt RL. Characteristics and predictors of adult frequent emergency department users in the United States: A systematic literature review. Journal of evaluation in clinical practice. 2019;25(3):420-33.
41. Gong CH, Kendig H, He X. Factors predicting health services use among older people in China: An analysis of the China Health and Retirement Longitudinal Study 2013. BMC health services research. 2016;16(1):63-.

For peer review only

Tables and Figures

Figure 1. Study selection, PRISMA diagram

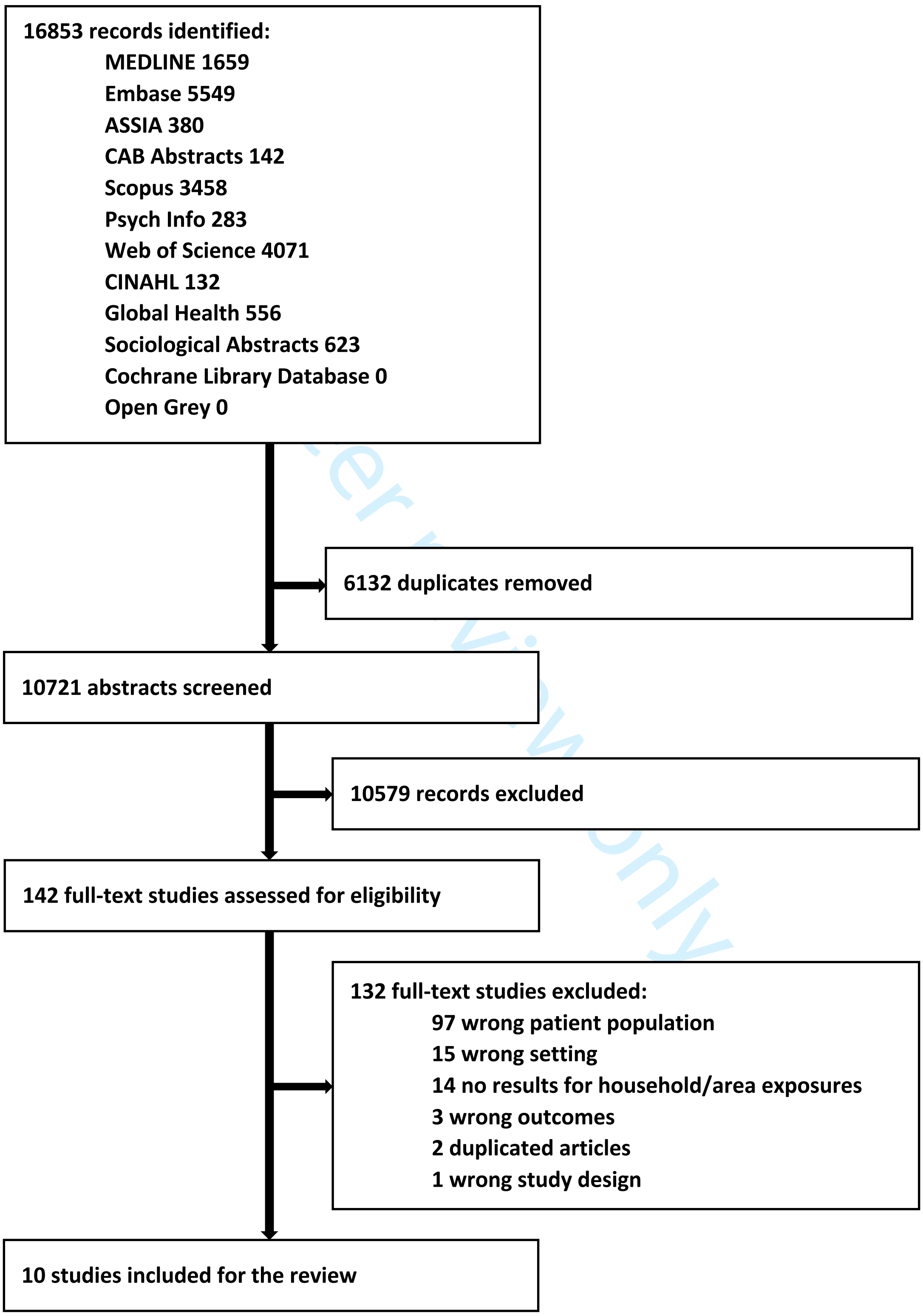

Supplementary File

Table S1. Database search strategies

**Database (number of articles)**	**Search strategy**
MedLine (1659)	(MULTIMORBIDITY/ or "multimorbidit*".mp. or "multi-morbidit*".mp. or "comorbidit*".mp. or "co-morbidit*".mp. or "polymorbidit*".mp. or "poly-morbidit*".mp. or"multicondition*".mp. or "multi-condition*".mp. or "multiple chronic condition*".mp. or "morbidity burden*".mp. or ((multiple or coexisting or co-existing or concurrent or con-current or comorbid* or co-morbid* or several*) adj2 (disease* or illness* or condition* or diagnosis* or morbid* or patholog*))) AND ("household*".mp. or "coresiden*".mp. or "co-residen*".mp. or ((household or family or domestic or residen*) adj2 (composition* or member* or characterist* or dweller*)) or "neighborhood*".mp. or "neighbourhood*".mp. or "small area*".mp. or "small-area*".mp. or "place".mp. or "communit*".mp. or "census tracts".mp. or "census block group".mp. or "zip code*".mp. or "post code*".mp. or "electoral ward*".mp. or "data zone*".mp. or "output area*".mp. or "lower super output area*".mp. or "municipalit*".mp. or "provinc*".mp. or SOCIOECONOMIC FACTORS/ or INCOME/ or "socioeconomic".mp. or "socio-economic".mp. or "social capital".mp. or "inequalit*".mp. or "disparit*".mp. or "income".mp. or "wealth".mp. or "financial problem*".mp. or "financial difficult*".mp. or "vocation*".mp. or "training opportunit*".mp. or "employment opportunit*".mp. or "occupation*".mp. or "opportunit*".mp. or "job opportunit*".mp. or "job insecurit".mp. or "education*" or "opportunit*".mp. or "education*".mp. or "achiev*".mp. or "education*".mp. or "quality".mp. or "fast-food".mp. or "fast food".mp. or "healthy food".mp. or "social determinant".mp. or "wider determinant".mp. or POVERTY AREAS/ or POVERTY/ or "neighbourhood disorder".mp. or "overcrowding".mp. or "public housing".mp. or "population density".mp. or "urban".mp. or "ghetto".mp. or "rural".mp. or "slum".mp. or "estate".mp. or "poverty area*".mp. or "physical environment*".mp. or "built environment*".mp. or "living standard*".mp. or "family structure".mp. or "family breakdown".mp. or "family disintegrat*".mp. or "single parent*".mp. or "housing tenure".mp. or "Townsend".mp. or "Carstairs".mp. or "Index of Multiple Deprivation".mp. or "Nam-Powers".mp. or "Hollingshead Index".mp. or "Breadline Britain Index".mp. or "inverse care law".mp. or "Singh ind*".mp. or "Yost ind*".mp. or "Kolak ind*".mp. or "social determinants of health".mp. or "neighbourhood archetype*".mp. or "segregation indic*".mp. or "ecological inversion".mp. or "ecological fallacy".mp. or "ecological bias".mp. or "depriv*".mp. or "impoverish*".mp. or "disadvantage*".mp. or "community cohesion".mp. or "community network".mp. or "community support".mp. or "transport* quality".mp. or "transport* services".mp. or "transport link*".mp. or "public transport*".mp. or "travel time".mp. or "community resource access*".mp. or "walkability".mp. or "public space".mp. or "green space".mp. or "open space".mp. or "recreation*".mp. or "leisure activit*".mp. or "food outlet*".mp. or "food environment".mp. or "air pollution".mp. or "particulate matter".mp. or "nitrogen dioxide".mp. or "sulphur dioxide".mp. or "sulfur dioxide".mp. or "carbon monoxide".mp. or "crime*".mp.) AND ("hospital admission*".mp. or "hospitalisation".mp. or "hospitalization".mp. or "emergency admission*".mp. or "unscheduled admission*".mp. or "unplanned admission*".mp. or "emergency department".mp. or "accident and emergency".mp.)<br>limit to (english language and yr="2000-Current")
EMBASE (5549)	("multimorbidit*".mp. or "multi-morbidit*".mp. or "comorbidit*".mp. or "co-morbidit*".mp. or "polymorbidit*".mp. or "poly-morbidit*".mp. or"multicondition*".mp. or "multi-condition*".mp. or "multiple chronic condition*".mp. or "morbidity burden*".mp. or "multiple long-term condition*".mp. or ((multiple or coexisting or co-existing or concurrent or con-current or comorbid* or co-morbid* or several*) adj2 (disease* or illness* or condition* or diagnosis* or morbid* or patholog*))) AND ("household*".mp. or "coresiden*".mp. or "co-residen*".mp. or ((household or family or domestic or residen*) adj2 (composition* or member* or characterist* or dweller*)) or "neighborhood*".mp. or "neighbourhood*".mp. or "small area*".mp. or "small-area*".mp. or "place".mp. or "communit*".mp. or "census tracts".mp. or "census block group".mp. or "zip code*".mp. or "post code*".mp. or "electoral ward*".mp. or "data zone*".mp. or "output area*".mp. or "lower super output area*".mp. or "municipalit*".mp. or "provinc*".mp. or "socioeconomic".mp. or "socio-economic".mp. or "social capital".mp. or "inequalit*".mp. or "disparit*".mp. or "income".mp. or "wealth".mp. or "financial problem*".mp. or "financial difficult*".mp. or "vocation*".mp. or "training opportunit*".mp. or "employment opportunit*".mp. or "occupation*".mp. or "opportunit*".mp. or "job opportunit*".mp. or "job insecurit".mp. or "education*" or "opportunit*".mp. or "education*".mp. or "achiev*".mp. or "education*".mp. or "quality".mp. or "fast-food".mp. or "fast food".mp. or "healthy food".mp. or "social determinant".mp. or "wider determinant".mp. or "neighbourhood disorder".mp. or "overcrowding".mp. or "public housing".mp. or "population density".mp. or "urban".mp. or "ghetto".mp. or "rural".mp. or "slum".mp. or "estate".mp. or "poverty area*".mp. or "physical environment*".mp. or "built environment*".mp. or "living standard*".mp. or "family structure".mp. or "family breakdown".mp. or "family disintegrat*".mp. or "single parent*".mp. or "housing tenure".mp. or "Townsend".mp. or "Carstairs".mp. or "Index of Multiple Deprivation".mp. or "Nam-Powers".mp. or "Hollingshead Index".mp. or "Breadline Britain Index".mp. or "inverse care law".mp. or "Singh ind*".mp. or "Yost ind*".mp. or "Kolak ind*".mp. or "social determinants of health".mp. or "neighbourhood archetype*".mp. or "segregation indic*".mp. or "ecological inversion".mp. or "ecological fallacy".mp. or "ecological bias".mp. or "depriv*".mp. or "impoverish*".mp. or "disadvantage*".mp. or "community cohesion".mp. or "community network".mp. or "community support".mp. or "transport* quality".mp. or "transport* services".mp. or "transport link*".mp. or "public transport*".mp. or "travel time".mp. or "community resource access*".mp. or "walkability".mp. or "public space".mp. or "green space".mp. or "open space".mp. or "recreation*".mp. or "leisure activit*".mp. or "food

	outlet*".mp. or "food environment".mp. or "air pollution".mp. or "particulate matter".mp. or "nitrogen dioxide".mp. or "sulphur dioxide".mp. or "sulfur dioxide".mp. or "carbon monoxide".mp. or "crime*".mp.) AND ("admission*".mp. or "hospital admission*".mp. or "hospitalisation".mp. or "hospitalization".mp. or "emergency admission*".mp. or "unscheduled admission*".mp. or "unplanned admission*".mp. or "emergency department".mp. or "accident and emergency".mp.)<br>limit to (english language and yr="2000-Current")
Global Health (556)	("multimorbidit*".mp. or "multi-morbidit*".mp. or "comorbidit*".mp. or "co-morbidit*".mp. or "polymorbidit*".mp. or "poly-morbidit*".mp. or"multicondition*".mp. or "multi-condition*".mp. or "multiple chronic condition*".mp. or "morbidity burden*".mp. or ((multiple or coexisting or co-existing or concurrent or con-current or comorbid* or co-morbid* or several*) adj2 (disease* or illness* or condition* or diagnosis* or morbid* or patholog*))) AND ("household*".mp. or "coresiden*".mp. or "co-residen*".mp. or ((household or family or domestic or residen*) adj2 (composition* or member* or characterist* or dweller*)) or "neighborhood*".mp. or "neighbourhood*".mp. or "small area*".mp. or "small-area*".mp. or "place".mp. or "communit*".mp. or "census tracts".mp. or "census block group".mp. or "zip code*".mp. or "post code*".mp. or "electoral ward*".mp. or "data zone*".mp. or "output area*".mp. or "lower super output area*".mp. or "municipalit*".mp. or "provinc*".mp. or "socioeconomic".mp. or "socio-economic".mp. or "social capital".mp. or "inequalit*".mp. or "disparit*".mp. or "income".mp. or "wealth".mp. or "financial problem*".mp. or "financial difficult*".mp. or "vocation*".mp. or "training opportunit*".mp. or "employment opportunit*".mp. or "occupation*".mp. or "opportunit*".mp. or "job opportunit*".mp. or "job insecurit".mp. or "education*" or "opportunit*".mp. or "education*".mp. or "achiev*".mp. or "education*".mp. or "quality".mp. or "fast-food".mp. or "fast food".mp. or "healthy food".mp. or "social determinant".mp. or "wider determinant".mp. or "neighbourhood disorder".mp. or "overcrowding".mp. or "public housing".mp. or "population density".mp. or "urban".mp. or "ghetto".mp. or "rural".mp. or "slum".mp. or "estate".mp. or "poverty area*".mp. or "physical environment*".mp. or "built environment*".mp. or "living standard*".mp. or "family structure".mp. or "family breakdown".mp. or "family disintegrat*".mp. or "single parent*".mp. or "housing tenure".mp. or "Townsend".mp. or "Carstairs".mp. or "Index of Multiple Deprivation".mp. or "Nam-Powers".mp. or "Hollingshead Index".mp. or "Breadline Britain Index".mp. or "inverse care law".mp. or "Singh ind*".mp. or "Yost ind*".mp. or "Kolak ind*".mp. or "social determinants of health".mp. or "neighbourhood archetype*".mp. or "segregation indic*".mp. or "ecological inversion".mp. or "ecological fallacy".mp. or "ecological bias".mp. or "depriv*".mp. or "impoverish*".mp. or "disadvantage*".mp. or "community cohesion".mp. or "community network".mp. or "community support".mp. or "transport* quality".mp. or "transport* services".mp. or "transport link*".mp. or "public transport*".mp. or "travel time".mp. or "community resource access*".mp. or "walkability".mp. or "public space".mp. or "green space".mp. or "open space".mp. or "recreation*".mp. or "leisure activit*".mp. or "food outlet*".mp. or "food environment".mp. or "air pollution".mp. or "particulate matter".mp. or "nitrogen dioxide".mp. or "sulphur dioxide".mp. or "sulfur dioxide".mp. or "carbon monoxide".mp. or "crime*".mp.) AND ("hospital admission*".mp. or "hospitalisation".mp. or "hospitalization".mp. or "emergency admission*".mp. or "unscheduled admission*".mp. or "unplanned admission*".mp. or "emergency department".mp. or "accident and emergency".mp.)<br>limit to (english language and yr="2000-Current")
PsychInfo (283)	("multimorbidit*".mp. or "multi-morbidit*".mp. or "comorbidit*".mp. or "co-morbidit*".mp. or "polymorbidit*".mp. or "poly-morbidit*".mp. or"multicondition*".mp. or "multi-condition*".mp. or "multiple chronic condition*".mp. or "morbidity burden*".mp. or ((multiple or coexisting or co-existing or concurrent or con-current or comorbid* or co-morbid* or several*) adj2 (disease* or illness* or condition* or diagnosis* or morbid* or patholog*))) AND ("household*".mp. or "coresiden*".mp. or "co-residen*".mp. or ((household or family or domestic or residen*) adj2 (composition* or member* or characterist* or dweller*)) or "neighborhood*".mp. or "neighbourhood*".mp. or "small area*".mp. or "small-area*".mp. or "place".mp. or "communit*".mp. or "census tracts".mp. or "census block group".mp. or "zip code*".mp. or "post code*".mp. or "electoral ward*".mp. or "data zone*".mp. or "output area*".mp. or "lower super output area*".mp. or "municipalit*".mp. or "provinc*".mp. or "socioeconomic".mp. or "socio-economic".mp. or "social capital".mp. or "inequalit*".mp. or "disparit*".mp. or "income".mp. or "wealth".mp. or "financial problem*".mp. or "financial difficult*".mp. or "vocation*".mp. or "training opportunit*".mp. or "employment opportunit*".mp. or "occupation*".mp. or "opportunit*".mp. or "job opportunit*".mp. or "job insecurit".mp. or "education*" or "opportunit*".mp. or "education*".mp. or "achiev*".mp. or "education*".mp. or "quality".mp. or "fast-food".mp. or "fast food".mp. or "healthy food".mp. or "social determinant".mp. or "wider determinant".mp. or "neighbourhood disorder".mp. or "overcrowding".mp. or "public housing".mp. or "population density".mp. or "urban".mp. or "ghetto".mp. or "rural".mp. or "slum".mp. or "estate".mp. or "poverty area*".mp. or "physical environment*".mp. or "built environment*".mp. or "living standard*".mp. or "family structure".mp. or "family breakdown".mp. or "family disintegrat*".mp. or "single parent*".mp. or "housing tenure".mp. or "Townsend".mp. or "Carstairs".mp. or "Index of Multiple Deprivation".mp. or "Nam-Powers".mp. or "Hollingshead Index".mp. or "Breadline Britain Index".mp. or "inverse care law".mp. or "Singh ind*".mp. or "Yost ind*".mp. or "Kolak ind*".mp. or "social determinants of health".mp. or "neighbourhood archetype*".mp. or "segregation indic*".mp. or "ecological inversion".mp. or "ecological fallacy".mp. or "ecological bias".mp. or "depriv*".mp. or "impoverish*".mp. or "disadvantage*".mp. or "community cohesion".mp. or "community network".mp. or "community support".mp. or "transport* quality".mp. or "transport* services".mp. or "transport link*".mp. or "public transport*".mp. or "travel time".mp. or "community resource access*".mp. or "walkability".mp. or "public space".mp. or "green space".mp. or "open space".mp. or "recreation*".mp. or "leisure activit*".mp. or "food outlet*".mp. or "food environment".mp. or "air pollution".mp. or "particulate matter".mp. or "nitrogen dioxide".mp. or "sulphur dioxide".mp. or "sulfur dioxide".mp. or "carbon monoxide".mp. or "crime*".mp.) AND

For peer review only

	("hospital admission*".mp. or "hospitalisation".mp. or "hospitalization".mp. or "emergency admission*".mp. or "unscheduled admission*".mp. or "unplanned admission*".mp. or "emergency department".mp. or "accident and emergency".mp.)<br>limit to (english language and yr="2000-Current")
ASSIA (380)	("multimorbidit*" or "multi-morbidit*" or "comorbidit*" or "co-morbidit*" or "polymorbidit*" or "poly-morbidit*" or "multicondition*" or "multi-condition*" or "multiple chronic condition*" or "morbidity burden*" or ((multiple or coexisting or co-existing or concurrent or con-current or comorbid* or co-morbid* or several*) adj2 (disease* or illness* or condition* or diagnosis* or morbid* or patholog*))) AND ("household*" or "coresiden*" or "co-residen*" or ((household or family or domestic or residen*) adj2 (composition* or member* or characterist* or dweller*)) or "neighborhood*" or "neighbourhood*" or "small area*" or "small-area*" or "place" or "communit*" or "census tracts" or "census block group" or "zip code*" or "post code*" or "electoral ward*" or "data zone*" or "output area*" or "lower super output area*" or "municipalit*" or "provinc*" or "socioeconomic" or "socio-economic" or "social capital" or "inequalit*" or "disparit*" or "income" or "wealth" or "financial problem*" or "financial difficult*" or "vocation*" or "training opportunit*" or "employment opportunit*" or "occupation* opportunit*" or "job opportunit*" or "job insecurit" or "education* opportunit*" or "education* achiev*" or "education* quality" or "fast-food" or "fast food" or "healthy food" or "social determinant" or "wider determinant" or "neighbourhood disorder" or "overcrowding" or "public housing" or "population density" or "urban" or "ghetto" or "rural" or "slum" or "estate" or "poverty area*" or "physical environment*" or "built environment*" or "living standard*" or "family structure" or "family breakdown" or "family disintegrat*" or "single parent*" or "housing tenure" or "Townsend" or "Carstairs" or "Index of Multiple Deprivation" or "Nam-Powers" or "Hollingshead Index" or "Breadline Britain Index" or "inverse care law" or "Singh ind*" or "Yost ind*" or "Kolak ind*" or "social determinants of health" or "neighbourhood archetype*" or "segregation indic*" or "ecological inversion" or "ecological fallacy" or "ecological bias" or "depriv*" or "impoverish*" or "disadvantage*" or "community cohesion" or "community network" or "community support" or "transport* quality" or "transport* services" or "transport link*" or "public transport*" or "travel time" or "community resource access*" or "walkability" or "public space" or "green space" or "open space" or "recreation*" or "leisure activit*" or "food outlet*" or "food environment" or "air pollution" or "particulate matter" or "nitrogen dioxide" or "sulphur dioxide" or "sulfur dioxide" or "carbon monoxide" or "crime*") AND ("hospital admission*" or "hospitalisation" or "hospitalization" or "emergency admission*" or "unscheduled admission*" or "unplanned admission*" or "emergency department" or "accident and emergency")<br>Publication date: after 01 January 2000
CAB Abstracts (142)	("multimorbidit*" or "multi-morbidit*" or "comorbidit*" or "co-morbidit*" or "polymorbidit*" or "poly-morbidit*" or "multicondition*" or "multi-condition*" or "multiple chronic condition*" or "morbidity burden*" or ((multiple or coexisting or co-existing or concurrent or con-current or comorbid* or co-morbid* or several*) adj2 (disease* or illness* or condition* or diagnosis* or morbid* or patholog*))) AND ("household*" or "coresiden*" or "co-residen*" or ((household or family or domestic or residen*) adj2 (composition* or member* or characterist* or dweller*)) or "neighborhood*" or "neighbourhood*" or "small area*" or "small-area*" or "place" or "communit*" or "census tracts" or "census block group" or "zip code*" or "post code*" or "electoral ward*" or "data zone*" or "output area*" or "lower super output area*" or "municipalit*" or "provinc*" or "socioeconomic" or "socio-economic" or "social capital" or "inequalit*" or "disparit*" or "income" or "wealth" or "financial problem*" or "financial difficult*" or "vocation*" or "training opportunit*" or "employment opportunit*" or "occupation* opportunit*" or "job opportunit*" or "job insecurit" or "education* opportunit*" or "education* achiev*" or "education* quality" or "fast-food" or "fast food" or "healthy food" or "social determinant" or "wider determinant" or "neighbourhood disorder" or "overcrowding" or "public housing" or "population density" or "urban" or "ghetto" or "rural" or "slum" or "estate" or "poverty area*" or "physical environment*" or "built environment*" or "living standard*" or "family structure" or "family breakdown" or "family disintegrat*" or "single parent*" or "housing tenure" or "Townsend" or "Carstairs" or "Index of Multiple Deprivation" or "Nam-Powers" or "Hollingshead Index" or "Breadline Britain Index" or "inverse care law" or "Singh ind*" or "Yost ind*" or "Kolak ind*" or "social determinants of health" or "neighbourhood archetype*" or "segregation indic*" or "ecological inversion" or "ecological fallacy" or "ecological bias" or "depriv*" or "impoverish*" or "disadvantage*" or "community cohesion" or "community network" or "community support" or "transport* quality" or "transport* services" or "transport link*" or "public transport*" or "travel time" or "community resource access*" or "walkability" or "public space" or "green space" or "open space" or "recreation*" or "leisure activit*" or "food outlet*" or "food environment" or "air pollution" or "particulate matter" or "nitrogen dioxide" or "sulphur dioxide" or "sulfur dioxide" or "carbon monoxide" or "crime*") AND ("hospital admission*" or "hospitalisation" or "hospitalization" or "emergency admission*" or "unscheduled admission*" or "unplanned admission*" or "emergency department" or "accident and emergency")<br>Publication date: after 01 January 2000
Web of Science (4071)	("multimorbidit*" or "multi-morbidit*" or "comorbidit*" or "co-morbidit*" or "polymorbidit*" or "poly-morbidit*" or "multicondition*" or "multi-condition*" or "multiple chronic condition*" or "morbidity burden*" or ((multiple or coexisting or co-existing or concurrent or con-current or comorbid* or co-morbid* or several*) adj2 (disease* or illness* or condition* or diagnosis* or morbid* or patholog*))) AND ("household*" or "coresiden*" or "co-residen*" or ((household or family or domestic or residen*) adj2 (composition* or member* or characterist* or dweller*)) or "neighborhood*" or "neighbourhood*" or "small area*" or "small-area*" or "place" or "communit*" or "census tracts" or "census block group" or "zip code*" or "post code*" or "electoral ward*" or "data zone*" or

For peer review only

	"output area*" or "lower super output area*" or "municipalit*" or "provinc*" or "socioeconomic" or "socio-economic" or "social capital" or "inequalit*" or "disparit*" or "income" or "wealth" or "financial problem*" or "financial difficult*" or "vocation*" or "training opportunit*" or "employment opportunit*" or "occupation* opportunit*" or "job opportunit*" or "job insecurit" or "education* opportunit*" or "education* achiev*" or "education* quality" or "fast-food" or "fast food" or "healthy food" or "social determinant" or "wider determinant" or "neighbourhood disorder" or "overcrowding" or "public housing" or "population density" or "urban" or "ghetto" or "rural" or "slum" or "estate" or "poverty area*" or "physical environment*" or "built environment*" or "living standard*" or "family structure" or "family breakdown" or "family disintegrat*" or "single parent*" or "housing tenure" or "Townsend" or "Carstairs" or "Index of Multiple Deprivation" or "Nam-Powers" or "Hollingshead Index" or "Breadline Britain Index" or "inverse care law" or "Singh ind*" or "Yost ind*" or "Kolak ind*" or "social determinants of health" or "neighbourhood archetype*" or "segregation indic*" or "ecological inversion" or "ecological fallacy" or "ecological bias" or "depriv*" or "impoverish*" or "disadvantage*" or "community cohesion" or "community network" or "community support" or "transport* quality" or "transport* services" or "transport link*" or "public transport*" or "travel time" or "community resource access*" or "walkability" or "public space" or "green space" or "open space" or "recreation*" or "leisure activit*" or "food outlet*" or "food environment" or "air pollution" or "particulate matter" or "nitrogen dioxide" or "sulphur dioxide" or "sulfur dioxide" or "carbon monoxide" or "crime*") AND ("hospital admission*" or "hospitalisation" or "hospitalization" or "emergency admission*" or "unscheduled admission*" or "unplanned admission*" or "emergency department" or "accident and emergency")<br>Language: English<br>Publication date: after 01 January 2000
Scopus (3458)	("multimorbidit*" or "multi-morbidit*" or "comorbidit*" or "co-morbidit*" or "polymorbidit*" or "poly-morbidit*" or "multicondition*" or "multi-condition*" or "multiple chronic condition*" or "morbidity burden*" or ((multiple or coexisting or co-existing or concurrent or con-current or comorbid* or co-morbid* or several*) adj2 (disease* or illness* or condition* or diagnosis* or morbid* or patholog*))) AND ("household*" or "coresiden*" or "co-residen*" or ((household or family or domestic or residen*) adj2 (composition* or member* or characterist* or dweller*)) or "neighborhood*" or "neighbourhood*" or "small area*" or "small-area*" or "place" or "communit*" or "census tracts" or "census block group" or "zip code*" or "post code*" or "electoral ward*" or "data zone*" or "output area*" or "lower super output area*" or "municipalit*" or "provinc*" or "socioeconomic" or "socio-economic" or "social capital" or "inequalit*" or "disparit*" or "income" or "wealth" or "financial problem*" or "financial difficult*" or "vocation*" or "training opportunit*" or "employment opportunit*" or "occupation* opportunit*" or "job opportunit*" or "job insecurit" or "education* opportunit*" or "education* achiev*" or "education* quality" or "fast-food" or "fast food" or "healthy food" or "social determinant" or "wider determinant" or "neighbourhood disorder" or "overcrowding" or "public housing" or "population density" or "urban" or "ghetto" or "rural" or "slum" or "estate" or "poverty area*" or "physical environment*" or "built environment*" or "living standard*" or "family structure" or "family breakdown" or "family disintegrat*" or "single parent*" or "housing tenure" or "Townsend" or "Carstairs" or "Index of Multiple Deprivation" or "Nam-Powers" or "Hollingshead Index" or "Breadline Britain Index" or "inverse care law" or "Singh ind*" or "Yost ind*" or "Kolak ind*" or "social determinants of health" or "neighbourhood archetype*" or "segregation indic*" or "ecological inversion" or "ecological fallacy" or "ecological bias" or "depriv*" or "impoverish*" or "disadvantage*" or "community cohesion" or "community network" or "community support" or "transport* quality" or "transport* services" or "transport link*" or "public transport*" or "travel time" or "community resource access*" or "walkability" or "public space" or "green space" or "open space" or "recreation*" or "leisure activit*" or "food outlet*" or "food environment" or "air pollution" or "particulate matter" or "nitrogen dioxide" or "sulphur dioxide" or "sulfur dioxide" or "carbon monoxide" or "crime*") AND ("hospital admission*" or "hospitalisation" or "hospitalization" or "emergency admission*" or "unscheduled admission*" or "unplanned admission*" or "emergency department" or "accident and emergency")<br>Publication date: after 01 January 2000
CINAHL (132)	("multimorbidit*" or "multi-morbidit*" or "comorbidit*" or "co-morbidit*" or "polymorbidit*" or "poly-morbidit*" or "multicondition*" or "multi-condition*" or "multiple chronic condition*" or "morbidity burden*" or ((multiple or coexisting or co-existing or concurrent or con-current or comorbid* or co-morbid* or several*) adj2 (disease* or illness* or condition* or diagnosis* or morbid* or patholog*))) AND ("household*" or "coresiden*" or "co-residen*" or ((household or family or domestic or residen*) adj2 (composition* or member* or characterist* or dweller*)) or "neighborhood*" or "neighbourhood*" or "small area*" or "small-area*" or "place" or "communit*" or "census tracts" or "census block group" or "zip code*" or "post code*" or "electoral ward*" or "data zone*" or "output area*" or "lower super output area*" or "municipalit*" or "provinc*" or "socioeconomic" or "socio-economic" or "social capital" or "inequalit*" or "disparit*" or "income" or "wealth" or "financial problem*" or "financial difficult*" or "vocation*" or "training opportunit*" or "employment opportunit*" or "occupation* opportunit*" or "job opportunit*" or "job insecurit" or "education* opportunit*" or "education* achiev*" or "education* quality" or "fast-food" or "fast food" or "healthy food" or "social determinant" or "wider determinant" or "neighbourhood disorder" or "overcrowding" or "public housing" or "population density" or "urban" or "ghetto" or "rural" or "slum" or "estate" or "poverty area*" or "physical environment*" or "built environment*" or "living standard*" or "family structure" or "family breakdown" or "family disintegrat*" or "single parent*" or "housing tenure" or "Townsend" or "Carstairs" or "Index of Multiple Deprivation" or "Nam-Powers" or "Hollingshead Index" or "Breadline Britain Index" or "inverse care law" or "Singh ind*" or "Yost ind*" or "Kolak ind*" or "social determinants of health" or "neighbourhood archetype*" or "segregation indic*" or "ecological inversion" or "ecological fallacy" or "ecological bias" or "depriv*" or "impoverish*" or "disadvantage*" or "community cohesion" or "community network" or

For peer review only

	"community support" or "transport* quality" or "transport* services" or "transport link*" or "public transport*" or "travel time" or "community resource access*" or "walkability" or "public space" or "green space" or "open space" or "recreation*" or "leisure activit*" or "food outlet*" or "food environment" or "air pollution" or "particulate matter" or "nitrogen dioxide" or "sulphur dioxide" or "sulfur dioxide" or "carbon monoxide" or "crime*") AND ("hospital admission*" or "hospitalisation" or "hospitalization" or "emergency admission*" or "unscheduled admission*" or "unplanned admission*" or "emergency department" or "accident and emergency")<br>Publication date: after 01 January 2000
Sociological Abstracts (623)	("multimorbidit*" or "multi-morbidit*" or "comorbidit*" or "co-morbidit*" or "polymorbidit*" or "poly-morbidit*" or "multicondition*" or "multi-condition*" or "multiple chronic condition*" or "morbidity burden*" or ((multiple or coexisting or co-existing or concurrent or con-current or comorbid* or co-morbid* or several*) adj2 (disease* or illness* or condition* or diagnosis* or morbid* or patholog*))) AND ("household*" or "coresiden*" or "co-residen*" or ((household or family or domestic or residen*) adj2 (composition* or member* or characterist* or dweller*)) or "neighborhood*" or "neighbourhood*" or "small area*" or "small-area*" or "place" or "communit*" or "census tracts" or "census block group" or "zip code*" or "post code*" or "electoral ward*" or "data zone*" or "output area*" or "lower super output area*" or "municipalit*" or "provinc*" or "socioeconomic" or "socio-economic" or "social capital" or "inequalit*" or "disparit*" or "income" or "wealth" or "financial problem*" or "financial difficult*" or "vocation*" or "training opportunit*" or "employment opportunit*" or "occupation* opportunit*" or "job opportunit*" or "job insecurit" or "education* opportunit*" or "education* achiev*" or "education* quality" or "fast-food" or "fast food" or "healthy food" or "social determinant" or "wider determinant" or "neighbourhood disorder" or "overcrowding" or "public housing" or "population density" or "urban" or "ghetto" or "rural" or "slum" or "estate" or "poverty area*" or "physical environment*" or "built environment*" or "living standard*" or "family structure" or "family breakdown" or "family disintegrat*" or "single parent*" or "housing tenure" or "Townsend" or "Carstairs" or "Index of Multiple Deprivation" or "Nam-Powers" or "Hollingshead Index" or "Breadline Britain Index" or "inverse care law" or "Singh ind*" or "Yost ind*" or "Kolak ind*" or "social determinants of health" or "neighbourhood archetype*" or "segregation indic*" or "ecological inversion" or "ecological fallacy" or "ecological bias" or "depriv*" or "impoverish*" or "disadvantage*" or "community cohesion" or "community network" or "community support" or "transport* quality" or "transport* services" or "transport link*" or "public transport*" or "travel time" or "community resource access*" or "walkability" or "public space" or "green space" or "open space" or "recreation*" or "leisure activit*" or "food outlet*" or "food environment" or "air pollution" or "particulate matter" or "nitrogen dioxide" or "sulphur dioxide" or "sulfur dioxide" or "carbon monoxide" or "crime*") AND ("hospital admission*" or "hospitalisation" or "hospitalization" or "emergency admission*" or "unscheduled admission*" or "unplanned admission*" or "emergency department" or "accident and emergency")<br>Publication date: after 01 January 2000
Cochrane (0)	("multimorbidit*" or "multi-morbidit*" or "comorbidit*" or "co-morbidit*" or "polymorbidit*" or "poly-morbidit*" or "multicondition*" or "multi-condition*" or "multiple chronic condition*" or "morbidity burden*" or ((multiple or coexisting or co-existing or concurrent or con-current or comorbid* or co-morbid* or several*) adj2 (disease* or illness* or condition* or diagnosis* or morbid* or patholog*)))
OpenGrey (0)	("multimorbidit*" or "multi-morbidit*" or "comorbidit*" or "co-morbidit*" or "polymorbidit*" or "poly-morbidit*" or "multicondition*" or "multi-condition*" or "multiple chronic condition*" or "morbidity burden*" or ((multiple or coexisting or co-existing or concurrent or con-current or comorbid* or co-morbid* or several*) adj2 (disease* or illness* or condition* or diagnosis* or morbid* or patholog*)))

For peer review only

Table S2. Study methodological quality ratings: Newcastle-Ottowa Assessment (NOAS), adapted for cross-sectional studies

Source	Selection				Comparability based on design and analysis	Outcome		Total
	Representativeness of the sample	Sample size	Non-respondents	Ascertainment of exposure		Assessment of outcome	Statistical test	
Lu et al (2017)	Half*	*	*	Half*	*	Half*	*	6.5 (Medium)
Fisher et al (2021)	Half*	*	*	Half*	**	*	*	7 (High)
Mbuya-Bienge et al (2021)	*	*	*	*	*	*	Half*	6.5 (Medium)
Oureta et al (2013)	*	*	Half*	*	*	*	Half*	6.5 (Medium)
Pati et al (20 15)	Half*	Half*	Half*	Half*	*	Half*	*	4.5(Medium)
Payne et al (2013)	*	*	*	*	**	*	*	8 (High)
Stafford et al (2020)	Half*	Half*	half*	*	*	*	*	5.5 (Medium)
Tomita et al (2021)	*	*	*	*	**	Half*	*	7.5 (High)
Wang et al	Half*	*	Half*	Half*	**	Half*	*	6 (Medium)

Methodological score: high = 7-8, medium = 4-6, low = 0-3

For peer review only

Box S1. Risk of bias Newcastle-Ottowa Assessment (NOAS), adapted for this study and application to examination of cross-sectional studies

A study can be awarded a maximum of one point for each numbered item within the Selection and Exposure categories. A maximum of two points can be given for the Comparability category.

Selection (maximum 4)

1. Representativeness of the sample

a. Truly representative of the average in the target population (random sample or whole population) *

b. Somewhat representative of the average in the target population (purposive sampling of representative healthcare sector areas or evidence that the sample is representative of the source population) [half]*

c. Selected group of users

d. No description of the sampling strategy

2. Sample size

a. Justified and satisfactory *

b. Adequately powered to detect a difference (at least 10 events per variable in multivariate analyses)*

c. Not justified

3. Non-respondents

a. Comparability between respondents and non-respondents characteristics is established, and the response rate is satisfactory (>60%)*

b. The response rate is unsatisfactory, or the comparability between respondents and non-respondents is unsatisfactory

c. No description of the response rate or the characteristics of the responders and non-responders

4. Risk factor exposure assessment

a. Assessment of exposure including type and duration from electronic health record or administrative database*

b. Assessment of exposure from questionnaire *

c. No description

Comparability (maximum 2)

5. The subjects in different outcome groups are comparable, based on the study design or analysis. Confounding factors are adjusted for

a. Study controls for multimorbidity and no multimorbidity, including age and gender adjustment**

b. Study controls for multimorbidity and no multimorbidity, without additional variable adjustment*

c. Study does not control for multimorbidity

Outcome (maximum 2)

6. Assessment of the outcome (emergency department or unscheduled hospital admission)

a. Electronic health record or health payment record account of healthcare use *

b. Reported healthcare use from questionnaire or no described data source [half]*

7. Statistical test

a. The statistical test used to analyse the data is clearly described and appropriate, and the measurement of the association is presented as either an OR, CI and P value or a beta coefficient, SE, and P value*

b. The statistical test is not appropriate, not described, or incomplete

For peer review only

Checkilsts

1. Preferred Reporting Items for Systematic Reviews and Meta-Analyses (PRISMA) 2020 Checklist

Section and Topic	Item #	Checklist item	Location where item is reported
**TITLE**			
Title	1	Identify the report as a systematic review.	Methods – paragraph 1<br>(Page 4)
**ABSTRACT**			
Abstract	2	See the PRISMA 2020 for Abstracts checklist.	See checklist below
**INTRODUCTION**			
Rationale	3	Describe the rationale for the review in the context of existing knowledge.	Introduction – paragraphs 1-3<br>(Page 4)
Objectives	4	Provide an explicit statement of the objective(s) or question(s) the review addresses.	Introduction – paragraphs 2-3<br>(Page 4)
**METHODS**			
Eligibility criteria	5	Specify the inclusion and exclusion criteria for the review and how studies were grouped for the syntheses.	Methods – paragraph 3, Table 1<br>(Page 5)
Information sources	6	Specify all databases, registers, websites, organisations, reference lists and other sources searched or consulted to identify studies. Specify the date when each source was last searched or consulted.	Methods – paragraph 1<br>(Page 5)
Search strategy	7	Present the full search strategies for all databases, registers and websites, including any filters and limits used.	Table S1
Selection process	8	Specify the methods used to decide whether a study met the inclusion criteria of the review, including how many reviewers screened each record and each report retrieved, whether they worked independently, and if applicable, details of automation tools used in the process.	Methods – paragraph 4<br>(Page 4)
Data collection process	9	Specify the methods used to collect data from reports, including how many reviewers collected data from each report, whether they worked independently, any processes for obtaining or confirming data from study investigators, and if applicable, details of automation tools used in the process.	Methods – paragraph 6<br>(Page 5)
Data items	10a	List and define all outcomes for which data were sought. Specify whether all results that were compatible with each outcome domain in each study were sought (e.g. for all measures, time points, analyses), and if not, the methods used to decide which results to collect.	Methods – paragraph 6<br>(Page 5)
	10b	List and define all other variables for which data were sought (e.g. participant and intervention characteristics, funding sources). Describe any assumptions made about any missing or unclear information.	Methods – paragraph 6<br>(Page 5)
Study risk of bias assessment	11	Specify the methods used to assess risk of bias in the included studies, including details of the tool(s) used, how many reviewers assessed each study and whether they worked independently, and if applicable, details of automation tools used in the process.	Methods – paragraph 5<br>(Page 5)
Effect measures	12	Specify for each outcome the effect measure(s) (e.g. risk ratio, mean difference) used in the synthesis or presentation of results.	Methods – paragraph 7

For peer review only

Section and Topic	Item #	Checklist item	Location where item is reported
			(Page 6)
Synthesis methods	13a	Describe the processes used to decide which studies were eligible for each synthesis (e.g. tabulating the study intervention characteristics and comparing against the planned groups for each synthesis (item #5)).	Methods – paragraph 7 (Page 6)
	13b	Describe any methods required to prepare the data for presentation or synthesis, such as handling of missing summary statistics, or data conversions.	Methods – paragraph 7 (Page 6)
	13c	Describe any methods used to tabulate or visually display results of individual studies and syntheses.	Methods – paragraph 7 (Page 6)
	13d	Describe any methods used to synthesize results and provide a rationale for the choice(s). If meta-analysis was performed, describe the model(s), method(s) to identify the presence and extent of statistical heterogeneity, and software package(s) used.	Methods – paragraph 7 (Page 5)
	13e	Describe any methods used to explore possible causes of heterogeneity among study results (e.g. subgroup analysis, meta-regression).	Methods – paragraph 7 (Page 5)
	13f	Describe any sensitivity analyses conducted to assess robustness of the synthesized results.	Not applicable
Reporting bias assessment	14	Describe any methods used to assess risk of bias due to missing results in a synthesis (arising from reporting biases).	Methods – paragraph 5 (Page 5)
Certainty assessment	15	Describe any methods used to assess certainty (or confidence) in the body of evidence for an outcome.	Methods – paragraph 7 (Page 5)
**RESULTS**			
Study selection	16a	Describe the results of the search and selection process, from the number of records identified in the search to the number of studies included in the review, ideally using a flow diagram.	Results – paragraph 1 (Page 6)
	16b	Cite studies that might appear to meet the inclusion criteria, but which were excluded, and explain why they were excluded.	Introduction – paragraph 2 (Page 4)
Study characteristics	17	Cite each included study and present its characteristics.	Results – paragraphs 2-4 and 7-12 (Pages 6-8), Table 2
Risk of bias in studies	18	Present assessments of risk of bias for each included study.	Results – paragraph 8 (Page 7), Table S2
Results of individual studies	19	For all outcomes, present, for each study: (a) summary statistics for each group (where appropriate) and (b) an effect estimate and its precision (e.g. confidence/credible interval), ideally using structured tables or plots.	Results – paragraphs 3-16 (Page 6-10), Table 3
Results of syntheses	20a	For each synthesis, briefly summarise the characteristics and risk of bias among contributing studies.	Results – paragraphs 8-16 (Page 7-10).

For peer review only

Section and Topic	Item #	Checklist item	Location where item is reported
	20b	Present results of all statistical syntheses conducted. If meta-analysis was done, present for each the summary estimate and its precision (e.g. confidence/credible interval) and measures of statistical heterogeneity. If comparing groups, describe the direction of the effect.	Results – paragraphs 9-16 (Page 8-10)
	20c	Present results of all investigations of possible causes of heterogeneity among study results.	Results – paragraphs 9-16 (Page 8-10)
	20d	Present results of all sensitivity analyses conducted to assess the robustness of the synthesized results.	Not applicable
Reporting biases	21	Present assessments of risk of bias due to missing results (arising from reporting biases) for each synthesis assessed.	Table S2
Certainty of evidence	22	Present assessments of certainty (or confidence) in the body of evidence for each outcome assessed.	Results – paragraphs 9-16 (Page 8-10)
**DISCUSSION**			
Discussion	23a	Provide a general interpretation of the results in the context of other evidence.	Discussion - paragraph 1 (Page 10)
	23b	Discuss any limitations of the evidence included in the review.	Discussion – paragraph 2 (Page 10)
	23c	Discuss any limitations of the review processes used.	Discussion – paragraph 2 (Page 10)
	23d	Discuss implications of the results for practice, policy, and future research.	Discussion – paragraph 6 (Page 11-12)
**OTHER INFORMATION**			
Registration and protocol	24a	Provide registration information for the review, including register name and registration number, or state that the review was not registered.	Methods – paragraph 1 (Page 4)
	24b	Indicate where the review protocol can be accessed, or state that a protocol was not prepared.	Methods – paragraph 1 (Page 4)
	24c	Describe and explain any amendments to information provided at registration or in the protocol.	Not applicable
Support	25	Describe sources of financial or non-financial support for the review, and the role of the funders or sponsors in the review.	Declarations (Page 13)
Competing interests	26	Declare any competing interests of review authors.	Declarations (Page 13)
Availability of data, code and other materials	27	Report which of the following are publicly available and where they can be found: template data collection forms; data extracted from included studies; data used for all analyses; analytic code; any other materials used in the review.	Stable 2, Table 3, upplementary file

From: Page MJ, McKenzie JE, Bossuyt PM, Boutron I, Hoffmann TC, Mulrow CD, et al. The PRISMA 2020 statement: an updated guideline for reporting systematic reviews. BMJ 2021;372:n71. doi: 10.1136/bmj.n71

For peer review only

2. PRISMA 2020 for Abstracts Checklist

Section and Topic	Item #	Checklist item	Reported (Yes/No)
**TITLE**			
Title	1	Identify the report as a systematic review.	Background
**BACKGROUND**			
Objectives	2	Provide an explicit statement of the main objective(s) or question(s) the review addresses.	Methods
**METHODS**			
Eligibility criteria	3	Specify the inclusion and exclusion criteria for the review.	Methods
Information sources	4	Specify the information sources (e.g. databases, registers) used to identify studies and the date when each was last searched.	Methods (and main paper)
Risk of bias	5	Specify the methods used to assess risk of bias in the included studies.	No (main paper)
Synthesis of results	6	Specify the methods used to present and synthesise results.	Methods
**RESULTS**			
Included studies	7	Give the total number of included studies and participants and summarise relevant characteristics of studies.	No (main paper)
Synthesis of results	8	Present results for main outcomes, preferably indicating the number of included studies and participants for each. If meta-analysis was done, report the summary estimate and confidence/credible interval. If comparing groups, indicate the direction of the effect (i.e. which group is favoured).	Results
**DISCUSSION**			
Limitations of evidence	9	Provide a brief summary of the limitations of the evidence included in the review (e.g. study risk of bias, inconsistency and imprecision).	No (main paper)
Interpretation	10	Provide a general interpretation of the results and important implications.	Results
**OTHER**			
Funding	11	Specify the primary source of funding for the review.	No (main paper)
Registration	12	Provide the register name and registration number.	Methods

From: Page MJ, McKenzie JE, Bossuyt PM, Boutron I, Hoffmann TC, Mulrow CD, et al. The PRISMA 2020 statement: an updated guideline for reporting systematic reviews. BMJ 2021;372:n71. doi: 10.1136/bmj.n71
For more information, visit: http://www.prisma-statement

For peer review only

3. Synthesis Without Meta-analysis (SWiM) reporting items

**SWiM is intended to complement and be used as an extension to PRISMA**			
**SWiM reporting item**	**Item description**	**Page in manuscript where item is reported**	**Other***
Methods			
1 Grouping studies for synthesis	1a) Provide a description of, and rationale for, the groups used in the synthesis (e.g., groupings of populations, interventions, outcomes, study design)	Methods – paragraph 7 (Pages 5-6)	
	1b) Detail and provide rationale for any changes made subsequent to the protocol in the groups used in the synthesis		Not applicable
2 Describe the standardised metric and transformation methods used	Describe the standardised metric for each outcome. Explain why the metric(s) was chosen, and describe any methods used to transform the intervention effects, as reported in the study, to the standardised metric, citing any methodological guidance consulted	Results – paragraph 5-16 (Pages 7-10)	
3 Describe the synthesis methods	Describe and justify the methods used to synthesise the effects for each outcome when it was not possible to undertake a meta-analysis of effect estimates	Methods – paragraphs 5 and 7 (Pages 5-6)	
4 Criteria used to prioritise results for summary and synthesis	Where applicable, provide the criteria used, with supporting justification, to select the particular studies, or a particular study, for the main synthesis or to draw conclusions from the synthesis (e.g., based on study design, risk of bias assessments, directness in relation to the review question)	Results – paragraph 5-16 (Pages 7-10)	
5 Investigation of heterogeneity in reported effects	State the method(s) used to examine heterogeneity in reported effects when it was not possible to undertake a meta-analysis of effect estimates and its extensions to investigate heterogeneity	Results – paragraph 5-16 (Pages 7-10)	
6 Certainty of evidence	Describe the methods used to assess certainty of the synthesis findings		Not applicable
7 Data presentation methods	Describe the graphical and tabular methods used to present the effects (e.g., tables, forest plots, harvest plots).<br>Specify key study characteristics (e.g., study design, risk of bias) used to order the studies, in the text and any tables or graphs, clearly referencing the studies included	Table 2, Table 3,	
Results			
8 Reporting results	For each comparison and outcome, provide a description of the synthesised findings, and the certainty of the findings. Describe the result in language that is consistent with the question the synthesis addresses, and indicate which studies contribute to the synthesis	Results – paragraph 5-16 (Pages 7-10)	
Discussion			
9 Limitations of the synthesis	Report the limitations of the synthesis methods used and/or the groupings used in the synthesis, and how these affect the conclusions that can be drawn in relation to the original review question	Discussion – paragraph 2 (Page 10)	

For peer review only

Household and area determinants of emergency department attendance and hospitalisation in people with multimorbidity: a systematic review

Journal:	*BMJ Open*
Manuscript ID	bmjopen-2022-063441.R2
Article Type:	Original research
Date Submitted by the Author:	30-Aug-2022
Complete List of Authors:	MacRae, Clare; The University of Edinburgh Usher Institute of Population Health Sciences and Informatics,<br>Fisken, Harry; The University of Edinburgh Edinburgh Medical School<br>Lawrence, Edward; Ninewells Hospital and Medical School<br>Connor, Thomas; The University of Edinburgh Edinburgh Medical School<br>Pearce, Jamie; University of Edinburgh Institute of Geography, Institute of Geography<br>Marshall, Alan; The University of Edinburgh Social Policy, Social Policy<br>Lawson, Andrew; Medical University of South Carolina, Department of Public Health Sciences<br>Dibben, Chris; University of Edinburgh Institute of Geography<br>Mercer, Stewart; The University of Edinburgh, The Usher Institute of Population Health Sciences and Informatics<br>Guthrie, Bruce; The University of Edinburgh Usher Institute of Population Health Sciences and Informatics
**Primary Subject Heading**:	Public health
Secondary Subject Heading:	Epidemiology
Keywords:	EPIDEMIOLOGY, PUBLIC HEALTH, STATISTICS & RESEARCH METHODS

SCHOLARONE™
Manuscripts

I, the Submitting Author has the right to grant and does grant on behalf of all authors of the Work (as defined in the below author licence), an exclusive licence and/or a non-exclusive licence for contributions from authors who are: i) UK Crown employees; ii) where BMJ has agreed a CC-BY licence shall apply, and/or iii) in accordance with the terms applicable for US Federal Government officers or employees acting as part of their official duties; on a worldwide, perpetual, irrevocable, royalty-free basis to BMJ Publishing Group Ltd ("BMJ") its licensees and where the relevant Journal is co-owned by BMJ to the co-owners of the Journal, to publish the Work in this journal and any other BMJ products and to exploit all rights, as set out in our licence.

The Submitting Author accepts and understands that any supply made under these terms is made by BMJ to the Submitting Author unless you are acting as an employee on behalf of your employer or a postgraduate student of an affiliated institution which is paying any applicable article publishing charge ("APC") for Open Access articles. Where the Submitting Author wishes to make the Work available on an Open Access basis (and intends to pay the relevant APC), the terms of reuse of such Open Access shall be governed by a Creative Commons licence – details of these licences and which Creative Commons licence will apply to this Work are set out in our licence referred to above.

Other than as permitted in any relevant BMJ Author's Self Archiving Policies, I confirm this Work has not been accepted for publication elsewhere, is not being considered for publication elsewhere and does not duplicate material already published. I confirm all authors consent to publication of this Work and authorise the granting of this licence.

For peer review only

Household and area determinants of emergency department attendance and hospitalisation in people with multimorbidity: a systematic review

Authors: Clare MacRae[1], Harry Fisken[2], Edward Lawrence[3], Thomas Connor[2], Jamie Pearce[4], Alan Marshall[5], Andrew Lawson[1, 6], Chris Dibben[4], Stewart W Mercer[1], Bruce Guthrie[1]

[1]The University of Edinburgh Usher Institute of Population Health Sciences and Informatics University of Edinburgh, EH8 9AG Edinburgh, UK EH8 9AG

[2] The University of Edinburgh Medical School Edinburgh, Edinburgh, UK

[3] Ninewells Hospital and Medical School Dundee, Dundee, UK

[4] University of Edinburgh Institute of Geography, Institute of Geography Edinburgh, Edinburgh, UK

[5] The University of Edinburgh Social Policy, Social Policy Edinburgh, Edinburgh, UK

[6] Medical University of South Carolina, Department of Public Health Sciences Charleston, USA

Corresponding author: Dr Clare MacRae, Usher Institute, College of Medicine and Veterinary Medicine, University of Edinburgh, EH8 9AG, clare.macrae@ed.ac.uk

PROSPERO registration number: CRD42021283515

For peer review only - http://bmjopen.bmj.com/site/about/guidelines.xhtml

Abstract

Objectives: Multimorbidity is one of the greatest challenges facing healthcare internationally. Emergency department (ED) attendance and hospitalisation rates are higher in people with multimorbidity, but most research focuses on associations with individual characteristics, ignoring household or area mediators of service use.

Design: Systematic review reported using the synthesis without meta-analysis framework.

Data sources: Twelve electronic databases (January 1st, 2000, to September 21st, 2021): MEDLINE/OVID, Embase, Global Health, PsycINFO, ASSIA, CAB Abstracts, Science Citation Index Expanded/ISI Web of Science, Scopus, Cumulative Index to Nursing and Allied Health Literature [CINAHL], Sociological Abstracts, the Cochrane Library, and OpenGrey.

Eligibility criteria: Adults aged ≥16 years, with multimorbidity. Exposure(s) were household and/or area determinants of health. Outcomes were ED attendance and/or hospitalisation. The literature search was limited to publications in English.

Data extraction and synthesis: Independent double screening of titles and abstracts to select relevant full-text studies. Methodological quality was assessed using an adaptation of the Newcastle-Ottawa Quality Assessment Scale tool. Given high study heterogeneity, narrative synthesis was performed.

Results: After de-duplication, 10,721 titles and abstracts were screened, and 142 full-text articles reviewed, of which ten were eligible for inclusion. In people with multimorbidity, household food insecurity was associated with hospitalisation (OR 1.58 [95%CI 1.06-2.36] in concordant multimorbidity). People with multimorbidity living in the most versus least deprived areas attended ED more frequently (8.9% [95%CI 8.6-9.1] in most, versus 6.3% [95%CI 6.1-6.6] in least), had higher rates of hospitalisation (26% in most, versus 22% in least), and higher probability of hospitalisation (6.4% [95%CI 5.8-7.2] in most, versus 4.2% [95%CI 3.8-4.7] in least). There was non-conclusive evidence that household income is associated with ED attendance and hospitalisation. No statistically significant relationships were found between marital status, living with others with multimorbidity, or rurality and ED attendance or hospitalisation.

Conclusions: There is some evidence that household and area context mediate associations of multimorbidity with ED attendance and hospitalisation, but firm conclusions are constrained by the small number of studies published and study design heterogeneity. Further research is required on large population samples using robust analytical methods.

For peer review only - http://bmjopen.bmj.com/site/about/guidelines.xhtml

PROSPERO registration number: CRD42021283515.

Word count: 343

For peer review only - http://bmjopen.bmj.com/site/about/guidelines.xhtml

Strengths and limitations of this study

- Comprehensive literature search of twelve electronic databases to examine associations between household or area context and hospital attendance, specifically in people with multimorbidity
- Reporting as per Preferred Reporting Items for Systematic Reviews and Meta-Analyses and Synthesis Without Meta-analysis guidelines
- Limited evidence available in current literature with a small number of studies meeting eligibility criteria
- Heterogeneity in multimorbidity definition and measurement, and study exposures, outcomes, and analysis methods, precluded meta-analysis

For peer review only

Introduction

Multimorbidity is usually defined as the presence of two or more long-term physical or mental health conditions and is one of the greatest challenges facing healthcare internationally. It is becoming more common globally because of population ageing,[1, 2] improved survival after acute illnesses such as myocardial infarction,[3] and changing patterns of mental health in younger people.[4] There are large independent associations between multimorbidity and adverse outcomes including frailty,[5] reduced functional health status,[6] and hospital attendance.[7] People with multimorbidity are at nearly three times the risk of hospitalisation compared to those without.[7]

Research usually compares different groups of people with multimorbidity, for example defined by their individual characteristics, such as age, sex, and lifestyle factors.[8, 9] However, for this important patient group, social, and environmental contexts are rarely examined in depth. People live in a social context, where attitudes and practices can be influenced by those around them, for example households have been described as “a living and developing unit of interdependent members, sharing common internal and external conditions and interactions”.[10] Research examining general adult populations (not stratified by multimorbidity) has found that people who live in a household with fewer financial or food resources,[11, 12] and single person households,[13] are at higher risk of emergency department (ED) attendance and hospitalisation. The areas in which people live can influence health - for example, populations not stratified by multimorbidity living in urban areas, and in areas with low socioeconomic position (SEP), with associated additional health burdens, are similarly at risk.[14] Households and areas are therefore an important unit of measurement when considering patterns of disease and healthcare use.[15] To date, there is a paucity of synthesis of studies examining these data in people with multimorbidity. Additionally, factors such as geographical proximity to services are known to be associated with likelihood of ED attendance.[16] Consideration of variation in how services themselves operate, including supply-side factors such as relative accessibility of primary care versus ED care, are important.

Understanding whether and how context influences outcomes is needed to ensure that the call by the Academy of Medical Sciences for research into “how to organise healthcare systems to better manage patients with multimorbidity” is based on appropriate understanding of both the individual and their social and environmental context.[2] The aim of this review is to examine, in people with multimorbidity, associations of household and area contextual exposures with emergency department (ED) attendance and hospitalisation.

For peer review only - http://bmjopen.bmj.com/site/about/guidelines.xhtml

Methods

Method development and reporting of findings of the systematic review were based on the PRISMA-P 2020 checklist.[17] The protocol was registered with PROSPERO CRD42021283515.

Eligibility criteria and inclusion

Eligible studies included populations of adults aged 16 years and over, from the general population, who had been assessed for, and had, the presence of multimorbidity or multiple long-term conditions (LTCs), defined according to the Academy of Medical Sciences core definition of multimorbidity as 'two or more chronic conditions' (Table 1).[2] Study exposure(s) examined were one or more household and/or area determinant of health, including built environment, and socioeconomic determinants of health (Table 1).[18] Study outcomes were ED attendance and/or planned or unplanned hospitalisation.

For peer review only

Table 1. Study inclusion and exclusion criteria

	Inclusion	Exclusion
**Population**	Adult participants from the general population residing in the community, aged 16 years and older and assessed for the presence of multiple LTCs (multimorbidity)	Participants initially selected based on the presence of index diseases, including any study examining comorbidity<br>Studies exclusively examining children aged 15 years and younger<br>Participants within hospital settings, or examination of readmission where the dominator is previously hospitalised participants
**Exposure**	≥ 1 household or area-level socioeconomic determinant of health (SDoH) in alignment with the World Health Organisation (WHO) Commission on SDoH (CSDH) Framework[18]<br>Household: One residential unit, characteristics can include reference to the built environment, or the occupants living within<br>Area: Geographical area within which a person lives including all area sizes larger than household unit	Individual SDoH only (e.g., ethnicity)<br>Study exposure(s) are direct "causes" of ill-health, such as health behaviours (e.g., smoking)
**Comparator**	Study reports comparator group(s) for SDoH exposures (e.g., prevalence of hospitalisation admission in lowest versus highest household income)	Study does not report a comparator group for SDoH exposure(s)
**Outcome**	Prevalence or incidence studies examining emergency department use and hospitalisation (defined as a planned or unplanned overnight admission)	Studies not examining emergency department use or hospitalisation
**Study design**	Peer-reviewed studies of quantitative research designs (cross-sectional and longitudinal)	Systematic reviews, meta-analyses, clinical trials, and qualitative research

For peer review only - http://bmjopen.bmj.com/site/about/guidelines.xhtml

Search strategy

Twelve electronic databases (MEDLINE/OVID, Embase, Global Health, PsycINFO, ASSIA, CAB Abstracts, Science Citation Index Expanded/ISI Web of Science, Scopus, Cumulative Index to Nursing and Allied Health Literature [CINAHL], Sociological Abstracts, the Cochrane Library, and OpenGrey), and reference lists, were searched for full text articles meeting eligibility criteria, published between 1st January 2000, and 21st September 2021, which was the date the final searches took place. Searches were limited to articles written in English. An empirical approach to deriving the search terms was followed using a test set of three critical papers, one examining the household and area determinants of multimorbidity,[19] a second examining the definition and operationalisation of the term multimorbidity,[20] and a third meeting the study inclusion and exclusion criteria.[21] Key search terms, derived through reference to medical subject heading terms in test papers, iterated within MedLine to maximise sensitivity and specificity for relevant articles, were divided into three sets pertaining to multimorbidity, contextual exposures, and healthcare use (Table S1).

Following removal of duplicate records, independent double screening for relevance was performed for all titles and abstracts, using Covidence software,[22] and relevant full-text studies were selected (Figure 1). Conflicts were resolved through discussion between screeners (CM, HF, EL, TC) at each stage of screening and eligibility of all included studies was verified by a fifth author (BG).

Quality assessment

The Newcastle-Ottawa Quality Assessment Scale (NOAS) tool[23] was adapted (Box S1), and study quality screening for each paper was performed independently by two of the researchers (CM plus one of HF, EL, TC), with consensus achieved through discussion.

Data synthesis

CM performed data extraction using a custom spreadsheet to record study design, location, population (age, definition of multimorbidity), exposures (household and/or area), and outcome measures (ED attendance and/or hospitalisation). If data were missing or unclear, we contacted corresponding authors for clarification who provided additional data, and information regarding study methodology and analyses.

For peer review only - http://bmjopen.bmj.com/site/about/guidelines.xhtml

Study methodologies, exposures, outcomes, and effect measures were diverse and heterogenous, and narrative synthesis was therefore performed according to Synthesis Without Meta-analysis (SWiM)[24] and PRISMA guidelines.[17] Studies were grouped according to exposure examined, to ensure meaningful presentation of reviewed evidence.[25] Presentation of results using a standardised metric was not possible given the heterogeneity of outcome measures. Due to the small number of included studies, all study results were reported with quantitative figures given for statistically significant findings. Narrative comparison of heterogeneity between studies examining each exposure was performed by reviewing results within each group, comparing methodologies, exposures, and outcomes. Patients or the public were not involved in the design, conduct, reporting, or dissemination plans of this research.

Patient and Public Involvement

Patients or the public were not involved in the design, conduct, reporting, or dissemination plans of this research.

Results

The systematic search identified 16,853 articles. After removal of 6,132 duplicates, 10,721 titles and abstracts were screened, of which 10,579 were excluded at the first screening stage. Full text-screening of 142 articles identified 10 studies meeting eligibility criteria. Screening of reference lists yielded no additional studies eligible for inclusion (Figure 1).

Study characteristics

Two studies were conducted in North America,[26, 27] three in Asia,[28-30] three in Europe,[21, 31, 32] one in Africa, [33] and one study used multiple sites[34] (Table 2). All studies used cross-sectional data; two used data derived from existing cohort studies.[26, 27] Study sample size ranged from 1670 to 5316830 (median 27689 participants, interquartile range [IQR] 20689-216633). Seven studies reporting regression model outcomes,[21, 26, 28-30, 33] used smaller populations (1670-162464 participants, median 24642, IQR 15387-66474), than the two studies reporting rates of hospital attendance (2262698-5316830, median 3789764, IQR 3026231-4553297).[27, 31] Each included study examined a specific age-group, which were ≥15 years old (one study),[30] 15-65 years old (one study),[28] ≥18 years old (two studies),[27, 29] ≥20 years old (two studies),[32, 34] ≥40 years old (one study),[33] ≥50 years old (one study),[21] and ≥65 years old (two studies).[26, 31]

For peer review only - http://bmjopen.bmj.com/site/about/guidelines.xhtml

Table 2. Study characteristics.

Author	Study purpose	Study design	Data source a	Sample size	Setting and date	Target population	Multimorbidity definition	Household exposure	Area exposure	Primary outcome measure(s)[a]
Chung et al (2016),[30] Hong Kong	Examine factors associated with hospitalisation	Cross-sectional	Hong Kong Government household survey	25780	General population, 2011	≥ 15 yrs	≥ 2 LTCSs out of 45 LTCs, mental and physical	Household income and marital status	NA	12-month hospitalisation[b]
Fisher et al (2021),[26] Canada	Examine socio-demographic and health factors associated with acute care	Cross-sectional	Canadian Community Health Survey; administrative health data	28361	General population, pooled from multiple cycles 2005-2012	65 - 85 yrs	≥ 2 LTCSs out of 12 LTCs, physical and mental	Household income	NA	12-month ED attendance and hospitalisation[b]
Lu et al (2017),[28] China	Examine inequalities limiting utilisation of health services	Cross-sectional	China Labour Force Dynamic Survey	23505	Adult population in employment, 2014	15-65 yrs	≥ 2 LTCSs out of 10 LTCs, physical only	Marital status	NA	12-month hospitalisation[b]
Mbuya-Bienge et al (2021),[27] Canada	Examine SEP and frequency of use of healthcare	Cross-sectional	Quebec Integrated Chronic Disease System	5316830	General population, 2011-2012	≥18 yrs	≥ 2 LTCSs out of 31 LTCs, physical and mental	NA	Area SEP	12-month ED attendance
Orueta et al (2013),[31] Spain	Examine costs of multimorbidity by SEP	Cross-sectional	Routine administrative health data	2262698	General population, 2011	≥65 yrs	≥ 2 LTCSs out of 52 LTCs, physical and mental	NA	Area SEP	12-month hospitalisation[b]
Pati et al (20 15),[29] India	Examine relationship between multimorbidity and health care utilisation	Cross-sectional	Multi-stage random sampling patient interviews	1670	General population, 2013-2014	≥18 yrs	≥ 2 LTCSs out of 22 LTCs, physical and mental	Marital status	Rurality	12-month hospital in-patient or out-patient hospital use
Payne et al (2013),[32] Scotland,	Examine association between SEP and	Cross-sectional	Scottish Practice Team	180815	General population, 2006	≥18 yrs	≥ 2 LTCSs out of 52 LTCs,	NA	Area SEP	12-month emergency or potentially

For peer review only - http://bmjopen.bmj.com/site/about/guidelines.xhtml

China, Hong Kong	unplanned hospitalisation		Information dataset				physical and mental			preventable hospitalisation
Stafford et al (2020),[21] England	Examine association between co-resident multimorbidity status and hospitalisation	Cross-sectional	Barking and Dagenham (B&D) health data, Clinical Practice Research Datalink (CPRD)	9222 (B&D) and 10528 (CPRD)	General population, 2016-2018	≥ 50 yrs living in 2-person households	≥ 2 LTCSs out of 16 LTCs, physical and mental	Multimorbidity status of other household member	NA	Non-elective hospitalisation
Tomita et al (2021), [33] Tanzania	Examine association between SEP and hospitalisation	Cross-sectional	Dar es Salaam Health Demographic Surveillance System	2299	General population, 2017-2018	≥ 40 yrs	≥ 2 LTCSs out of 8 LTCs (concordant and discordant)[c], physical and mental	Marital status<br><br>Household food insecurity	NA	12-month hospitalisation[b]
Wang et at (2015),[34] Scotland, China, Hong Kong	Examine association between household income with hospitalisation	Cross-sectional	Scotland: Health Survey<br>China: Routine administrative data<br>Hong Kong: Household Survey	36921<br><br>162464<br><br>29187	General population, 2008<br><br>2011<br><br>2011	≥ 40 yrs	≥ 2 LTCSs out of 31 LTCs, physical and menta	Household income	NA	12-month hospitalisation[b]

[a] All outcomes are dichotomous (attendance or no attendance) rather than counts

[b] No differentiation planned/unplanned

[c] Concordant multimorbidity: 1 domain of mental health/non-communicable/communicable health. Discordant multimorbidity: ≥ domains.

For peer review only - http://bmjopen.bmj.com/site/about/guidelines.xhtml

Multimorbidity was defined as the presence of two or more LTCs by all studies, with the number of LTCs included in multimorbidity measures ranging from 8 to 52 (median 26.5, IQR 13-41.5). Nine studies included both physical and mental LTCs,[21, 26, 27, 29-35] and one study only included physical LTCs.[28] Various approaches were taken to defining the list of LTCs included, ranging from no included rationale by Lu et al[28] and Stafford et al,[21] adaptation of previous studies examining multimorbidity by Oureta et al,[31] to including LTCs included in the Charlson and Elixhauser indexes by Mbuya-Bienge et al.[27] Tomita et al[33] examined both concordant and discordant multimorbidity, fitting separate logistic regression models for each. In this study, discordant multimorbidity was defined as the occurrence of LTCs in ≥ two domains in mental health, non-communicable, and communicable disease.

The 10 included studies examined six unique exposures, where an exposure was defined as any household or area characteristic associated with ED attendance or hospitalisation. Seven studies examined household,[21, 26, 28-30, 33, 34] and four studies examined area exposures.[27, 29, 31, 32] One study, by Pati et al,[29] examined both household and area exposures.

Household exposures examined were household income (three studies),[26, 30, 34] multimorbidity status of other household members (one study),[21] household food insecurity (one study),[33] and marital status (four studies).[28-30, 33] Data on household income were derived through household surveys in all included studies.[26, 30, 34] Household food insecurity was defined using a cross-cultural measure, where survey participants were asked how often there was no food in their household, and how often household members went to sleep hungry or did not eat for a day.[33] Multimorbidity status of other household members was determined through linkage of local government and health provider administrative data with the unique property reference number,[21] a unique identifier for every addressable location in the United Kingdom.[36] Marital status was the most examined household exposure, but studies varied in how they examined this. Pati et al[29] classified participants as currently married and currently not married, and Lu et al[28] defined groups as married and single/divorced. Tomita et al[33] and Chung et al[30] divided participants into three groups comparing those who are currently married with never married, or separated/divorced/widowed. Stafford et al[21] examined multimorbidity status of household the other household resident in two-person households, with no reference to relationship status.

Area exposures examined were area SEP (three studies),[27, 31, 32] and rurality (one study).[29] All studies examining area SEP used a similar approach to deriving their SEP measure, using quintiles of a score derived from census tract deprivation indexes, based on rates of unemployment, educational attainment, and type of employment. Rurality was examined as a dichotomous exposure of rural versus urban, derived from population size and density, and proportion of workers.

For peer review only - http://bmjopen.bmj.com/site/about/guidelines.xhtml

Reported outcomes were any ED attendance (two studies),[26, 27] any hospitalisation (whether planned or unplanned, and some studies did not differentiate whether hospitalisations were planned or unplanned) (six studies),[26, 28, 30, 31, 33, 34] any inpatient or outpatient hospital attendance (one study),[29] any ED attendance or unplanned hospitalisation (one study),[21] and any unplanned or emergency hospitalisation or unplanned or emergency potentially avoidable hospitalisation (one study).[32] No studies presented results stratified by the number of attendances in the study period.

Methodological quality of studies varied across studies and was rated high in three studies,[26, 32, 33] and medium in six[21, 27-29, 31, 34] (Table S2). Potential bias related to reporting of hospital attendance outcomes, where studies used either questionnaire based self-reported outcomes,[28, 29, 33, 34] or electronic health records and health payment systems,[21, 26, 27, 31] therefore differences in accuracy and reporting could exist between these studies. Use of statistical reporting varied between studies, with six studies performing regression models with different model outcomes, including odds ratios (ORs),[21, 26, 28, 33] incidence rate ratios (IRRs),[29] event probability,[34] as well as studies examining unadjusted rates of hospital attendance.[27, 31]

Household income

There was inconclusive evidence of a relationship between household income and hospital use. Chung et al,[30] a medium quality study from Hong Kong, examined the relative risk (RR) of hospitalisation for households with different levels of income. Analyses were compared to household income of <$10K and RRs were close to 1.00 for all income strata, with all 95%CIs crossing 1.00 apart (from income of $10-19K, RR1.66, 95%CI 1.002-1.356). Fisher et al,[26] a Canadian study rated as high quality, examined associations between household income and service use in people with multimorbidity, with separate regression models fitted to sub-groups defined by age-group (65-74 and 75-84 years), sex, multimorbidity (categorised as 2-3 and ≥4 LTCs), and outcomes (ED attendance and hospitalisation) (Table 3). In people with multimorbidity, ORs for ED attendance in people with middle (Canadian $30-$79.9K) and lower (<$30K) household income, versus high (>$80K), were almost all >1 (15 out of 16). However, 12 (out of 16) had wide confidence intervals crossing 1, which is likely to reflect small numbers in each stratum. There was some evidence of a dose-response relationship (ORs for low versus high income were always larger than ORs for medium versus high income), and estimated ORs were all small to moderate in size (maximum likelihood in men aged 65-74 with ≥4 LTCs was OR 2.74, 95C%I 1.12-6.66 for low versus high household income). In models with hospitalisation as outcome, the ORs for middle and lower household income, versus high, were all >1, however only two (out of 16) ORs had 95%CIs not crossing 1, and a similar dose-response relationship between degree of income and hospitalisation was seen.

For peer review only - http://bmjopen.bmj.com/site/about/guidelines.xhtml

Table 3. Household exposure study results

Study exposure	Study	Outcome	Methodological quality rating	Covariates	Result metric	Key Results	Association at 95% for ORs, RRs, and IRRs*
Household income	Fisher et al[26] Canada	ED attendance and any hospital use	High	Stratified by age and number of LTCs	ORs and 95%CIs	Highly stratified sample with small group sizes<br>6 of 32 models showed statistically significant relationship between increased likelihood of ED attendance/any hospital use with lower household income	✓/-
	Chung et al[30] China	Hospitalisation	Medium	No details provided	Relative risk (RR) and 95%CIs	Highest RR of hospitalisation in households with income ≥$50,000 (RR 1.193 [0.916–1.553]) versus income <$10,000 (reference RR 1.000)	-
	Wang et al[34] Scotland, Hong Kong, China	Hospitalisation	Medium	Stratified by number of LTC and sex	Predicted probability (P) and 95%CIs	Scotland: Higher probability of hospitalisation in lowest (18.7% [95%CI 18.3-19.1]) versus highest (11.1% [95%CI 10.7-11.4]) household income<br>China (no medical insurance): Lower probability of hospital admission in lowest (8.2% [95%CI 7.9-8.5]) versus highest (13.1% [95%CI 12.2-14.0]) household income	NA
Co-resident multimorbidity	Stafford et al[21] England	ED attendance	Medium	Age, sex, SEP	ORs and 95%CIs	No difference in ED attendance between people with household co-resident with versus without multimorbidity (OR 1.08 [95%CI 0.95-1.23])	-
Household food insecurity	Tomita et al[33] Tanzania	Hospitalisation	High	Age, sex, marital status, education	ORs and 95%CIs	Increased likelihood of hospitalisation in severe versus little-to-no household food insecurity (OR 1.58 [95%CI 1.06-2.36])	✓
Household marital status	Tomita et al[33] Tanzania	Hospitalisation	High	Age, sex, education	ORs and 95%CIs	No difference in hospitalisation between currently married versus	-

						never married (OR 1.43 [95%CI 0.62-3.28]), or separated/divorced (OR 1.24 [95%CI 0.86-1.78])	
	Chung et al[30] China	Hospitalisation	Medium	No details provided	Relative risk (RR) and 95%CIs	No difference in RR of hospitalisation in widowed (RR 1.058 [95%CI 0.791-1.416], or in married (RR0.917 [95%CI 0.706-1.191]) versus single (reference RR 1.000)	-
	Lu et al[28] China	Hospitalisation	Medium	No details provided	ORs and 95%CIs	No difference in hospitalisation between married or single/divorced (OR 1.04 [95%CI 0.54-2.02])	-
	Pati et al[29] India	Inpatient and outpatient hospital use	Medium	Age, sex, ethnicity, education, SEP, rurality	IRRs and 95%CIs	No difference in inpatient or outpatient hospital use between currently married versus currently not married (IRR 1.17 [95%CI 0.85-1.61])	-

* "NA" study outcomes were not ORs or IRRs, "✓" studies where an association at 95% significance was found, "-" studies where no association at 95% significance was found, "✓/-" studies where results were mixed.

For peer review only - http://bmjopen.bmj.com/site/about/guidelines.xhtml

Wang et al,[34] a study rated as medium quality, found that in people with multimorbidity, lower household income was associated with a higher probability of hospitalisation in Scotland, and public hospitalisation in Hong Kong. A dose-response relationship was seen where probability of admission rose as the number of LTCs increased, for example, probability for hospitalisation was higher in people with ≥4 LTCs (probability 30.7%, 95%CI 30.3-31.7) than people with 2 LTCs (probability 18.85%, 95%CI 18.4-19.2) in the lowest household income group in Scotland. However, a reversed relationship, lower household income associated with lower probability of hospitalisation, was seen in China and in private hospitals in Hong Kong. For example, people with lowest household income had lowest probability of hospitalisation (24.7%, 95%CI 23.4-26.1) versus people with highest household income (36.1% [95%CI 33.6-38.6]), in people with ≥4 LTCs with no medical insurance in China.

Household co-resident multimorbidity status

An English study by Stafford et al,[21] examined the multimorbidity status of the other household resident in two-person households. In this study rated as medium quality, no significant difference between living with someone who had multimorbidity, versus someone who did not have multimorbidity, was found in association with ED attendance (OR 1.08 [95%CI 0.95-1.23]).

Household hunger

A statistically significant relationship between severe household hunger and hospitalisation was found in a Tanzanian study by Tomita et al,[33] rated as high quality. This relationship was found in both concordant (severe versus none-to-moderate household hunger OR 1.58 [95%CI 1.06-2.36]) and discordant (severe versus none-to-moderate household hunger OR 1.54 [95%CI 1.04-2.28]) multimorbidity.

Marital status

No significant association between marital status and any outcome was found in the four studies examining this. Chung et al[30] examined relative RR of hospitalisation depending on marital status, and there were no statistically significant results with all RR being close to 1.00 with wide 95%CIs crossing 1.00. Lu et al,[28] a Chinese study rated as medium quality, found a small but not statistically significant relationship between being married and hospitalisation (married versus unmarried OR 1.04 [95%CI 0.54-2.02]). Pati et al,[29] a study from India rated as medium quality, found a larger but not statistically significant relationship in the opposite direction between marital status and any inpatient or outpatient hospital attendance (unmarried versus married incidence rate ratio [IRR] 1.17 [95%CI 0.85-1.61]). Similarly, Tomita et al[33] found a small but not statistically significant association between not being married and hospitalisation in people with concordant multimorbidity (never married versus married OR 1.43 [95%CI 0.62-3.28]; divorced/separated vs married OR 1.24 [95%CI 0.86-1.7]) and in people with

For peer review only - http://bmjopen.bmj.com/site/about/guidelines.xhtml

discordant multimorbidity (never married versus married OR 1.44 [95%CI 0.63-3.28]; divorced/separated versus married OR 1.27 [95%CI 0.83-1.85]) in Tanzania.

Socioeconomic position

More people living in the most versus least deprived quintile of areas attended ED in a medium quality Canadian study by Mbuya-Bienge et al[27] (Table 4). Results were stratified by number of LTCs: for people with two LTCs 8.9% (95%CI 8.6-9.1) living in most deprived areas attended ED versus 6.3% (95%CI 6.1-6.6) in the least deprived areas; for people with three LTCs 12.0% (95%CI 11.6-12.5) versus 9.5% (95%CI 9.1-10.0); for people with ≥ four LTCs 18.3% (95%CI 17.9-18.8) versus 16.8% (95%CI 16.3-17.4). Likewise, more people living in the most versus least deprived area quintiles were hospitalised in the Spanish study by Orueta et al,[31] rated as medium quality. This study also stratified the number of LTCs and found that in people with four to six LTCs, 26% living in most deprived areas were hospitalised versus 22% of people in the least deprived areas; for people with seven to nine LTCs, 48% versus 44% (no 95%CIs or cohort size from which to derive these). However, in people with ≥10 LTCs, rates of hospitalisation were lower in people living in the most, 68%, versus least, 70%, deprived areas. Neither study examining the effect of SEP performed regression analyses.

For peer review only - http://bmjopen.bmj.com/site/about/guidelines.xhtml

Table 4. Area exposure study results

Study exposure	Study	Outcome	Methodological quality rating	Covariates	Result metric	Key Results	Association at 95% for IRR*
Area SEP	Payne et al[32] Scotland, Hong Kong, China	Hospitalisation	High	Stratified by number of LTC and sex	Predicted probability and 95%CIs	Higher probability of unplanned hospitalisation in men with physical only multimorbidity in most (6.4% [95%CI 5.8-7.2]) versus least (4.2% [95%CI 3.8-4.7]) deprived areas<br>Higher probability of unplanned hospitalisation in men with physical and mental multimorbidity in most (12.1% [95%CI 11.0-13.6]) versus least (8.1% [95%CI 7.3-9.2]) deprived areas	NA
	Mbuya-Bienge et al[27] Canada	ED attendance	Medium	Non-adjusted prevalence rates	Prevalence rates % and 95%CIs	Lower rates of ED use in most (8.9% [95%CI 8.6-9.1]) versus least (6.3% [95%CI 6.1-6.6]) deprived areas	NA
	Orueta et al[31] Spain	Hospitalisation	Medium	Non-adjusted prevalence rates	Prevalence rates % (no 95%CIs)	Lower rates of hospitalisation in most (26%) versus least (22%) deprived area quintile	NA
Rurality	Pati et al[29] India	Inpatient and outpatient hospital use	Medium	Age, sex, ethnicity, education, SEP, rurality	IRR and 95%CIs	No difference in hospitalisation between urban and rural areas (IRR 1.09 [95%CI 0.64-1.88])	-

* "NA" study outcomes were not ORs or IRRs, "✓" studies where an association at 95% significance was found, "-" studies where no association at 95% significance was found, "✓/-" studies where results were mixed

For peer review only - http://bmjopen.bmj.com/site/about/guidelines.xhtml

For peer review only

For peer review only - http://bmjopen.bmj.com/site/about/guidelines.xhtml

Payne et al,[32] a Scottish study rated as high quality, examined the association between SEP and hospitalisation through regression analyses, stratified by sex number of LTCs, and presence of physical only or physical and mental LTCs. Living in the most versus least deprived quintile areas was associated with a higher probability of hospitalisation for all groups examined (for example, men with physical only multimorbidity had a higher probability of unplanned hospitalisation in most (6.4% [95%CI 0.05.8-7.2]) versus least (4.2% [95%CI 3.8-4.7]) deprived areas. These effects were more pronounced for people with more versus less LTCs, with a dose response seen between number of LTCs and increase in probability of hospitalisation, and for people with physical and mental multimorbidity versus physical only multimorbidity.

Area rurality

Pati et al[29] examined the relationship between urban versus rural residence and any hospital attendance in India, and found a relationship that did not reach statistical significance (urban versus rural residence IRR 1.09 [95%CI 0.64-1.88]).

Discussion

Principal findings

This systematic review and narrative synthesis describe existing evidence of associations between household and area exposures and ED attendance and hospitalisation outcomes in people with multimorbidity. In people with multimorbidity, household food insecurity was associated with hospitalisation, and rates of ED attendance and hospitalisation, and probability of hospitalisation, were higher in people living in the most versus least deprived areas. There is non-conclusive evidence that in people with multimorbidity, household income is associated with ED attendance and hospitalisation, with differing relationships seen depending on study location and how healthcare is organised and paid for in different settings. No statistically significant relationships were found in people with multimorbidity between ED attendance or hospitalisation with marital status, living with others with multimorbidity, or living in urban versus rural areas.

Strengths and weaknesses

Strengths of the study include comprehensive and systematic searching of many electronic databases, with an iteratively developed set of search terms, extraction of data by two researchers, and assessment of study quality with adaptation of a standard method to meet the needs of the review, and analysis using a standardised approach to narrative reporting. There are several limitations of our study. There are few included studies because analyses of household and area variables associated with ED attendance or hospitalisation has not been commonly done in people with multimorbidity, and there is considerable heterogeneity in the definition and measurement of multimorbidity, exposures, outcomes,

For peer review only - http://bmjopen.bmj.com/site/about/guidelines.xhtml

and analysis methods precluding meta-analysis. Studies were from both high-income and low-and-middle income countries, where contextual factors may hold different meanings, making direct comparisons across studies difficult. Finally, although multimorbidity was defined as the presence of ≥ 2 LTCs in all studies, there was a diverse range of LTCs included within counts, which will affect prevalence rates and therefore reliability of comparison between studies.[20]

Comparison with existing literature

Household income was not conclusively associated with ED attendance or hospitalisation in people with multimorbidity, however small sample sizes resulting from stratified analyses may have contributed to some estimates not reaching statistical significance in the Canadian study examining this.[26] Different directions of association were found between household income and hospitalisation in different countries and healthcare systems by Wang et al.[34] Lower household income was associated with higher likelihood of hospitalisation in areas with universal healthcare provision (free at the point of care in Scotland and heavily subsidised in Hong Kong public hospitals). These results reflect findings from a study of older people (not stratified by multimorbidity) in Stockholm, where healthcare is free at the point of delivery, found women living in households with the lowest versus highest income households had an increased probability (OR 2.91 [95% CI 2.52-3.36]) of ED attendance.[37] However lower household income was associated with lower probability of hospitalisation in areas where healthcare is largely or solely privately funded (mainland China and the private hospitals in Hong Kong), demonstrating that lack of access to equitable care can further exacerbate health inequalities by reducing access to those with lowest household income.[34]

Household hunger was associated with hospitalisation in both concordant and discordant multimorbidity in Tanzania,[33] which is similar to a study from the United States of America where household hunger was associated with increased hospitalisations in a general adult population (OR 1.36 [95%CI 1.22–1.52]).[38] The severity of household hunger is likely to vary widely depending on study setting, making these findings context specific. Marital status in this study was assumed to be a household variable. Marital status could also be considered a characteristic of the individual or of a household. It is commonly examined as a proxy for not living alone and having increased household social support in studies using routine data,[12, 39] and in this study was therefore assumed to be a household variable." However, actual living arrangements (including cohabitation as an unmarried couple) are more closely associated with health outcomes but are less examined due to data limitations.[39] We expected to see a protective effect between being married resulting in reduced ED attendance and hospitalisation, but the included studies found no statistically significant associations in both directions. The three studies examining area SEP reported increased ED attendance and hospitalisation in people with multimorbidity living in the

For peer review only - http://bmjopen.bmj.com/site/about/guidelines.xhtml

most deprived fifth of areas, consistent with existing literature of general adult populations people living in areas of lower area SEP are higher users of healthcare.[8, 9, 40]

In our review, one study examined the association between living in urban versus rural areas and any hospital attendance in people with multimorbidity, finding a non-statistically significant OR>1 for people living in urban areas. Similarly, a study examining a population of older adults in China,[41] not stratified by multimorbidity status, found that living in urban areas was associated with increased likelihood of inpatient care.

Implications

Some of the variation in results across studies may be explained by the heterogeneity in the definition of multimorbidity. Future studies should standardise multimorbidity definitions (for example, the presence of two or more LTCs from a standardised list of LTCs) to align with other studies and improve comparability.[20] Comparative research that further explores variation in associations in different healthcare systems would be valuable, testing models of exposure to outcome associations. Such studies are needed to understand the mechanisms by which observed associations happen, examining whether household and area exposures interact with each other and with multimorbidity, considering whether these factors mediate, moderate, or have a multiplicative effect. No included articles examined supply factors associated with hospital use, for example area wide demand issues such as distance to services, or supply issues such as access to primary care services. These factors are likely to influence hospital use rates in people with multimorbidity and require further study. Use of longitudinal study designs could be used to evaluate the evolution of increased risk or protection associated with the context in which an individual lives. Only two area-level exposures have been examined, despite more extensive literature examining the place-based determinants of multimorbidity,[19] emphasising the need to examine associations between outcomes in people with multimorbidity and a broader range of area-level factors, including green spaces, social cohesion, and provision of services.

Conclusions

In conclusion, although available evidence in the study area is limited, there is some evidence that household and area exposures are associated with increased risk of ED attendance and hospitalisation in people with multimorbidity. Since interventions to reduce hospital attendance may be more effective if they also account for the context in which people live, there is a need for further research to examine the contribution of a wider range of contextual exposures to hospital attendance in people with multimorbidity.

Word count: 3970

For peer review only - http://bmjopen.bmj.com/site/about/guidelines.xhtml

Figure 1. Study selection, PRISMA diagram

For peer review only

Declarations

Funding: CM received funding from the NHS Education for Scotland Academic Fellowship and Medical Research Council MR/W000253/1 to support this work.

Role of the funding source: The funder had no role development of study design, data collection and interpretation, or writing of the final report. The corresponding author had full access all data and had final responsibility for submitting for publication.

Competing interests: None.

Data availability statement: No additional data are available. No data sharing agreement was obtained for this study because data analysed was obtained from previous published studies where data sharing agreements are in place.

Ethical approvals: Not applicable. No ethical approval was obtained for this study because data analysed was obtained from previous published studies where ethical approvals are in place.

Contributors: CM and BG conceived the study and contributed to the design of the project. CM created the search strategy and BG, SWM, JP, AL, and CD contributed to subsequent iterations. CM, HF, EL, and TC conducted the search. CM and BG extracted and analysed the data. CM drafted the paper, BG, SWM, JP, AM, AL, and CD critically commented on the manuscript. All authors reviewed drafts and approved the final version

For peer review only

References

1. National population projections: 2018-based. Office for National Statistics. Availalble at https://www.ons.gov.uk/peoplepopulationandcommunity/populationandmigration/populationprojections/bulletins/nationalpopulationprojections/2018based [accessed 19 September 2021] [

2. Multimorbidity: a priority for global health research. The Academy of Medical Sciences. Available at https://acmedsci.ac.uk/policy/policy-projects/multimorbidity [accessed 18 April 2022] [

3. Johansson S, Rosengren A, Young K, Jennings E. Mortality and morbidity trends after the first year in survivors of acute myocardial infarction: a systematic review. BMC cardiovascular disorders. 2017;17(1):53.

4. Jones PB. Adult mental health disorders and their age at onset. British journal of psychiatry. 2013;202(s54):s5-s10.

5. Hanlon P, Nicholl BI, Jani BD, Lee D, McQueenie R, Mair FS. Frailty and pre-frailty in middle-aged and older adults and its association with multimorbidity and mortality: a prospective analysis of 493 737 UK Biobank participants. The Lancet Public health. 2018;3(7):e323-e32.

6. Williams JS, Egede LE. The Association Between Multimorbidity and Quality of Life, Health Status and Functional Disability. The American journal of the medical sciences. 2016;352(1):45-52.

7. Cassell A, Edwards D, Harshfield A, Rhodes K, Brimicombe J, Payne R, et al. The epidemiology of multimorbidity in primary care: a retrospective cohort study. The British journal of general practice : the journal of the Royal College of General Practitioners. 2018;68(669):e245.

8. Luben R, Hayat S, Wareham N, Pharoah PP, Khaw KT. Sociodemographic and lifestyle predictors of incident hospital admissions with multimorbidity in a general population, 1999-2019: the EPIC-Norfolk cohort. Bmj Open. 2020;10(9).

9. McCormack LA, Madlock-Brown C. Social Determinant of Health Documentation Trends and Their Association with Emergency Department Admissions. AMIA Annual Symposium proceedings AMIA Symposium. 2020;2020:823-32.

10. Huygen FJA. Family medicine : the medical life history of families / F.J.A. Huygen. London: Royal College of General Practitioners; 1990.

11. Longman JM, I Rolfe M, Passey MD, Heathcote KE, Ewald DP, Dunn T, et al. Frequent hospital admission of older people with chronic disease: A cross-sectional survey with telephone follow-up and data linkage. BMC Health Services Research. 2012;12(1).

12. Ridge A, Peterson GM, Kitsos A, Seidel BM, Anderson V, Nash R. Potentially Preventable Hospitalisations in rural Community-Dwelling Patients. Internal medicine journal. 2021.

13. Bu F, Philip K, Fancourt D. Social isolation and loneliness as risk factors for hospital admissions for respiratory disease among older adults. Thorax. 2020;75(7):597-9.

14. Luben R, Hayat S, Khawaja A, Wareham N, Pharoah PP, Khaw K-T. Residential area deprivation and risk of subsequent hospital admission in a British population: the EPIC-Norfolk cohort. BMJ open. 2019;9(12):e031251-e.

15. Mohnen SM, Schneider S, Droomers M. Neighborhood characteristics as determinants of healthcare utilization – a theoretical model. Health economics review. 2019;9(1):1-9.

16. Griffin E, McCarthy JP, Thomas F, Kingham S. New Zealand Healthline call data used to measure the effect of travel time on the use of the emergency department. Social science & medicine (1982). 2017;179:91-6.

17. PRISMA: Transparent Reporting of Systematic Reviews and Meta-Analyses. Aavailable at http://www.prisma-statement.org [accessed 7 September 2021].

18. A conceptual framework for action on the social determinants of health: social determinants of health discussion paper 2. World Health Organisation. Available at https://apps.who.int/iris/bitstream/handle/10665/44489/9789241500852_eng.pdf?sequence=1&isAllowed=y [accessed 28 September 2021] [

For peer review only - http://bmjopen.bmj.com/site/about/guidelines.xhtml

19. Ingram E, Ledden S, Beardon S, Gomes M, Hogarth S, McDonald H, et al. Household and area-level social determinants of multimorbidity: a systematic review. Journal of epidemiology and community health (1979). 2021;75(3):232-41.
20. Ho IS-S, Azcoaga-Lorenzo A, Akbari A, Black C, Davies J, Hodgins P, et al. Examining variation in the measurement of multimorbidity in research: a systematic review of 566 studies. The Lancet Public Health. 2021;6(8):e587-e97.
21. Stafford M, Deeny SR, Dreyer K, Shand J. Multiple long-term conditions within households and use of health and social care: a retrospective cohort study. BJGP open. 2021;5(2):BJGPO.2020.0134.
22. Innovation VH. Covidence systematic review software. Melbourne: Veritas Health Innovation VIC, 2016. [
23. The Newcastle-Ottowa Assessment Scale (NOAS) for assessing the quality of nonrandomised studies in meta-analyses. Available at http://www.ohri.ca/programs/clinical_epidemiology/oxford.asp [accessed 5 October 2021] [
24. Campbell M, McKenzie JE, Sowden A, Katikireddi SV, Brennan SE, Ellis S, et al. Synthesis without meta-analysis (SWiM) in systematic reviews: reporting guideline. BMJ. 2020;368:l6890-l.
25. Thomas JHJ. Cochrane Handbook for Systematic Reviews of Interventions. Cochrane Training. 2019, version 6.
26. Fisher KA, Griffith LE, Gruneir A, Upshur R, Perez R, Favotto L, et al. Effect of socio-demographic and health factors on the association between multimorbidity and acute care service use: population-based survey linked to health administrative data. Bmc Health Services Research. 2021;21(1).
27. Mbuya-Bienge C, Simard M, Gaulin M, Candas B, Sirois C. Does socio-economic status influence the effect of multimorbidity on the frequent use of ambulatory care services in a universal healthcare system? A population-based cohort study. Bmc Health Services Research. 2021;21(1).
28. Lu L, Zeng J, Zeng Z. What limits the utilization of health services among china labor force? analysis of inequalities in demographic, socio-economic and health status. International journal for equity in health. 2017;16(1):30-.
29. Pati S, Swain S, Hussain MA, Kadam S, Salisbury C. Prevalence, correlates, and outcomes of multimorbidity among patients attending primary care in Odisha, India. Annals of Family Medicine. 2015;13(5):446-50.
30. Chung RY, Mercer SW, Yip BHK, Chan SWC, Lai FTT, Wang HHX, et al. The association between types of regular primary care and hospitalization among people with and without multimorbidity: A household survey on 25,780 Chinese. Scientific Reports. 2016;6.
31. Orueta JF, García-Álvarez A, Alonso-Morán E, Vallejo-Torres L, Nuño-Solinis R. Socioeconomic variation in the burden of chronic conditions and health care provision - Analyzing administrative individual level data from the Basque Country, Spain. BMC Public Health. 2013;13(1).
32. Payne RA, Abel GA, Guthrie B, Mercer SW. The effect of physical multimorbidity, mental health conditions and socioeconomic deprivation on unplanned admissions to hospital: A retrospective cohort study. CMAJ. 2013;185(5):E221-E8.
33. Tomita A, Leyna GH, Kim HY, Moodley Y, Mpolya E, Mogeni P, et al. Patterns of multimorbidity and their association with hospitalisation: a population-based study of older adults in urban Tanzania. Age and Ageing. 2021;50(4):1349-60.
34. Wang HHX, Wang JJ, Lawson KD, Wong SYS, Wong MCS, Li FJ, et al. Relationships of multimorbidity and income with hospital admissions in 3 health care systems. Annals of Family Medicine. 2015;13(2):164-7.
35. Cassell A, Edwards D, Harshfield A, Rhodes K, Brimicombe J, Payne R, et al. The epidemiology of multimorbidity in primary care: A retrospective cohort study. British Journal of General Practice. 2018;68(669):e245-e51.

For peer review only - http://bmjopen.bmj.com/site/about/guidelines.xhtml

36. Identifying property and street information. Available at https://www.gov.uk/government/publications/open-standards-for-government/identifying-property-and-street-information [accessed 19 March 2022] [

37. Doheny M, Agerholm J, Orsini N, Schon P, Burstrom B. Socio-demographic differences in the frequent use of emergency department care by older persons: a population-based study in Stockholm County. Bmc Health Services Research. 2019;19.

38. Wray CM, Tang J, Lopez L, Hoggatt K, Keyhani S. Association of Social Determinants of Health and Their Cumulative Impact on Hospitalization Among a National Sample of Community-Dwelling US Adults. Journal of general internal medicine : JGIM. 2021.

39. Herm A, Anson JON, Poulain M. Living arrangements and marital status: a register-based study of survival of older adults in Belgium at the beginning of the 21st century. Ageing and society. 2016;36(10):2141-62.

40. Giannouchos TV, Kum H-C, Foster MJ, Ohsfeldt RL. Characteristics and predictors of adult frequent emergency department users in the United States: A systematic literature review. Journal of evaluation in clinical practice. 2019;25(3):420-33.

41. Gong CH, Kendig H, He X. Factors predicting health services use among older people in China: An analysis of the China Health and Retirement Longitudinal Study 2013. BMC health services research. 2016;16(1):63-.

For peer review only

Tables and Figures

Figure 1. Study selection, PRISMA diagram

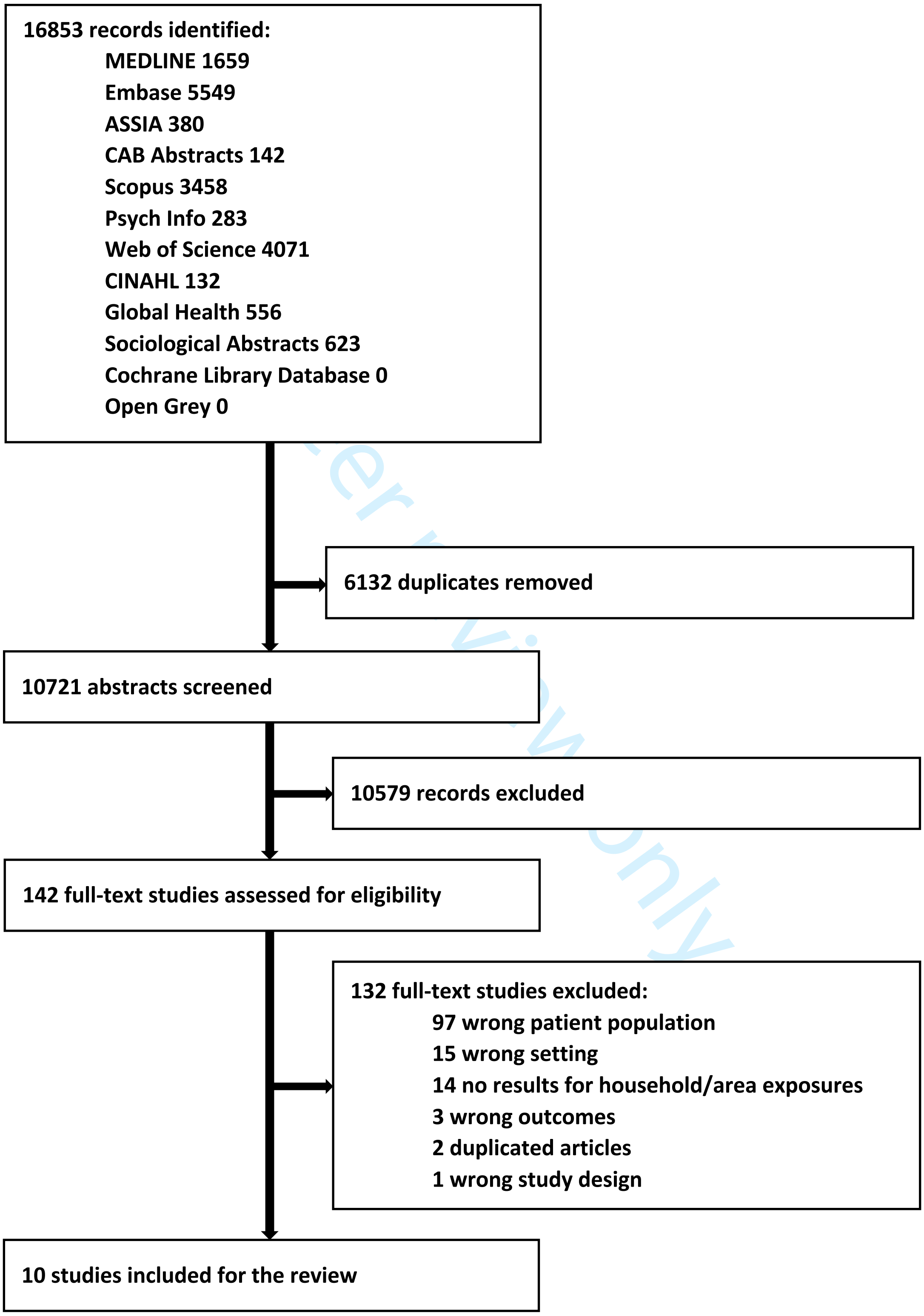

Supplementary File

Table S1. Database search strategies

**Database (number of articles)**	**Search strategy**
MedLine (1659)	(MULTIMORBIDITY/ or "multimorbidit*".mp. or "multi-morbidit*".mp. or "comorbidit*".mp. or "co-morbidit*".mp. or "polymorbidit*".mp. or "poly-morbidit*".mp. or"multicondition*".mp. or "multi-condition*".mp. or "multiple chronic condition*".mp. or "morbidity burden*".mp. or ((multiple or coexisting or co-existing or concurrent or con-current or comorbid* or co-morbid* or several*) adj2 (disease* or illness* or condition* or diagnosis* or morbid* or patholog*))) AND ("household*".mp. or "coresiden*".mp. or "co-residen*".mp. or ((household or family or domestic or residen*) adj2 (composition* or member* or characterist* or dweller*)) or "neighborhood*".mp. or "neighbourhood*".mp. or "small area*".mp. or "small-area*".mp. or "place".mp. or "communit*".mp. or "census tracts".mp. or "census block group".mp. or "zip code*".mp. or "post code*".mp. or "electoral ward*".mp. or "data zone*".mp. or "output area*".mp. or "lower super output area*".mp. or "municipalit*".mp. or "provinc*".mp. or SOCIOECONOMIC FACTORS/ or INCOME/ or "socioeconomic".mp. or "socio-economic".mp. or "social capital".mp. or "inequalit*".mp. or "disparit*".mp. or "income".mp. or "wealth".mp. or "financial problem*".mp. or "financial difficult*".mp. or "vocation*".mp. or "training opportunit*".mp. or "employment opportunit*".mp. or "occupation*".mp. or "opportunit*".mp. or "job opportunit*".mp. or "job insecurit".mp. or "education*" or "opportunit*".mp. or "education*".mp. or "achiev*".mp. or "education*".mp. or "quality".mp. or "fast-food".mp. or "fast food".mp. or "healthy food".mp. or "social determinant".mp. or "wider determinant".mp. or POVERTY AREAS/ or POVERTY/ or "neighbourhood disorder".mp. or "overcrowding".mp. or "public housing".mp. or "population density".mp. or "urban".mp. or "ghetto".mp. or "rural".mp. or "slum".mp. or "estate".mp. or "poverty area*".mp. or "physical environment*".mp. or "built environment*".mp. or "living standard*".mp. or "family structure".mp. or "family breakdown".mp. or "family disintegrat*".mp. or "single parent*".mp. or "housing tenure".mp. or "Townsend".mp. or "Carstairs".mp. or "Index of Multiple Deprivation".mp. or "Nam-Powers".mp. or "Hollingshead Index".mp. or "Breadline Britain Index".mp. or "inverse care law".mp. or "Singh ind*".mp. or "Yost ind*".mp. or "Kolak ind*".mp. or "social determinants of health".mp. or "neighbourhood archetype*".mp. or "segregation indic*".mp. or "ecological inversion".mp. or "ecological fallacy".mp. or "ecological bias".mp. or "depriv*".mp. or "impoverish*".mp. or "disadvantage*".mp. or "community cohesion".mp. or "community network".mp. or "community support".mp. or "transport* quality".mp. or "transport* services".mp. or "transport link*".mp. or "public transport*".mp. or "travel time".mp. or "community resource access*".mp. or "walkability".mp. or "public space".mp. or "green space".mp. or "open space".mp. or "recreation*".mp. or "leisure activit*".mp. or "food outlet*".mp. or "food environment".mp. or "air pollution".mp. or "particulate matter".mp. or "nitrogen dioxide".mp. or "sulphur dioxide".mp. or "sulfur dioxide".mp. or "carbon monoxide".mp. or "crime*".mp.) AND ("hospital admission*".mp. or "hospitalisation".mp. or "hospitalization".mp. or "emergency admission*".mp. or "unscheduled admission*".mp. or "unplanned admission*".mp. or "emergency department".mp. or "accident and emergency".mp.)<br>limit to (english language and yr="2000-Current")
EMBASE (5549)	("multimorbidit*".mp. or "multi-morbidit*".mp. or "comorbidit*".mp. or "co-morbidit*".mp. or "polymorbidit*".mp. or "poly-morbidit*".mp. or"multicondition*".mp. or "multi-condition*".mp. or "multiple chronic condition*".mp. or "morbidity burden*".mp. or "multiple long-term condition*".mp. or ((multiple or coexisting or co-existing or concurrent or con-current or comorbid* or co-morbid* or several*) adj2 (disease* or illness* or condition* or diagnosis* or morbid* or patholog*))) AND ("household*".mp. or "coresiden*".mp. or "co-residen*".mp. or ((household or family or domestic or residen*) adj2 (composition* or member* or characterist* or dweller*)) or "neighborhood*".mp. or "neighbourhood*".mp. or "small area*".mp. or "small-area*".mp. or "place".mp. or "communit*".mp. or "census tracts".mp. or "census block group".mp. or "zip code*".mp. or "post code*".mp. or "electoral ward*".mp. or "data zone*".mp. or "output area*".mp. or "lower super output area*".mp. or "municipalit*".mp. or "provinc*".mp. or "socioeconomic".mp. or "socio-economic".mp. or "social capital".mp. or "inequalit*".mp. or "disparit*".mp. or "income".mp. or "wealth".mp. or "financial problem*".mp. or "financial difficult*".mp. or "vocation*".mp. or "training opportunit*".mp. or "employment opportunit*".mp. or "occupation*".mp. or "opportunit*".mp. or "job opportunit*".mp. or "job insecurit".mp. or "education*" or "opportunit*".mp. or "education*".mp. or "achiev*".mp. or "education*".mp. or "quality".mp. or "fast-food".mp. or "fast food".mp. or "healthy food".mp. or "social determinant".mp. or "wider determinant".mp. or "neighbourhood disorder".mp. or "overcrowding".mp. or "public housing".mp. or "population density".mp. or "urban".mp. or "ghetto".mp. or "rural".mp. or "slum".mp. or "estate".mp. or "poverty area*".mp. or "physical environment*".mp. or "built environment*".mp. or "living standard*".mp. or "family structure".mp. or "family breakdown".mp. or "family disintegrat*".mp. or "single parent*".mp. or "housing tenure".mp. or "Townsend".mp. or "Carstairs".mp. or "Index of Multiple Deprivation".mp. or "Nam-Powers".mp. or "Hollingshead Index".mp. or "Breadline Britain Index".mp. or "inverse care law".mp. or "Singh ind*".mp. or "Yost ind*".mp. or "Kolak ind*".mp. or "social determinants of health".mp. or "neighbourhood archetype*".mp. or "segregation indic*".mp. or "ecological inversion".mp. or "ecological fallacy".mp. or "ecological bias".mp. or "depriv*".mp. or "impoverish*".mp. or "disadvantage*".mp. or "community cohesion".mp. or "community network".mp. or "community support".mp. or "transport* quality".mp. or "transport* services".mp. or "transport link*".mp. or "public transport*".mp. or "travel time".mp. or "community resource access*".mp. or "walkability".mp. or "public space".mp. or "green space".mp. or "open space".mp. or "recreation*".mp. or "leisure activit*".mp. or "food

For peer review only

	outlet*".mp. or "food environment".mp. or "air pollution".mp. or "particulate matter".mp. or "nitrogen dioxide".mp. or "sulphur dioxide".mp. or "sulfur dioxide".mp. or "carbon monoxide".mp. or "crime*".mp.) AND ("admission*".mp. or "hospital admission*".mp. or "hospitalisation".mp. or "hospitalization".mp. or "emergency admission*".mp. or "unscheduled admission*".mp. or "unplanned admission*".mp. or "emergency department".mp. or "accident and emergency".mp.)<br>limit to (english language and yr="2000-Current")
Global Health (556)	("multimorbidit*".mp. or "multi-morbidit*".mp. or "comorbidit*".mp. or "co-morbidit*".mp. or "polymorbidit*".mp. or "poly-morbidit*".mp. or"multicondition*".mp. or "multi-condition*".mp. or "multiple chronic condition*".mp. or "morbidity burden*".mp. or ((multiple or coexisting or co-existing or concurrent or con-current or comorbid* or co-morbid* or several*) adj2 (disease* or illness* or condition* or diagnosis* or morbid* or patholog*))) AND ("household*".mp. or "coresiden*".mp. or "co-residen*".mp. or ((household or family or domestic or residen*) adj2 (composition* or member* or characterist* or dweller*)) or "neighborhood*".mp. or "neighbourhood*".mp. or "small area*".mp. or "small-area*".mp. or "place".mp. or "communit*".mp. or "census tracts".mp. or "census block group".mp. or "zip code*".mp. or "post code*".mp. or "electoral ward*".mp. or "data zone*".mp. or "output area*".mp. or "lower super output area*".mp. or "municipalit*".mp. or "provinc*".mp. or "socioeconomic".mp. or "socio-economic".mp. or "social capital".mp. or "inequalit*".mp. or "disparit*".mp. or "income".mp. or "wealth".mp. or "financial problem*".mp. or "financial difficult*".mp. or "vocation*".mp. or "training opportunit*".mp. or "employment opportunit*".mp. or "occupation*".mp. or "opportunit*".mp. or "job opportunit*".mp. or "job insecurit".mp. or "education*" or "opportunit*".mp. or "education*".mp. or "achiev*".mp. or "education*".mp. or "quality".mp. or "fast-food".mp. or "fast food".mp. or "healthy food".mp. or "social determinant".mp. or "wider determinant".mp. or "neighbourhood disorder".mp. or "overcrowding".mp. or "public housing".mp. or "population density".mp. or "urban".mp. or "ghetto".mp. or "rural".mp. or "slum".mp. or "estate".mp. or "poverty area*".mp. or "physical environment*".mp. or "built environment*".mp. or "living standard*".mp. or "family structure".mp. or "family breakdown".mp. or "family disintegrat*".mp. or "single parent*".mp. or "housing tenure".mp. or "Townsend".mp. or "Carstairs".mp. or "Index of Multiple Deprivation".mp. or "Nam-Powers".mp. or "Hollingshead Index".mp. or "Breadline Britain Index".mp. or "inverse care law".mp. or "Singh ind*".mp. or "Yost ind*".mp. or "Kolak ind*".mp. or "social determinants of health".mp. or "neighbourhood archetype*".mp. or "segregation indic*".mp. or "ecological inversion".mp. or "ecological fallacy".mp. or "ecological bias".mp. or "depriv*".mp. or "impoverish*".mp. or "disadvantage*".mp. or "community cohesion".mp. or "community network".mp. or "community support".mp. or "transport* quality".mp. or "transport* services".mp. or "transport link*".mp. or "public transport*".mp. or "travel time".mp. or "community resource access*".mp. or "walkability".mp. or "public space".mp. or "green space".mp. or "open space".mp. or "recreation*".mp. or "leisure activit*".mp. or "food outlet*".mp. or "food environment".mp. or "air pollution".mp. or "particulate matter".mp. or "nitrogen dioxide".mp. or "sulphur dioxide".mp. or "sulfur dioxide".mp. or "carbon monoxide".mp. or "crime*".mp.) AND ("hospital admission*".mp. or "hospitalisation".mp. or "hospitalization".mp. or "emergency admission*".mp. or "unscheduled admission*".mp. or "unplanned admission*".mp. or "emergency department".mp. or "accident and emergency".mp.)<br>limit to (english language and yr="2000-Current")
PsychInfo (283)	("multimorbidit*".mp. or "multi-morbidit*".mp. or "comorbidit*".mp. or "co-morbidit*".mp. or "polymorbidit*".mp. or "poly-morbidit*".mp. or"multicondition*".mp. or "multi-condition*".mp. or "multiple chronic condition*".mp. or "morbidity burden*".mp. or ((multiple or coexisting or co-existing or concurrent or con-current or comorbid* or co-morbid* or several*) adj2 (disease* or illness* or condition* or diagnosis* or morbid* or patholog*))) AND ("household*".mp. or "coresiden*".mp. or "co-residen*".mp. or ((household or family or domestic or residen*) adj2 (composition* or member* or characterist* or dweller*)) or "neighborhood*".mp. or "neighbourhood*".mp. or "small area*".mp. or "small-area*".mp. or "place".mp. or "communit*".mp. or "census tracts".mp. or "census block group".mp. or "zip code*".mp. or "post code*".mp. or "electoral ward*".mp. or "data zone*".mp. or "output area*".mp. or "lower super output area*".mp. or "municipalit*".mp. or "provinc*".mp. or "socioeconomic".mp. or "socio-economic".mp. or "social capital".mp. or "inequalit*".mp. or "disparit*".mp. or "income".mp. or "wealth".mp. or "financial problem*".mp. or "financial difficult*".mp. or "vocation*".mp. or "training opportunit*".mp. or "employment opportunit*".mp. or "occupation*".mp. or "opportunit*".mp. or "job opportunit*".mp. or "job insecurit".mp. or "education*" or "opportunit*".mp. or "education*".mp. or "achiev*".mp. or "education*".mp. or "quality".mp. or "fast-food".mp. or "fast food".mp. or "healthy food".mp. or "social determinant".mp. or "wider determinant".mp. or "neighbourhood disorder".mp. or "overcrowding".mp. or "public housing".mp. or "population density".mp. or "urban".mp. or "ghetto".mp. or "rural".mp. or "slum".mp. or "estate".mp. or "poverty area*".mp. or "physical environment*".mp. or "built environment*".mp. or "living standard*".mp. or "family structure".mp. or "family breakdown".mp. or "family disintegrat*".mp. or "single parent*".mp. or "housing tenure".mp. or "Townsend".mp. or "Carstairs".mp. or "Index of Multiple Deprivation".mp. or "Nam-Powers".mp. or "Hollingshead Index".mp. or "Breadline Britain Index".mp. or "inverse care law".mp. or "Singh ind*".mp. or "Yost ind*".mp. or "Kolak ind*".mp. or "social determinants of health".mp. or "neighbourhood archetype*".mp. or "segregation indic*".mp. or "ecological inversion".mp. or "ecological fallacy".mp. or "ecological bias".mp. or "depriv*".mp. or "impoverish*".mp. or "disadvantage*".mp. or "community cohesion".mp. or "community network".mp. or "community support".mp. or "transport* quality".mp. or "transport* services".mp. or "transport link*".mp. or "public transport*".mp. or "travel time".mp. or "community resource access*".mp. or "walkability".mp. or "public space".mp. or "green space".mp. or "open space".mp. or "recreation*".mp. or "leisure activit*".mp. or "food outlet*".mp. or "food environment".mp. or "air pollution".mp. or "particulate matter".mp. or "nitrogen dioxide".mp. or "sulphur dioxide".mp. or "sulfur dioxide".mp. or "carbon monoxide".mp. or "crime*".mp.) AND

For peer review only

	("hospital admission*".mp. or "hospitalisation".mp. or "hospitalization".mp. or "emergency admission*".mp. or "unscheduled admission*".mp. or "unplanned admission*".mp. or "emergency department".mp. or "accident and emergency".mp.)<br>limit to (english language and yr="2000-Current")
ASSIA (380)	("multimorbidit*" or "multi-morbidit*" or "comorbidit*" or "co-morbidit*" or "polymorbidit*" or "poly-morbidit*" or "multicondition*" or "multi-condition*" or "multiple chronic condition*" or "morbidity burden*" or ((multiple or coexisting or co-existing or concurrent or con-current or comorbid* or co-morbid* or several*) adj2 (disease* or illness* or condition* or diagnosis* or morbid* or patholog*))) AND ("household*" or "coresiden*" or "co-residen*" or ((household or family or domestic or residen*) adj2 (composition* or member* or characterist* or dweller*)) or "neighborhood*" or "neighbourhood*" or "small area*" or "small-area*" or "place" or "communit*" or "census tracts" or "census block group" or "zip code*" or "post code*" or "electoral ward*" or "data zone*" or "output area*" or "lower super output area*" or "municipalit*" or "provinc*" or "socioeconomic" or "socio-economic" or "social capital" or "inequalit*" or "disparit*" or "income" or "wealth" or "financial problem*" or "financial difficult*" or "vocation*" or "training opportunit*" or "employment opportunit*" or "occupation* opportunit*" or "job opportunit*" or "job insecurit" or "education* opportunit*" or "education* achiev*" or "education* quality" or "fast-food" or "fast food" or "healthy food" or "social determinant" or "wider determinant" or "neighbourhood disorder" or "overcrowding" or "public housing" or "population density" or "urban" or "ghetto" or "rural" or "slum" or "estate" or "poverty area*" or "physical environment*" or "built environment*" or "living standard*" or "family structure" or "family breakdown" or "family disintegrat*" or "single parent*" or "housing tenure" or "Townsend" or "Carstairs" or "Index of Multiple Deprivation" or "Nam-Powers" or "Hollingshead Index" or "Breadline Britain Index" or "inverse care law" or "Singh ind*" or "Yost ind*" or "Kolak ind*" or "social determinants of health" or "neighbourhood archetype*" or "segregation indic*" or "ecological inversion" or "ecological fallacy" or "ecological bias" or "depriv*" or "impoverish*" or "disadvantage*" or "community cohesion" or "community network" or "community support" or "transport* quality" or "transport* services" or "transport link*" or "public transport*" or "travel time" or "community resource access*" or "walkability" or "public space" or "green space" or "open space" or "recreation*" or "leisure activit*" or "food outlet*" or "food environment" or "air pollution" or "particulate matter" or "nitrogen dioxide" or "sulphur dioxide" or "sulfur dioxide" or "carbon monoxide" or "crime*") AND ("hospital admission*" or "hospitalisation" or "hospitalization" or "emergency admission*" or "unscheduled admission*" or "unplanned admission*" or "emergency department" or "accident and emergency")<br>Publication date: after 01 January 2000
CAB Abstracts (142)	("multimorbidit*" or "multi-morbidit*" or "comorbidit*" or "co-morbidit*" or "polymorbidit*" or "poly-morbidit*" or "multicondition*" or "multi-condition*" or "multiple chronic condition*" or "morbidity burden*" or ((multiple or coexisting or co-existing or concurrent or con-current or comorbid* or co-morbid* or several*) adj2 (disease* or illness* or condition* or diagnosis* or morbid* or patholog*))) AND ("household*" or "coresiden*" or "co-residen*" or ((household or family or domestic or residen*) adj2 (composition* or member* or characterist* or dweller*)) or "neighborhood*" or "neighbourhood*" or "small area*" or "small-area*" or "place" or "communit*" or "census tracts" or "census block group" or "zip code*" or "post code*" or "electoral ward*" or "data zone*" or "output area*" or "lower super output area*" or "municipalit*" or "provinc*" or "socioeconomic" or "socio-economic" or "social capital" or "inequalit*" or "disparit*" or "income" or "wealth" or "financial problem*" or "financial difficult*" or "vocation*" or "training opportunit*" or "employment opportunit*" or "occupation* opportunit*" or "job opportunit*" or "job insecurit" or "education* opportunit*" or "education* achiev*" or "education* quality" or "fast-food" or "fast food" or "healthy food" or "social determinant" or "wider determinant" or "neighbourhood disorder" or "overcrowding" or "public housing" or "population density" or "urban" or "ghetto" or "rural" or "slum" or "estate" or "poverty area*" or "physical environment*" or "built environment*" or "living standard*" or "family structure" or "family breakdown" or "family disintegrat*" or "single parent*" or "housing tenure" or "Townsend" or "Carstairs" or "Index of Multiple Deprivation" or "Nam-Powers" or "Hollingshead Index" or "Breadline Britain Index" or "inverse care law" or "Singh ind*" or "Yost ind*" or "Kolak ind*" or "social determinants of health" or "neighbourhood archetype*" or "segregation indic*" or "ecological inversion" or "ecological fallacy" or "ecological bias" or "depriv*" or "impoverish*" or "disadvantage*" or "community cohesion" or "community network" or "community support" or "transport* quality" or "transport* services" or "transport link*" or "public transport*" or "travel time" or "community resource access*" or "walkability" or "public space" or "green space" or "open space" or "recreation*" or "leisure activit*" or "food outlet*" or "food environment" or "air pollution" or "particulate matter" or "nitrogen dioxide" or "sulphur dioxide" or "sulfur dioxide" or "carbon monoxide" or "crime*") AND ("hospital admission*" or "hospitalisation" or "hospitalization" or "emergency admission*" or "unscheduled admission*" or "unplanned admission*" or "emergency department" or "accident and emergency")<br>Publication date: after 01 January 2000
Web of Science (4071)	("multimorbidit*" or "multi-morbidit*" or "comorbidit*" or "co-morbidit*" or "polymorbidit*" or "poly-morbidit*" or "multicondition*" or "multi-condition*" or "multiple chronic condition*" or "morbidity burden*" or ((multiple or coexisting or co-existing or concurrent or con-current or comorbid* or co-morbid* or several*) adj2 (disease* or illness* or condition* or diagnosis* or morbid* or patholog*))) AND ("household*" or "coresiden*" or "co-residen*" or ((household or family or domestic or residen*) adj2 (composition* or member* or characterist* or dweller*)) or "neighborhood*" or "neighbourhood*" or "small area*" or "small-area*" or "place" or "communit*" or "census tracts" or "census block group" or "zip code*" or "post code*" or "electoral ward*" or "data zone*" or

For peer review only

	"output area*" or "lower super output area*" or "municipalit*" or "provinc*" or "socioeconomic" or "socio-economic" or "social capital" or "inequalit*" or "disparit*" or "income" or "wealth" or "financial problem*" or "financial difficult*" or "vocation*" or "training opportunit*" or "employment opportunit*" or "occupation* opportunit*" or "job opportunit*" or "job insecurit" or "education* opportunit*" or "education* achiev*" or "education* quality" or "fast-food" or "fast food" or "healthy food" or "social determinant" or "wider determinant" or "neighbourhood disorder" or "overcrowding" or "public housing" or "population density" or "urban" or "ghetto" or "rural" or "slum" or "estate" or "poverty area*" or "physical environment*" or "built environment*" or "living standard*" or "family structure" or "family breakdown" or "family disintegrat*" or "single parent*" or "housing tenure" or "Townsend" or "Carstairs" or "Index of Multiple Deprivation" or "Nam-Powers" or "Hollingshead Index" or "Breadline Britain Index" or "inverse care law" or "Singh ind*" or "Yost ind*" or "Kolak ind*" or "social determinants of health" or "neighbourhood archetype*" or "segregation indic*" or "ecological inversion" or "ecological fallacy" or "ecological bias" or "depriv*" or "impoverish*" or "disadvantage*" or "community cohesion" or "community network" or "community support" or "transport* quality" or "transport* services" or "transport link*" or "public transport*" or "travel time" or "community resource access*" or "walkability" or "public space" or "green space" or "open space" or "recreation*" or "leisure activit*" or "food outlet*" or "food environment" or "air pollution" or "particulate matter" or "nitrogen dioxide" or "sulphur dioxide" or "sulfur dioxide" or "carbon monoxide" or "crime*") AND ("hospital admission*" or "hospitalisation" or "hospitalization" or "emergency admission*" or "unscheduled admission*" or "unplanned admission*" or "emergency department" or "accident and emergency")<br>Language: English<br>Publication date: after 01 January 2000
Scopus (3458)	("multimorbidit*" or "multi-morbidit*" or "comorbidit*" or "co-morbidit*" or "polymorbidit*" or "poly-morbidit*" or "multicondition*" or "multi-condition*" or "multiple chronic condition*" or "morbidity burden*" or ((multiple or coexisting or co-existing or concurrent or con-current or comorbid* or co-morbid* or several*) adj2 (disease* or illness* or condition* or diagnosis* or morbid* or patholog*))) AND ("household*" or "coresiden*" or "co-residen*" or ((household or family or domestic or residen*) adj2 (composition* or member* or characterist* or dweller*)) or "neighborhood*" or "neighbourhood*" or "small area*" or "small-area*" or "place" or "communit*" or "census tracts" or "census block group" or "zip code*" or "post code*" or "electoral ward*" or "data zone*" or "output area*" or "lower super output area*" or "municipalit*" or "provinc*" or "socioeconomic" or "socio-economic" or "social capital" or "inequalit*" or "disparit*" or "income" or "wealth" or "financial problem*" or "financial difficult*" or "vocation*" or "training opportunit*" or "employment opportunit*" or "occupation* opportunit*" or "job opportunit*" or "job insecurit" or "education* opportunit*" or "education* achiev*" or "education* quality" or "fast-food" or "fast food" or "healthy food" or "social determinant" or "wider determinant" or "neighbourhood disorder" or "overcrowding" or "public housing" or "population density" or "urban" or "ghetto" or "rural" or "slum" or "estate" or "poverty area*" or "physical environment*" or "built environment*" or "living standard*" or "family structure" or "family breakdown" or "family disintegrat*" or "single parent*" or "housing tenure" or "Townsend" or "Carstairs" or "Index of Multiple Deprivation" or "Nam-Powers" or "Hollingshead Index" or "Breadline Britain Index" or "inverse care law" or "Singh ind*" or "Yost ind*" or "Kolak ind*" or "social determinants of health" or "neighbourhood archetype*" or "segregation indic*" or "ecological inversion" or "ecological fallacy" or "ecological bias" or "depriv*" or "impoverish*" or "disadvantage*" or "community cohesion" or "community network" or "community support" or "transport* quality" or "transport* services" or "transport link*" or "public transport*" or "travel time" or "community resource access*" or "walkability" or "public space" or "green space" or "open space" or "recreation*" or "leisure activit*" or "food outlet*" or "food environment" or "air pollution" or "particulate matter" or "nitrogen dioxide" or "sulphur dioxide" or "sulfur dioxide" or "carbon monoxide" or "crime*") AND ("hospital admission*" or "hospitalisation" or "hospitalization" or "emergency admission*" or "unscheduled admission*" or "unplanned admission*" or "emergency department" or "accident and emergency")<br>Publication date: after 01 January 2000
CINAHL (132)	("multimorbidit*" or "multi-morbidit*" or "comorbidit*" or "co-morbidit*" or "polymorbidit*" or "poly-morbidit*" or "multicondition*" or "multi-condition*" or "multiple chronic condition*" or "morbidity burden*" or ((multiple or coexisting or co-existing or concurrent or con-current or comorbid* or co-morbid* or several*) adj2 (disease* or illness* or condition* or diagnosis* or morbid* or patholog*))) AND ("household*" or "coresiden*" or "co-residen*" or ((household or family or domestic or residen*) adj2 (composition* or member* or characterist* or dweller*)) or "neighborhood*" or "neighbourhood*" or "small area*" or "small-area*" or "place" or "communit*" or "census tracts" or "census block group" or "zip code*" or "post code*" or "electoral ward*" or "data zone*" or "output area*" or "lower super output area*" or "municipalit*" or "provinc*" or "socioeconomic" or "socio-economic" or "social capital" or "inequalit*" or "disparit*" or "income" or "wealth" or "financial problem*" or "financial difficult*" or "vocation*" or "training opportunit*" or "employment opportunit*" or "occupation* opportunit*" or "job opportunit*" or "job insecurit" or "education* opportunit*" or "education* achiev*" or "education* quality" or "fast-food" or "fast food" or "healthy food" or "social determinant" or "wider determinant" or "neighbourhood disorder" or "overcrowding" or "public housing" or "population density" or "urban" or "ghetto" or "rural" or "slum" or "estate" or "poverty area*" or "physical environment*" or "built environment*" or "living standard*" or "family structure" or "family breakdown" or "family disintegrat*" or "single parent*" or "housing tenure" or "Townsend" or "Carstairs" or "Index of Multiple Deprivation" or "Nam-Powers" or "Hollingshead Index" or "Breadline Britain Index" or "inverse care law" or "Singh ind*" or "Yost ind*" or "Kolak ind*" or "social determinants of health" or "neighbourhood archetype*" or "segregation indic*" or "ecological inversion" or "ecological fallacy" or "ecological bias" or "depriv*" or "impoverish*" or "disadvantage*" or "community cohesion" or "community network" or

For peer review only

	"community support" or "transport* quality" or "transport* services" or "transport link*" or "public transport*" or "travel time" or "community resource access*" or "walkability" or "public space" or "green space" or "open space" or "recreation*" or "leisure activit*" or "food outlet*" or "food environment" or "air pollution" or "particulate matter" or "nitrogen dioxide" or "sulphur dioxide" or "sulfur dioxide" or "carbon monoxide" or "crime*") AND ("hospital admission*" or "hospitalisation" or "hospitalization" or "emergency admission*" or "unscheduled admission*" or "unplanned admission*" or "emergency department" or "accident and emergency")<br>Publication date: after 01 January 2000
Sociological Abstracts (623)	("multimorbidit*" or "multi-morbidit*" or "comorbidit*" or "co-morbidit*" or "polymorbidit*" or "poly-morbidit*" or "multicondition*" or "multi-condition*" or "multiple chronic condition*" or "morbidity burden*" or ((multiple or coexisting or co-existing or concurrent or con-current or comorbid* or co-morbid* or several*) adj2 (disease* or illness* or condition* or diagnosis* or morbid* or patholog*))) AND ("household*" or "coresiden*" or "co-residen*" or ((household or family or domestic or residen*) adj2 (composition* or member* or characterist* or dweller*)) or "neighborhood*" or "neighbourhood*" or "small area*" or "small-area*" or "place" or "communit*" or "census tracts" or "census block group" or "zip code*" or "post code*" or "electoral ward*" or "data zone*" or "output area*" or "lower super output area*" or "municipalit*" or "provinc*" or "socioeconomic" or "socio-economic" or "social capital" or "inequalit*" or "disparit*" or "income" or "wealth" or "financial problem*" or "financial difficult*" or "vocation*" or "training opportunit*" or "employment opportunit*" or "occupation* opportunit*" or "job opportunit*" or "job insecurit" or "education* opportunit*" or "education* achiev*" or "education* quality" or "fast-food" or "fast food" or "healthy food" or "social determinant" or "wider determinant" or "neighbourhood disorder" or "overcrowding" or "public housing" or "population density" or "urban" or "ghetto" or "rural" or "slum" or "estate" or "poverty area*" or "physical environment*" or "built environment*" or "living standard*" or "family structure" or "family breakdown" or "family disintegrat*" or "single parent*" or "housing tenure" or "Townsend" or "Carstairs" or "Index of Multiple Deprivation" or "Nam-Powers" or "Hollingshead Index" or "Breadline Britain Index" or "inverse care law" or "Singh ind*" or "Yost ind*" or "Kolak ind*" or "social determinants of health" or "neighbourhood archetype*" or "segregation indic*" or "ecological inversion" or "ecological fallacy" or "ecological bias" or "depriv*" or "impoverish*" or "disadvantage*" or "community cohesion" or "community network" or "community support" or "transport* quality" or "transport* services" or "transport link*" or "public transport*" or "travel time" or "community resource access*" or "walkability" or "public space" or "green space" or "open space" or "recreation*" or "leisure activit*" or "food outlet*" or "food environment" or "air pollution" or "particulate matter" or "nitrogen dioxide" or "sulphur dioxide" or "sulfur dioxide" or "carbon monoxide" or "crime*") AND ("hospital admission*" or "hospitalisation" or "hospitalization" or "emergency admission*" or "unscheduled admission*" or "unplanned admission*" or "emergency department" or "accident and emergency")<br>Publication date: after 01 January 2000
Cochrane (0)	("multimorbidit*" or "multi-morbidit*" or "comorbidit*" or "co-morbidit*" or "polymorbidit*" or "poly-morbidit*" or "multicondition*" or "multi-condition*" or "multiple chronic condition*" or "morbidity burden*" or ((multiple or coexisting or co-existing or concurrent or con-current or comorbid* or co-morbid* or several*) adj2 (disease* or illness* or condition* or diagnosis* or morbid* or patholog*)))
OpenGrey (0)	("multimorbidit*" or "multi-morbidit*" or "comorbidit*" or "co-morbidit*" or "polymorbidit*" or "poly-morbidit*" or "multicondition*" or "multi-condition*" or "multiple chronic condition*" or "morbidity burden*" or ((multiple or coexisting or co-existing or concurrent or con-current or comorbid* or co-morbid* or several*) adj2 (disease* or illness* or condition* or diagnosis* or morbid* or patholog*)))

For peer review only

Table S2. Study methodological quality ratings: Newcastle-Ottawa Assessment (NOAS), adapted for cross-sectional studies

Source	Selection				Comparability based on design and analysis	Outcome		Total
	Representativeness of the sample	Sample size	Non-respondents	Ascertainment of exposure		Assessment of outcome	Statistical test	
Chung et al (2016)	Half*	*	*	*	*	Half*	*	6 (Medium)
Lu et al (2017)	Half*	*	*	Half*	*	Half*	*	6.5 (Medium)
Fisher et al (2021)	Half*	*	*	Half*	**	*	*	7 (High)
Mbuya-Bienge et al (2021)	*	*	*	*	*	*	Half*	6.5 (Medium)
Oureta et al (2013)	*	*	Half*	*	*	*	Half*	6.5 (Medium)
Pati et al (20 15)	Half*	Half*	Half*	Half*	*	Half*	*	4.5(Medium)
Payne et al (2013)	*	*	*	*	**	*	*	8 (High)
Stafford et al (2020)	Half*	Half*	half*	*	*	*	*	5.5 (Medium)
Tomita et al (2021)	*	*	*	*	**	Half*	*	7.5 (High)
Wang et al	Half*	*	Half*	Half*	**	Half*	*	6 (Medium)

Methodological score: high = 7-8, medium = 4-6, low = 0-3

For peer review only

Box S1. Risk of bias Newcastle-Ottawa Assessment (NOAS), adapted for this study and application to examination of cross-sectional studies

A study can be awarded a maximum of one point for each numbered item within the Selection and Exposure categories. A maximum of two points can be given for the Comparability category.

Selection (maximum 4)

1. Representativeness of the sample

a. Truly representative of the average in the target population (random sample or whole population) *

b. Somewhat representative of the average in the target population (purposive sampling of representative healthcare sector areas or evidence that the sample is representative of the source population) half*

c. Selected group of users

d. No description of the sampling strategy

2. Sample size

a. Justified and satisfactory *

b. Adequately powered to detect a difference (at least 10 events per variable in multivariate analyses)*

c. Not justified

3. Non-respondents

a. Comparability between respondents and non-respondents characteristics is established, and the response rate is satisfactory (>60%)*

b. The response rate is unsatisfactory, or the comparability between respondents and non-respondents is unsatisfactory

c. No description of the response rate or the characteristics of the responders and non-responders

4. Risk factor exposure assessment

a. Assessment of exposure including type and duration from electronic health record or administrative database*

b. Assessment of exposure from questionnaire *

c. No description

Comparability (maximum 2)

5. The subjects in different outcome groups are comparable, based on the study design or analysis. Confounding factors are adjusted for

a. Study controls for multimorbidity and no multimorbidity, including age and gender adjustment**

b. Study controls for multimorbidity and no multimorbidity, without additional variable adjustment*

c. Study does not control for multimorbidity

Outcome (maximum 2)

6. Assessment of the outcome (emergency department or unscheduled hospital admission)

a. Electronic health record or health payment record account of healthcare use *

b. Reported healthcare use from questionnaire or no described data source half*

7. Statistical test

a. The statistical test used to analyse the data is clearly described and appropriate, and the measurement of the association is presented as either an OR, CI and P value or a beta coefficient, SE, and P value*

b. The statistical test is not appropriate, not described, or incomplete

For peer review only

Checkilsts

1. Preferred Reporting Items for Systematic Reviews and Meta-Analyses (PRISMA) 2020 Checklist

Section and Topic	Item #	Checklist item	Location where item is reported
**TITLE**			
Title	1	Identify the report as a systematic review.	Methods – paragraph 1<br>(Page 4)
**ABSTRACT**			
Abstract	2	See the PRISMA 2020 for Abstracts checklist.	See checklist below
**INTRODUCTION**			
Rationale	3	Describe the rationale for the review in the context of existing knowledge.	Introduction – paragraphs 1-3<br>(Page 4)
Objectives	4	Provide an explicit statement of the objective(s) or question(s) the review addresses.	Introduction – paragraphs 2-3<br>(Page 4)
**METHODS**			
Eligibility criteria	5	Specify the inclusion and exclusion criteria for the review and how studies were grouped for the syntheses.	Methods – paragraph 3, Table 1<br>(Page 5)
Information sources	6	Specify all databases, registers, websites, organisations, reference lists and other sources searched or consulted to identify studies. Specify the date when each source was last searched or consulted.	Methods – paragraph 1<br>(Page 5)
Search strategy	7	Present the full search strategies for all databases, registers and websites, including any filters and limits used.	Table S1
Selection process	8	Specify the methods used to decide whether a study met the inclusion criteria of the review, including how many reviewers screened each record and each report retrieved, whether they worked independently, and if applicable, details of automation tools used in the process.	Methods – paragraph 4<br>(Page 4)
Data collection process	9	Specify the methods used to collect data from reports, including how many reviewers collected data from each report, whether they worked independently, any processes for obtaining or confirming data from study investigators, and if applicable, details of automation tools used in the process.	Methods – paragraph 6<br>(Page 5)
Data items	10a	List and define all outcomes for which data were sought. Specify whether all results that were compatible with each outcome domain in each study were sought (e.g. for all measures, time points, analyses), and if not, the methods used to decide which results to collect.	Methods – paragraph 6<br>(Page 5)
	10b	List and define all other variables for which data were sought (e.g. participant and intervention characteristics, funding sources). Describe any assumptions made about any missing or unclear information.	Methods – paragraph 6<br>(Page 5)
Study risk of bias assessment	11	Specify the methods used to assess risk of bias in the included studies, including details of the tool(s) used, how many reviewers assessed each study and whether they worked independently, and if applicable, details of automation tools used in the process.	Methods – paragraph 5<br>(Page 5)
Effect measures	12	Specify for each outcome the effect measure(s) (e.g. risk ratio, mean difference) used in the synthesis or presentation of results.	Methods – paragraph 7

For peer review only

Section and Topic	Item #	Checklist item	Location where item is reported
			(Page 6)
Synthesis methods	13a	Describe the processes used to decide which studies were eligible for each synthesis (e.g. tabulating the study intervention characteristics and comparing against the planned groups for each synthesis (item #5)).	Methods – paragraph 7 (Page 6)
	13b	Describe any methods required to prepare the data for presentation or synthesis, such as handling of missing summary statistics, or data conversions.	Methods – paragraph 7 (Page 6)
	13c	Describe any methods used to tabulate or visually display results of individual studies and syntheses.	Methods – paragraph 7 (Page 6)
	13d	Describe any methods used to synthesize results and provide a rationale for the choice(s). If meta-analysis was performed, describe the model(s), method(s) to identify the presence and extent of statistical heterogeneity, and software package(s) used.	Methods – paragraph 7 (Page 5)
	13e	Describe any methods used to explore possible causes of heterogeneity among study results (e.g. subgroup analysis, meta-regression).	Methods – paragraph 7 (Page 5)
	13f	Describe any sensitivity analyses conducted to assess robustness of the synthesized results.	Not applicable
Reporting bias assessment	14	Describe any methods used to assess risk of bias due to missing results in a synthesis (arising from reporting biases).	Methods – paragraph 5 (Page 5)
Certainty assessment	15	Describe any methods used to assess certainty (or confidence) in the body of evidence for an outcome.	Methods – paragraph 7 (Page 5)
**RESULTS**			
Study selection	16a	Describe the results of the search and selection process, from the number of records identified in the search to the number of studies included in the review, ideally using a flow diagram.	Results – paragraph 1 (Page 6)
	16b	Cite studies that might appear to meet the inclusion criteria, but which were excluded, and explain why they were excluded.	Introduction – paragraph 2 (Page 4)
Study characteristics	17	Cite each included study and present its characteristics.	Results – paragraphs 2-4 and 7-12 (Pages 6-8), Table 2
Risk of bias in studies	18	Present assessments of risk of bias for each included study.	Results – paragraph 8 (Page 7), Table S2
Results of individual studies	19	For all outcomes, present, for each study: (a) summary statistics for each group (where appropriate) and (b) an effect estimate and its precision (e.g. confidence/credible interval), ideally using structured tables or plots.	Results – paragraphs 3-16 (Page 6-10), Table 3
Results of syntheses	20a	For each synthesis, briefly summarise the characteristics and risk of bias among contributing studies.	Results – paragraphs 8-16 (Page 7-10).

For peer review only

Section and Topic	Item #	Checklist item	Location where item is reported
	20b	Present results of all statistical syntheses conducted. If meta-analysis was done, present for each the summary estimate and its precision (e.g. confidence/credible interval) and measures of statistical heterogeneity. If comparing groups, describe the direction of the effect.	Results – paragraphs 9-16 (Page 8-10)
	20c	Present results of all investigations of possible causes of heterogeneity among study results.	Results – paragraphs 9-16 (Page 8-10)
	20d	Present results of all sensitivity analyses conducted to assess the robustness of the synthesized results.	Not applicable
Reporting biases	21	Present assessments of risk of bias due to missing results (arising from reporting biases) for each synthesis assessed.	Table S2
Certainty of evidence	22	Present assessments of certainty (or confidence) in the body of evidence for each outcome assessed.	Results – paragraphs 9-16 (Page 8-10)
**DISCUSSION**			
Discussion	23a	Provide a general interpretation of the results in the context of other evidence.	Discussion - paragraph 1 (Page 10)
	23b	Discuss any limitations of the evidence included in the review.	Discussion – paragraph 2 (Page 10)
	23c	Discuss any limitations of the review processes used.	Discussion – paragraph 2 (Page 10)
	23d	Discuss implications of the results for practice, policy, and future research.	Discussion – paragraph 6 (Page 11-12)
**OTHER INFORMATION**			
Registration and protocol	24a	Provide registration information for the review, including register name and registration number, or state that the review was not registered.	Methods – paragraph 1 (Page 4)
	24b	Indicate where the review protocol can be accessed, or state that a protocol was not prepared.	Methods – paragraph 1 (Page 4)
	24c	Describe and explain any amendments to information provided at registration or in the protocol.	Not applicable
Support	25	Describe sources of financial or non-financial support for the review, and the role of the funders or sponsors in the review.	Declarations (Page 13)
Competing interests	26	Declare any competing interests of review authors.	Declarations (Page 13)
Availability of data, code and other materials	27	Report which of the following are publicly available and where they can be found: template data collection forms; data extracted from included studies; data used for all analyses; analytic code; any other materials used in the review.	Stable 2, Table 3, upplementary file

From: Page MJ, McKenzie JE, Bossuyt PM, Boutron I, Hoffmann TC, Mulrow CD, et al. The PRISMA 2020 statement: an updated guideline for reporting systematic reviews. BMJ 2021;372:n71. doi: 10.1136/bmj.n71

For peer review only

2. PRISMA 2020 for Abstracts Checklist

Section and Topic	Item #	Checklist item	Reported (Yes/No)
**TITLE**			
Title	1	Identify the report as a systematic review.	Background
**BACKGROUND**			
Objectives	2	Provide an explicit statement of the main objective(s) or question(s) the review addresses.	Methods
**METHODS**			
Eligibility criteria	3	Specify the inclusion and exclusion criteria for the review.	Methods
Information sources	4	Specify the information sources (e.g. databases, registers) used to identify studies and the date when each was last searched.	Methods (and main paper)
Risk of bias	5	Specify the methods used to assess risk of bias in the included studies.	No (main paper)
Synthesis of results	6	Specify the methods used to present and synthesise results.	Methods
**RESULTS**			
Included studies	7	Give the total number of included studies and participants and summarise relevant characteristics of studies.	No (main paper)
Synthesis of results	8	Present results for main outcomes, preferably indicating the number of included studies and participants for each. If meta-analysis was done, report the summary estimate and confidence/credible interval. If comparing groups, indicate the direction of the effect (i.e. which group is favoured).	Results
**DISCUSSION**			
Limitations of evidence	9	Provide a brief summary of the limitations of the evidence included in the review (e.g. study risk of bias, inconsistency and imprecision).	No (main paper)
Interpretation	10	Provide a general interpretation of the results and important implications.	Results
**OTHER**			
Funding	11	Specify the primary source of funding for the review.	No (main paper)
Registration	12	Provide the register name and registration number.	Methods

From: Page MJ, McKenzie JE, Bossuyt PM, Boutron I, Hoffmann TC, Mulrow CD, et al. The PRISMA 2020 statement: an updated guideline for reporting systematic reviews. BMJ 2021;372:n71. doi: 10.1136/bmj.n71
For more information, visit: http://www.prisma-statement

For peer review only

3. Synthesis Without Meta-analysis (SWiM) reporting items

**SWiM is intended to complement and be used as an extension to PRISMA**			
**SWiM reporting item**	**Item description**	**Page in manuscript where item is reported**	**Other***
Methods			
1 Grouping studies for synthesis	1a) Provide a description of, and rationale for, the groups used in the synthesis (e.g., groupings of populations, interventions, outcomes, study design)	Methods – paragraph 7 (Pages 5-6)	
	1b) Detail and provide rationale for any changes made subsequent to the protocol in the groups used in the synthesis		Not applicable
2 Describe the standardised metric and transformation methods used	Describe the standardised metric for each outcome. Explain why the metric(s) was chosen, and describe any methods used to transform the intervention effects, as reported in the study, to the standardised metric, citing any methodological guidance consulted	Results – paragraph 5-16 (Pages 7-10)	
3 Describe the synthesis methods	Describe and justify the methods used to synthesise the effects for each outcome when it was not possible to undertake a meta-analysis of effect estimates	Methods – paragraphs 5 and 7 (Pages 5-6)	
4 Criteria used to prioritise results for summary and synthesis	Where applicable, provide the criteria used, with supporting justification, to select the particular studies, or a particular study, for the main synthesis or to draw conclusions from the synthesis (e.g., based on study design, risk of bias assessments, directness in relation to the review question)	Results – paragraph 5-16 (Pages 7-10)	
5 Investigation of heterogeneity in reported effects	State the method(s) used to examine heterogeneity in reported effects when it was not possible to undertake a meta-analysis of effect estimates and its extensions to investigate heterogeneity	Results – paragraph 5-16 (Pages 7-10)	
6 Certainty of evidence	Describe the methods used to assess certainty of the synthesis findings		Not applicable
7 Data presentation methods	Describe the graphical and tabular methods used to present the effects (e.g., tables, forest plots, harvest plots).<br>Specify key study characteristics (e.g., study design, risk of bias) used to order the studies, in the text and any tables or graphs, clearly referencing the studies included	Table 2, Table 3,	
Results			
8 Reporting results	For each comparison and outcome, provide a description of the synthesised findings, and the certainty of the findings. Describe the result in language that is consistent with the question the synthesis addresses, and indicate which studies contribute to the synthesis	Results – paragraph 5-16 (Pages 7-10)	
Discussion			
9 Limitations of the synthesis	Report the limitations of the synthesis methods used and/or the groupings used in the synthesis, and how these affect the conclusions that can be drawn in relation to the original review question	Discussion – paragraph 2 (Page 10)	

For peer review only
